# Supplementary material for: Effects of bright light therapy for depression during pregnancy: a randomised, double-blind controlled trial
Source: BMJ Open. 2020 Oct 28;10(10):e038030. doi: 10.1136/bmjopen-2020-038030 (PMC7594358; doi:10.1136/bmjopen-2020-038030)
Supplement: Supplementary data [file bmjopen-2020-038030supp001.pdf]

Supplementary Table 1. Photobiological characterizations of light therapy in both treatment arms.

	BLT	DRLT
Cyanopic irradiance (μW · cm^{-2})	578.7	2.24
Melanopic irradiance (μW · cm^{-2})	891	5.53
Chloropic irradiance (μW · cm^{-2})	1032.3	7.23
Erythropic irradiance (μW · cm^{-2})	1212.3	11.37
Rhodopic irradiance (μW · cm^{-2})	16.61	16.61

BLT = bright light therapy; DRLT = dim red light therapy

Supplementary Table 2. Observed median SIGH-SAD, HAM-D and EPDS scores with ranges and number of participants over the course of the study for both treatment arms.

Measure	T0	T0+1	T0+2	T0+3	T0+4	T0+5	T1	T2	T3	P1
**SIGH-SAD**										
**BLT (mdn, range, N)**	27 (14-44; 33)	16.5 (1-33; 30)	16 (2-43; 29)	15 (0-41; 25)	18 (0-32; 25)	17.5 (1-37; 24)	15.5 (0-29; 26)	13 (1-26; 25)	11 (0-29; 17)	8 (1-23; 20)
**DRLT (mdn, range, N)**	26.5 (13-42; 34)	19 (8-33; 31)	17 (2-35; 27)	18 (4-30; 29)	15 (3-28; 24)	16 (2-31; 25)	13 (2-34; 25)	11.5 (1-26; 24)	9.5 (1-31; 14)	8 (0-28; 25)
**HAM-D**										
**BLT (mdn, range, N)**	16 (7-29; 33)	9 (0-25; 30)	9 (1-30; 29)	8 (0-28; 25)	10 (0-22; 25)	10 (0-21; 24)	7.5 (0-20; 26)	8 (0-17; 25)	5 (0-16; 17)	3 (0-11; 20)
**DRLT (mdn, range, N)**	18 (4-29; 34)	10 (3-20; 31)	9 (1-22; 27)	9 (2-20; 29)	8 (0-18; 24)	8 (1-20; 25)	6 (1-18; 25)	4.5 (0-20; 24)	4 (0-15; 14)	4 (0-19; 25)
**EPDS**										
**BLT (mdn, range, N)**	16 (7-25; 31)	11 (3-23; 26)	11 (0-23; 26)	10 (0-19; 21)	8 (0-25; 23)	7 (0-18; 23)	9.5 (1-18; 26)	8.5 (0-15; 18)	8.5 (1-24; 16)	7 (0-13; 22)

**DRLT (mdn, range, N)**	16 (3-25; 34)	12 (6-19; 28)	12 (3-20; 25)	11.5 (3-21; 24)	10 (1-18; 24)	10 (2-19; 23)	6.5 (1-22; 24)	6 (0-21; 23)	4 (1-10; 12)	7 (0-18; 26)

BLT = bright light therapy; DRLT = dim red light therapy; SIGH- SAD = Structured Interview Guide for the Hamilton Depression Scale – Seasonal Affective Disorder version; HAM-D = Hamilton Rating Scale for Depression; EPDS = Edinburgh Postnatal Depression Scale; T0 = baseline, before treatment; T0+1, T0+2 ... T0+5 = weeks during intervention period; T1 = end of treatment; T2 = 3 weeks after end of treatment; T3 = 10 weeks after end of treatment; P1 = 2 months postpartum; mdn = median

Supplementary Table 3. Effects of allocation on the course of depressive symptoms through the intervention period and follow-up (until two months postpartum): sensitivity analyses.

	**β (95% CI) of intervention***	**β (95% CI) of follow-up****
**Adjusted analysis[a]**		
**SIGH-SAD**	-0.27 (-1.70, 1.15)	-0.24 (-1.68, 1.20)
**HAM-D**	0.10 (-0.51, 0.72)	0.13 (-0.49, 0.75)
**EPDS**	0.27 (-0.36, 0.90)	0.25 (-0.38, 0.89)
**Data imputation[b]**		
**SIGH-SAD**	-0.45 (-1.44, 0.53)	-0.08 (-0.63, 0.46)
**HAM-D**	-0.09 (-0.63, 0.44)	0.06 (-0.25, 0.37)
**EPDS**	0.19 (-0.30, 0.68)	0.04 (-0.24, 0.32)
**Post-hoc analysis: high treatment compliance[c]**		
**SIGH-SAD**	-0.40 (-1.36, 0.55)	-0.32 (-0.88, 0.24)
**HAM-D**	-0.12 (-0.79, 0.54)	-0.06 (-0.43, 0.31)
**EPDS**	0.03 (-0.58, 0.65)	-0.05 (-0.40, 0.30)

**Post-hoc analysis: high symptom severity[d]**		
**SIGH-SAD**	-0.84 (-2.33, 0.65)	-0.20 (-1.14, 0.75)
**HAM-D**	-0.16 (-1.12, 0.87)	0.13 (-0.48, 0.73)
**EPDS**	-0.05 (-0.92, 0.82)	0.20 (-0.33, 0.74)

* From start of study until end of treatment; ** From start of study until follow-up 2 months postpartum; [a] Propensity score composed of psychiatric history, ethnicity, level of education, an unplanned pregnancy, maternal age, parity, gestational age, duration of actual depression and other psychiatric or psychotherapeutic treatment interventions; [b] Last observation carried forward; [c] <7 missed treatments; [d] Based on median split baseline SIGH-SAD scores
