# Supplementary material for: Effects of bright light therapy for depression during pregnancy: a randomised, double-blind controlled trial
Source: BMJ Open. 2020 Oct 28;10(10):e038030. doi: 10.1136/bmjopen-2020-038030 (PMC7594358; doi:10.1136/bmjopen-2020-038030)
Supplement: Author's manuscript [file bmjopen-2020-038030.draft_revisions.pdf]

BMJ Open

BMJ Open is committed to open peer review. As part of this commitment we make the peer review history of every article we publish publicly available.

When an article is published we post the peer reviewers' comments and the authors' responses online. We also post the versions of the paper that were used during peer review. These are the versions that the peer review comments apply to.

The versions of the paper that follow are the versions that were submitted during the peer review process. They are not the versions of record or the final published versions. They should not be cited or distributed as the published version of this manuscript.

BMJ Open is an open access journal and the full, final, typeset and author-corrected version of record of the manuscript is available on our site with no access controls, subscription charges or pay-per-view fees (http://bmjopen.bmj.com).

If you have any questions on BMJ Open's open peer review process please email info.bmjopen@bmj.com

BMJ Open

Effects of bright light therapy for depression during pregnancy: a randomized, double-blind controlled trial

Journal:	*BMJ Open*
Manuscript ID	bmjopen-2020-038030
Article Type:	Original research
Date Submitted by the Author:	26-Feb-2020
Complete List of Authors:	Bais, Babette; Erasmus Medical Center, Psychiatry<br>Kamperman, Astrid M.; Erasmus MC, Psychiatry<br>Bijma, Hilmar; Erasmus Medical Center, Obstetrics and Gynaecology<br>Hoogendijk, Witte; Erasmus Medical Center, Psychiatry<br>Souman, Jan; Signify NV, Lighting Applications<br>Knijff, Esther; Erasmus Medical Center, Psychiatry<br>Lambregtse-van den Berg, Mijke; Erasmus MC, Psychiatry; Erasmus MC, Child and Adolescent Psychiatry/Psychology
Keywords:	Depression & mood disorders < PSYCHIATRY, OBSTETRICS, PSYCHIATRY

SCHOLARONE™
Manuscripts

I, the Submitting Author has the right to grant and does grant on behalf of all authors of the Work (as defined in the below author licence), an exclusive licence and/or a non-exclusive licence for contributions from authors who are: i) UK Crown employees; ii) where BMJ has agreed a CC-BY licence shall apply, and/or iii) in accordance with the terms applicable for US Federal Government officers or employees acting as part of their official duties; on a worldwide, perpetual, irrevocable, royalty-free basis to BMJ Publishing Group Ltd ("BMJ") its licensees and where the relevant Journal is co-owned by BMJ to the co-owners of the Journal, to publish the Work in this journal and any other BMJ products and to exploit all rights, as set out in our licence.

The Submitting Author accepts and understands that any supply made under these terms is made by BMJ to the Submitting Author unless you are acting as an employee on behalf of your employer or a postgraduate student of an affiliated institution which is paying any applicable article publishing charge ("APC") for Open Access articles. Where the Submitting Author wishes to make the Work available on an Open Access basis (and intends to pay the relevant APC), the terms of reuse of such Open Access shall be governed by a Creative Commons licence – details of these licences and which Creative Commons licence will apply to this Work are set out in our licence referred to above.

Other than as permitted in any relevant BMJ Author's Self Archiving Policies, I confirm this Work has not been accepted for publication elsewhere, is not being considered for publication elsewhere and does not duplicate material already published. I confirm all authors consent to publication of this Work and authorise the granting of this licence.

For peer review only

Effects of bright light therapy for depression during pregnancy: a randomized, double-blind controlled trial

Babette Bais, MSc[1]*, Astrid M Kamperman, PhD[2], Hilmar H Bijma, MD, PhD[3], Witte JG Hoogendijk, MD, PhD[4], Jan L Souman, PhD[5], Esther Knijff, MD, PhD[6], Mijke P Lambregtse-van den Berg, MD, PhD[7]

[1] Department of Psychiatry, Erasmus University Medical Centre Rotterdam, Rotterdam, The Netherlands

[2] Epidemiological and Social Psychiatric Research Institute, Department of Psychiatry, Erasmus University Medical Centre Rotterdam, Rotterdam, The Netherlands

[3] Department of Obstetrics and Gynaecology, Erasmus University Medical Centre Rotterdam, Rotterdam, The Netherlands

[4] Department of Psychiatry, Erasmus University Medical Centre Rotterdam, Rotterdam, The Netherlands

[5] Department Lighting Applications, Signify Research, Eindhoven, The Netherlands;

[6] Department of Psychiatry, Erasmus University Medical Centre Rotterdam, Rotterdam, The Netherlands

[7] Department of Child and Adolescent Psychiatry/Psychology, department of Psychiatry, Erasmus University Medical Centre Rotterdam, Rotterdam, The Netherlands

*Corresponding author: P.O. Box 2040, 3000CA Rotterdam, The Netherlands; b.bais@erasmusmc.nl

For peer review only

Abstract

Objectives Approximately 11-13% of pregnant women suffer from depression. Bright light therapy (BLT) is a promising treatment, combining direct availability, sufficient efficacy, low costs and high safety for both mother and child. Here, we examined the effects of BLT on depression during pregnancy.

Design Randomized, double-blind controlled trial.

Setting Primary and secondary care in The Netherlands, from November 2016 to March 2019.

Participants 67 pregnant (12-32 weeks gestational age) with a DSM-5 diagnosis of depressive disorder.

Interventions Participants were randomly allocated to treatment with either BLT (9,000 lux, 5,000 K) or dim red light therapy (DRLT, 100 lux, 2,700 K), which is considered placebo. For six weeks, both groups were treated daily at home for 30 minutes upon awakening. Follow-up took place weekly during the intervention, after six weeks of therapy, three and ten weeks after treatment and two months postpartum.

Primary and secondary outcome measures Depressive symptoms were measured primarily with the Structured Interview Guide for the Hamilton Depression Scale – Seasonal Affective Disorder. Secondary measures were the Hamilton Rating Scale for Depression and the Edinburgh Postnatal Depression Scale. Changes in rating scale scores of these questionnaires over time were analysed using generalized linear mixed models.

Results Median depression scores decreased by 40.6-53.1% in the BLT group and by 50.9-66.7% in the DRLT group. We found no statistically significant difference in symptom change scores between BLT and DRLT. Sensitivity and post-hoc analyses did not change our findings.

Conclusions BLT and DRLT were both effective in reducing depressive symptoms in pregnant women with depression. More research is necessary to determine whether these responses represent true treatment effects, non-specific treatment responses, placebo effects or a combination hereof.

Trial Registration Bright Up, NTR5476, http://www.trialregister.nl

Strengths and limitations of this study

- We conducted various follow up measurements, including postpartum, to study the effects of withdrawal of treatment and to study whether treatment during pregnancy would protect against postpartum depression.

For peer review only

- The setting of treatment was within a real world setting.
- A strength of this study was the comprehensive assessment of side effects, as well as acceptability and satisfaction of treatment.
- An unforeseen lack of resources prevented us from including 150 participants, as we aimed to do according to our sample size calculation.
- Depressive symptoms during the study are assessed by questionnaires, rather than diagnostic criteria.

For peer review only

Introduction

Antepartum depression is a common and high impact disease, with approximately 11-13% of pregnant women suffering from depression [1]. Antepartum depression is not only seen in autumn and winter, but is a year-round phenomenon, with certain subgroups even showing more symptoms in summer [2]. Many risk factors for antepartum depression have been identified [3,4], inflammation is mentioned amongst others as possible cause [5,6]. Women who suffer from antepartum depression are more likely to suffer from postpartum depression as well [7]. Children who are exposed to maternal depression during pregnancy have a higher risk of adverse birth outcomes, such as prematurity and being small for gestational age [8,9]. Additionally, children show more often cognitive, emotional and behavioral problems in childhood, adolescence and adulthood [10,11] and they have a higher risk of suffering from depression later in life [12]. During pregnancy, fetal programming of the hypothalamus-pituitary-adrenal gland (HPA) axis takes place, which can be affected by maternal depression during pregnancy and may have long-lasting effects on stress response [13]. Possible mechanisms are 1) maternal cortisol crossing the placenta and thus increasing fetal cortisol levels, 2) placental secretion of corticotropin-releasing factor, which stimulates both maternal and fetal cortisol, and 3) reduced blood flow to the fetus, causing fetal growth restriction [8,14-17]. In addition, epigenetic programming takes place within the antepartum period, which influences not only the health of the (unborn) infant, but also that of following generations [18]. Therefore, early detection and treatment of antepartum depression is highly important for both mother and infant.

In non-pregnant women, guidelines propose psychotherapy, antidepressant medication or a combination of both as treatment. However, psychotherapy might not be readily available and the safety of maternal use of antidepressants, which cross the placenta, still remains to be established. The use of antidepressants is controversial, because of potential teratogenicity [19,20]. For example, increased risks have been found for persistent pulmonary hypertension of the neonate [21] and cardiovascular malformations [22]. Furthermore, pregnant women express a strong preference for non-pharmacologic treatment because of the possible harm for their unborn child [23,24]. Moreover, current adherence to national guidelines by midwives and gynaecologists is low [25] and international guidelines on the pharmacological treatment of antepartum depression are not consistent [26], which might result in unwanted variation in practice. Despite this, antidepressant use during pregnancy is increasing, not only

For peer review only

in the Netherlands [27,28], but in other European countries and the United States as well [29-31]. In the Netherlands, approximately 2-3% of pregnant women use antidepressants [28,32,33]. In the United States, this prevalence is approximately 6-7% [34-36], but could even be as high as 15% in some states [37]. Therefore, it is urgent and clinically relevant to investigate alternative approaches to treat antepartum depression, such as bright light therapy (BLT) [38].

Light synchronizes the suprachiasmatic nucleus (SCN), or the 'biological clock', with the environmental day-night rhythm [39]. Light hits the retina and intrinsically photosensitive retinal ganglion cells (ipRGCs) in the retina project, via the retino-hypthalamic tract to the SCN and thus influences circadian rhythm [39-41], which may indirectly benefit depressive symptoms [42]. However, not only do ipRGCs project to the SCN, but also directly to brain regions important in the regulation of mood, such as the medial amygdala and the lateral habenula [39-41].

Although BLT is the first-choice treatment for seasonal affective disorder, a condition of reoccurring depressions during fall and winter, with remissions in spring and summer [43,44], the effects of BLT have been shown both in seasonal affective disorder and in non-seasonal depression, which is not only shown by a Cochrane review [45], but also by more recent systematic reviews and meta-analyses [46-49]. An open trial of BLT in pregnant women showed improvement of mean depression ratings by 49% [50]. Two small randomized controlled trials showed significant improvement of depression among pregnant women exposed to BLT compared to placebo [51,52]. Although these results seem promising, the sample sizes of these studies were small, making them at risk for chance-findings [53].

In this study, we compared the effectiveness of BLT compared to placebo light among pregnant women with a depressive disorder in a larger randomized clinical trial. Moreover, we followed women until the postpartum period, to study whether treatment with light therapy during pregnancy might protect against postpartum depression. We hypothesized that daily treatment with six weeks of morning BLT will improve depressive symptoms during pregnancy.

Material and Methods

Design

For peer review only

This study was a randomized, double-blind, placebo-controlled clinical trial (Bright Up, NTR5476, http://www.trialregister.nl). A detailed protocol can be found elsewhere [54]. In short, the aim of the Bright Up study was to evaluate the effectiveness of BLT for pregnant women with a depressive disorder, compared to placebo light.

Participants

Eligible participants were pregnant women (12-32 weeks of gestational age, confirmed by ultrasound) diagnosed with a depressive disorder, confirmed by a Structured Clinical Interview for DSM disorders (SCID) by one trained assessor [55]. The specific inclusion and exclusion criteria are listed in Table 1. In the earlier published study protocol [54], we aimed to include women who were 12-18 weeks pregnant. For pragmatic reasons, in particular the fact that a substantial number of women was referred after 18 weeks of pregnancy, we later decided to widen our inclusion criteria to 12-32 weeks pregnancy.

In the Netherlands, maternity care for low-risk pregnancies is provided by midwives (primary care). High-risk pregnancies are cared for by gynaecologists in a general hospital (secondary care) or fetal-maternal medicine unit (tertiary care).

In this study, women were recruited not only via health care professionals, such as general practitioners, midwifes, gynaecologists, psychiatrists and psychologists, but also via (social) media. A complete flow-chart of the recruitment can be found in Figure 1.

Initially, we calculated the number of women to be included, based on the results and research methodology of previous studies [50,51,56]. We expected a true treatment effect in the range of a 10-15% symptom reduction over the full course of treatment, reflecting a small to medium effect size. To demonstrate this, with an α of 0.05 and a β of 0.8, a total sample size of 126 participants, 63 per arm was needed. To account for loss to follow up during and after treatment, we aimed at including 150 women. Power calculations were performed using GLIMMPSE 2.1.5. software [57]. Inclusion took place in The Netherlands and started on 9 November 2016 and lasted until 15 March 2019. By then, 67 women were included. However, due to limiting resources, we decided to stop the inclusion.

Patient and Public Involvement

For peer review only

No patients involved.

Ethics

All procedures performed involving human participants were in accordance with the ethical standards of the institutional and/or national research committee and with the 1964 Helsinki declaration and its later amendments or comparable ethical standards. Written informed consent was obtained from all participants. The study protocol and later amendments were approved by the medical ethical committee of the Erasmus University Medical Centre, Rotterdam, The Netherlands (registration number MEC-2015-731).

Blinding

Participants were blinded to allocation. Participants were informed that the study aimed to investigate the efficacy of different light colours. They were not informed that one treatment arm was considered placebo treatment. This was in accordance with approval of the medical ethical committee.

Outcome assessors were blinded to the allocation of the participants. Participants were asked not to share any details regarding their treatment towards the assessors. When blinding was broken, the assessor was replaced. The researcher performing the primary statistical analyses (AK) was blinded to the allocation. The field researcher (BB) was not blinded to the allocation for practical reasons. This researcher made sure lamps of the correct allocation were delivered to the participants. Also, this researcher asked participants about any side effects, keeping the independent assessors blinded to any adverse effects that might break the blinding, e.g. strained eyes, and answered any questions from the participants regarding their lamps.

At baseline, we asked about any expectations concerning the treatment with regards to their depressive symptoms. Women could choose whether they expected a negative effect, a small negative effect, no effect, a small positive effect or a positive effect. After the intervention period, the participants were asked whether they were aware of their allocation.

Light therapy

For peer review only

Light treatment consisted of either active BLT (9,000 lux, color temperature 5,000 K) or dim red light therapy (DRLT, 100 lux, color temperature 2,700 K). The photobiological characterizations of these treatments are shown in Supplementary Table 1. The original lamps were adjusted in the factory where these are produced (EnergyUp HF3419/01, Philips, Eindhoven, The Netherlands). To ensure that participants are exposed to the same light intensity, the output of the lamps was fixed. For the control condition, the standard LED's in the lamp were replaced by LED's with a lower intensity and a different color temperature. The lamps in the control condition were positioned at the same distance from the participant as in the experimental condition.

The active light therapy was shown to be effective in other studies [51,52,56,58]. DRLT can be considered to be biologically inactive and thus as placebo treatment [45]. In line with two previous RCT's among pregnant women, we chose six weeks of daily light exposure [51,52].

The lamps were delivered at the participants' home by one researcher (BB) who was not blinded to the allocation of the participants. This researcher did not share anything about the allocation with the participants. After delivery of the lamps and instructions, participants commenced their daily treatment with light for 30 minutes within 30 minutes of habitual wake up time for a six weeks period. This took place at the participants' home. Participants sat in front of two lamps with a distance of approximately 40 cm (15.8 inches). They received a plastic ruler of this length to ensure of the correct distance. The light boxes were placed in a custom-made scaffolding, so that the height of the light boxes could be adjusted per person and glare was avoided. Apart from the light treatment, participants in both treatment arms received treatment as usual: women were free to visit their general practitioner, obstetric care provider or mental health care worker and start additional treatment, whenever they felt a need for this.

During the intervention period, self-reported compliance with the light treatment was checked weekly.

Method

A baseline interview was conducted by telephone by one researcher (BB). The baseline interview collected sociodemographic information (age, ethnicity, educational level, marital status, body mass index (BMI)), obstetric information (gestational age, whether the pregnancy was planned, parity), psychiatric information (substance use (smoking, alcohol, drugs), present and past medication use, present

For peer review only - http://bmjopen.bmj.com/site/about/guidelines.xhtml

depressive symptoms and psychiatric history) and information on somatic conditions. Also, participants were screened with the SCID for depressive disorder and various potential co-morbidities, such as generalized anxiety disorder and panic disorder. Previous depressive episodes were also assessed with the SCID. The general practitioner was contacted to verify present medication use and whether the participant met any exclusion criteria.

After baseline measurements and receiving written informed consent, the participants were randomly allocated to either receive BLT or DRLT in a 1:1 ratio. Randomization was done with the web-based computer-generated schedule ALEA (software for randomization in clinical trials, version 2.2) using random block sizes of 2-6 [59] by an independent researcher. Stratification factors were the use of any current antidepressant medication and the number of previous depressive episodes. The latter was dichotomized to three or less versus four or more [60].

Follow up took place at the following time points:

- weekly during the intervention period (T0+1, T0+2, etc.)
- after 6 weeks of treatment (T1)
- 3 weeks after end of treatment (T2)
- 10 weeks after end of treatment (T3)
- 2 months postpartum (P1)
- 6 months postpartum (P2)
- 18 months postpartum (P3)

At these time points, questionnaires were assessed and body material was collected. We collected urine, hair and cortisol from the participants, as can be found in our earlier published protocol [54].

This paper reports the short term effectiveness, i.e. up to two months postpartum.

Primary and secondary outcome measures

The primary outcome measure was the average change in depressive symptoms between the two groups, as measured by the Structured Interview Guide for the Hamilton Depression Scale – Seasonal Affective Disorder version (SIGH-SAD). Secondary outcome measures were these changes as measured

For peer review only - http://bmjopen.bmj.com/site/about/guidelines.xhtml

by the 17-item Hamilton Rating Scale for Depression (HAM-D) and the Edinburgh Depression Scale (EPDS).

In the earlier published protocol [54], we were primarily interested in the effects of light therapy on depressive symptoms. Secondarily, we were interested in the effects on various other outcomes, such as maternal hormonal levels, maternal sleep quality and infant outcomes. Depressive symptoms were measured by two questionnaires: the SIGH-SAD and the EPDS, with the HAM-D being part of the SIGH-SAD. Therefore, in the original protocol [54], we mentioned these two questionnaires together as the primary outcome, as opposed to the other outcomes (maternal hormonal levels and others). However, it is not technically possible to have more than one primary outcome. Our power calculation was based on the SIGH-SAD, which makes this our true primary outcome. The HAM-D and the EPDS are the secondary outcomes for this manuscript. In the current manuscript, we only report our findings regarding the depressive symptoms. We will report the other outcomes elsewhere.

The SIGH-SAD is a 29-item structured interview, consisting of 21 HAM-D items and 8 atypical items. We used the entire SIGH-SAD questionnaire as primary measure, since this is the current benchmark for assessment of depression severity in light therapy trials. We chose the original 17-item HAM-D questionnaire as a secondary measure, since it is more commonly used in clinical practice and research. Blinded assessors conducted the SIGH-SAD interviews (including HAM-D questions) by telephone weekly in the intervention period and at follow up.

The EPDS is a structured 10-item questionnaire and was used as a self-report measure of depression during pregnancy and postpartum [61]. Items are scored with a value 0-3, resulting in a sum score of 0-30 [61]. The EPDS was developed for the detection of postpartum depression, but has been validated for screening depression during pregnancy as well [62]. The EPDS was assessed weekly in the intervention period and at follow up. Participants received a link by e-mail to fill out the questionnaire.

Side effects, acceptability and satisfaction

During the intervention period, participants were asked weekly about any possible side effects.

Acceptability was assessed by asking participants about their subjective treatment experiences after the intervention period. Women could choose whether they experienced a negative effect, a small negative

For peer review only

effect, no effect, a small positive effect or a positive effect. Women were asked how easy or difficult they could implement the therapy in their daily schedule and how easy or difficult the lamp was in use: very difficult, difficult, neutral, easy or very easy. Women could answer whether they found the light therapy very unpleasant, unpleasant, neutral, pleasant or very pleasant. Women were asked whether they would like to use the light therapy outside of the study (yes/no). Finally, women were asked how likely they would recommend light therapy to others on a scale of 1 to 10.

Confounders

The baseline interview collected information on various confounders, such as sociodemographic, obstetric and psychiatric information and information on somatic conditions (see Method for further specifications).

The participant's chronotype was assessed at inclusion with the Munich Chronotype Questionnaire (MCTQ), a structured 19-item self-report questionnaire [63], since evening types are more prone to depression compared to morning types [64,65]. The participant can be classified into one of seven chronotypes: extremely, moderately or slightly early, normal or slightly, moderately or extremely late. Sum scores range from 16 to 86, with low scores indicating extremely late chronotypes.

Statistical analysis

Continuous participant characteristics were summarized using mean and standard deviation (SD). Categorical variables, such as educational level, were summarized by count and percent. In line with the CONSORT statement, baseline differences between the two treatment arms were not tested [66].

For treatment effect analyses, we applied an intention-to-treat procedure, since none of the participants could switch to a different condition, and we included all observations of all participants until the study ended or the participant(s) dropped out of the study.

The primary outcome was changes in SIGH-SAD rating scale scores over time. Secondary outcomes were changes in HAM-D and EPDS rating scale scores over time. Analyses were conducted using general linear mixed modelling analyses. In a series of random-intercept models, we included time, allocation and time x allocation interaction-term as an effect measure of allocation on the course of

For peer review only

depression rating scale scores. The standardized baseline score was included in the model, since baseline depression severity is an important predictor for treatment outcome [67]. We studied the treatment effect for both the intervention period and follow-up period (two months postpartum).

Primary analyses were first crude, then adjusted. As adjusted primary analyses, we calculated propensity scores based on patient characteristics (psychiatric history, ethnicity, level of education, an unplanned pregnancy, maternal age, parity, gestational age, duration of actual depression and other psychiatric or psychotherapeutic treatment interventions). Next, we adjusted separately for chronotype and the month of treatment. By means of sensitivity analyses, we repeated the primary analyses with last observation carried forward data imputation. As post-hoc analyses, we repeated the crude analyses for women with good compliance (<7 missed treatments) and for women with most severe depressive symptomatology (based on median split baseline SIGH-SAD scores). Effect parameters were supplied with a 95% confidence interval (CI).

Additionally, we tested responders versus non-responders with Fisher's exact test, where response was defined as a ≥50% decrease to a final score of ≤8 on the 17-item HAM-D and ≤5 on the EPDS at the end of the intervention period.

Data was analyzed using SPSS 21.0 (IBM Corporation, Chicago, IL, USA). Statistical significance was defined as $p<.05$.

Results

Demographic and clinical characteristics

In total, 283 women were referred to the study. The majority of the participants (82%) was recruited via (social) media. Of these referrals, we included and randomized 67 women, with 33 allocated to BLT and 34 to DRLT. In total, eleven women dropped out during the study, of whom five in the BLT group. Ten women dropped out in the intervention period, one at ten weeks after treatment. Figure 1 shows a flow-chart of the entire study sample.

Table 2 shows the participant characteristics at the time of inclusion. At inclusion, the mean (SD) of the SIGH-SAD was 26.5 (7.2), of the 17-item HAM-D was 16.9 (5.3) and of the EPDS was 16.1 (4.8). Median scores were respectively 27, 17 and 16.

For peer review only

305 The most common comorbidity was anxiety (25.4%), followed by obsessive compulsive disorder (17.9%), PTSS (11.9%) and social phobia (11.9%). Various somatic comorbidities were reported, such as asthma, Guillain-Barré syndrome and fibromyalgia.

308 During the course of this study, as part of the care as usual, eleven additional women started with psychotherapy: three women in the intervention period, one after the intervention period during pregnancy and seven in the postpartum period. During the entire study, four additional women started with psychotropic medication: one woman started with an SSRI in the intervention period and one woman in the postpartum period (both sertraline), one with an antipsychotic (quetiapine) and one with a benzodiazepine (temazepam) postpartum. Of one participant, the dose of the SSRI was increased in the postpartum period (escitalopram).

Compliance

Self-reported compliance was somewhat higher in the BLT group, compared to the DRLT group. Amongst the women treated with BLT, eight women (24.2%) never missed a treatment, in contrast to three women (8.8%) in the DRLT group. Sixteen women (48.5%) treated with BLT missed a maximum of six treatments, compared to twenty women (58.9% in the DRLT group. In both groups, two women missed seven to thirteen treatments in the intervention period. One woman treated with BLT and two with DRLT missed fourteen or more treatments. One woman treated with BLT and two with DRLT missed the final two weeks of treatment, the first one due to complete remission of her symptoms.

Maintaining blinding

Before treatment, three women (4.8%) did not expect any effect from light therapy for their depressive symptoms. All other participants expected a (small) positive effect. After treatment, one participant treated with BLT (3.0%) and three women in the group treated with DRLT (8.8%) thought they were treated with placebo treatment. All other women had no specific ideas about their allocation.

Treatment effect

For peer review only

Supplementary Table 2 shows the observed median SIGH-SAD, HAM-D and EPDS scores over the course of the study. In the women treated with BLT, median depression scores decreased by 42.6% (SIGH-SAD), 53.1% (HAM-D) and 40.6% (EPDS) in the intervention period. In the DRLT group, this was respectively 50.9%, 66.7% and 59.4%. After women stopped with light treatment, median scores continued to decrease for all questionnaires in both groups, three and ten weeks after treatment. At two months postpartum, women treated with BLT showed no increase in EPDS scores, whereas women treated with DRLT showed an increase in EPDS-scores. For both SIGH-SAD and HAM-D scores, a decrease was observed in both treatment arms.

We also calculated the median improvement scores without the baseline score. For women treated with BLT, these were 6.1% (SIGH-SAD), 16.7% (HAM-D) and 13.6% (EPDS). For women treated with DRLT, this was respectively 31.6%, 40% and 45.8%.

No statistically significant difference was found between the two treatment arms for the intervention period, nor for the entire study (Figure 2 and Table 3). Adjusted primary analyses, where we repeated our primary analyses adjusted for propensity scores, and sensitivity analyses with imputed data did not show any other findings (Supplementary Table 3). Adjustment for chronotype and month of treatment did not change our findings as well. Post-hoc analyses, where we repeated the analyses for women with higher treatment compliance and for women with higher symptom severity at baseline, did not show a statistically significant difference between the two treatment arms (Supplementary Table 3).

For the HAM-D, 13 participants in the BLT group and 17 participants in the DRLT group were considered responders. This was respectively 11 and 9 when measured with the EPDS. When we studied responders versus non-responders, we found no statistically significant differences for both HAM-D scores (p=.46) and EPDS scores (p=.60).

Side effects

For women treated with BLT, the most frequently reported side effect was headaches (30.3%), followed by sleep problems (12.1%) and nausea (6.1%). For women treated with DRLT, the most reported side effect was headaches (20.6%), followed by sleep problems (8.9%) and irritable eyes (5.9%). Side effects were not reported more often by women treated with BLT, compared to DRLT (p=0.52). Most side effects

For peer review only

were experienced for a maximum of three days. None of the women suffered from any (hypo)manic symptoms. We reduced the treatment duration for five women to 20 minutes daily due to their side effects. Interestingly, two women dropped out of the study due to side effects, but only in the DRLT group.

Acceptability and satisfaction

The majority of women experienced a (small) positive effect for their depressive symptoms (78.6% BLT; 61.5% DRLT; p=0.58). All participants found the lamp (very) easy in use. Most women found the light therapy pleasant (57.1% BLT; 50% DRLT; p=0.49). Twenty-six women reported that it was (very) easy to plan the light therapy in the morning (42.9% BLT; 53.8% DRLT; p=0.43). Thirty-two women reported that they would like to use light therapy outside of the study (57.1% BLT; 61.5% DRLT; p=0.79). On average, women reported it was likely they would recommend the light therapy to others (BLT mean 8.0, SD 1.3; DRLT mean 7.0, SD 2.7; p=0.08).

Discussion

We conducted a randomized controlled trial, evaluating the effectiveness of BLT in a sample of 67 pregnant women with major depressive disorder, compared to DRLT. We found no statistically significant difference between BLT and DRLT on depressive symptoms. Median depression scores decreased by 40.6-53.1% during the intervention in the women treated with BLT and by 50.9-66.7% in the women treated by DRLT.

Effects in the current study

This level of improvement is comparable to the studies by Oren *et al*. [50] and Corral *et al*. [68] who both found a reduction in mean depression scores of 49%. Oren *et al*. conducted an open trial in an antepartum population, whereas Corral *et al*. conducted a randomized controlled trial among women with a postpartum depression. Similar to Corral *et al*., we did not find a statistically significant difference between the effective and placebo conditions. The median improvement in the DRLT group can be explained by placebo effects, which could also be the case in the BLT group. A meta-analysis showed that the placebo response in antidepressant trials is approximately 68% [69], although this effect is not clear

For peer review only - http://bmjopen.bmj.com/site/about/guidelines.xhtml

yet in light therapy trials specifically. Secondly, the improvement in both groups can be explained by non-specific treatment effects such the structure offered by the study [42], the interaction with the researchers or increased awareness and self-care resulting from participating in the study. A systematic review on various studies in treating antepartum depression with a control condition showed that these trials often show a considerable reduction in symptom scores in both treatment arms [38]. Furthermore, it might be that symptoms decrease related to the course of pregnancy, spontaneous remission or regression to the mean. A meta-analysis showed that untreated depressive symptoms could decrease by 10-15%, on average [70]. However, untreated depression during pregnancy is an important predictor for postpartum depression [71]. We calculated the improvement of the depressive symptoms without the baseline scores, to study whether the improvement was especially notable in the first week of treatment. We found that the improvement was less, especially in the group treated with BLT, which may pinpoint to regression to the mean. For example, women may have the feeling of 'finally being heard', or feeling empowered about doing something about their symptoms, which may explain these findings.

Corral *et al*. mentioned that several participants commented positively on having 30 minutes of "quiet time" on a daily basis. Several of our participants mentioned this as well, which could reflect sinking into a state of more relaxation or more mindfulness which may have contributed to the improvement in both groups. Two meta-analyses showed that mindfulness-based therapy is an effective treatment for a variety of psychological problems [72,73]. An earlier pilot study and an open study of mindfulness also showed positive effects on mood specifically in pregnant women [74,75]. Corral *et al*. mentioned that many postpartum women are motivated to access recourses, such as psychological treatment, which could have exerted non-specific treatment effects. In their study however, no participant took part in any treatment during the study. In our study, several women started psychotherapy or antidepressant medication. However, adjustment for any intervention did however not change our findings.

Finally, it has been shown earlier in healthy volunteers that treatment with similar conditions as our placebo therapy might actually have some effects in melatonin suppression [76], which could explain why we actually see a decrease of symptoms in the DRLT group.

Differences with literature

For peer review only

The results of this study differ from the randomized controlled trials by Epperson *et al.* [51] and Wirz-Justice *et al*. [52], who did find superiority of bright light therapy over placebo in an antepartum population. Wirz-Justice *et al*. included only clinical patients and found that BLT had more effects in severe patients in their study. However, mean baseline SIGH-SAD score in the Wirz-Justice et al. and Epperson et al. studies were 27.7 and 28.1, respectively, which is not clinically relevant different from the present study (26.5). Additionally, we included baseline depression scores in our model, which did not change our findings. Also, post-hoc analyses, where we repeated the analyses for women with higher baseline severity, did not show any significant findings.

Both Epperson *et al*. and Wirz-Justice *et al*. treated their patients for 1 hour a day and within 10 minutes of habitual wake-up time, which is different from the present study. Thus far, no studies have been executed comparing the effectiveness of shorter versus longer exposure to bright light in non-seasonal depression. Possibly, more light output in the BLT group would be necessary to show superiority of BLT over DRLT in a pregnant population. However, other studies that treated patients for 30 minutes also did show a statistical significant difference between the effective and the placebo intervention in non-seasonal depression [45]. One must keep in mind that these studies have been done in non-pregnant populations and different – yet unknown – underlying mechanisms may play a part during pregnancy, such as hormonal fluctuations and a shift in social role.

Our placebo condition, in which the possible effect of DRLT could be questioned, is not a plausible explanation for not finding a statistically significant effect between the treatment arms. Epperson *et al*. used a placebo condition with 500 lux white light, which is questionable as a placebo, for white light of 100 lux is able to phase-shift human circadian rhythms [77]. Since this study found a significant improvement in women treated with BLT when compared to this placebo, it is unlikely that the settings of our placebo would explain failing to achieve a significant difference between the two treatment arms.

In the study by Corral *et al*., depression scores worsened after withdrawal of treatment, indicating that spontaneous remission would be less likely. However, in the present study, median depressions scores of all questionnaires continued to improve after withdrawal of treatment in both groups, indicating that spontaneous remission in both groups is a possible explanation for this finding.

For peer review only

444 *Strengths and limitations*

445 Internationally, we conducted the largest randomized controlled trial studying light therapy in pregnant women with a depression. Moreover, we conducted various follow up measurements, including postpartum, to study the effects of withdrawal of treatment and to study whether treatment during pregnancy would protect against postpartum depression. Another strength is using a single assessor to diagnose depression. Moreover, the setting of treatment was within a real world setting. Finally, a strength of this study was the comprehensive assessment of side effects, as well as acceptability and satisfaction of treatment.

452 The main limitation of our study was that an unforeseen lack of resources prevented us from including 150 participants, as we aimed to do according to our sample size calculation [54], which enables us to find only large treatment effects [54]. Another limitation is the fact that depressive symptoms during the study are assessed by questionnaires, rather than diagnostic criteria. Also, information about psychiatric history was collected via an interview and not through medical records, which may be influenced by recall bias. Moreover, various covariates are self-reported, such as BMI, substance use and medication. We noticed a different attrition rate at T3 (10 weeks after treatment) and P1 (2 months postpartum). At T3, this is due to the fact that more women treated with DRLT already gave birth at T3, which resulted in missing data. We do not have an explanation for the different attrition rate at P1. We cannot rule out the possibility that these differences in attrition might have impacted our follow-up results. However, our sensitivity analyses indicate our follow-up results to be robust for differences between the conditions and data imputation.

464 *Conclusions*

465 BLT has been shown effective in treating non-seasonal depression [45] and in women with antepartum depression as well [51,52]. In the present study, both BLT and DRLT showed improvement in pregnant women with a depressive disorder after 6 weeks of treatment. Given the very mild and short-lived side effects, the major improvement in a short time period, the high acceptability of the participants, the low costs and the direct availability, more studies to the effectiveness of BLT during pregnancy are warranted. It is important to determine whether the responses observed in the present study represent true treatment effects, non-specific treatment responses, placebo effects or a combination of these. This could be done

For peer review only

by studying biological outcomes, such as cortisol and melatonin levels, which might show a statistically significant difference between the two treatment arms irrespective of perceived symptoms of depression. Additionally, it might show an indication of the positive effects of light therapy on the circadian rhythm and its inhibiting effects on HPA-axis hyperactivity.

Acknowledgements

We would like to thank all participants for participating in the study. We would also like to thank all general practitioners, midwifes, gynaecologists, psychiatrists and psychologists for their help with the recruitment. We are grateful for all co-workers, students and assistants who contributed to the data collection in this study: Nina Molenaar, PhD, Marlies Brouwer, PhD, Leo Genet, MSc, Sophie de Droog, MSc, Sofie Koomen, MSc, Diewertje Houtman, MSc, Maria Zepeda, MSc, Nicolle Croes, MSc, Rianne Winters, MSc. Lisanne van Kesteren, BSc, Finn Stofkoper, BSc, Indira Schouten, MSc and Mieke Roukema, MSc.

Funding

MLB received funding from the 'Light, Cognition, Behaviour and Health' program of The Netherlands Organization for Health Research and Development (NWO; The Hague, The Netherlands), in collaboration with Signify Research (grant number 058-14-003) to fund the current study.

Competing interests

Author JS is employed by Signify Research. The lamps used in this study were provided by Signify Research.

Author's contributions

MLB is the project's principle investigator and initiator of the study, obtained funding and designed the study. BB was responsible for recruiting and counselling participants, running the study and collecting data. AK is the project's methodologist and executed the primary statistical analysis. HB and EK were involved in the recruitment of the study. JS provided support. AK, WH and MLB supervised the study. BB, AK and MLB prepared the original draft. All authors reviewed, edited and approved the final manuscript.

For peer review only

500

501 **Data availability statement**

502 The datasets used and/or analysed during the current study are available from the corresponding author

503 MLB on reasonable request.

504

505 **Word count**

506 5,289

507

508 **References**

509 1. Woody CA, Ferrari AJ, Siskind DJ, Whiteford HA, Harris MG. A systematic review and meta-
510 regression of the prevalence and incidence of perinatal depression. *J Affect Disord* 2017; **219**: 86-92.
511 2. Bais B, de Groot N, Grootendorst-van Mil NH, et al. Seasonality of depressive symptoms during
512 pregnancy. *Psychiatry Res* 2018; **268**: 257-62.
513 3. Lancaster CA, Gold KJ, Flynn HA, Yoo H, Marcus SM, Davis MM. Risk factors for depressive
514 symptoms during pregnancy: a systematic review. *Am J Obstet Gynecol* 2010; **202**(1): 5-14.
515 4. Räisänen S, Lehto SM, Nielsen HS, Gissler M, Kramer MR, Heinonen S. Risk factors for and
516 perinatal outcomes of major depression during pregnancy: a population-based analysis during 2002-
517 2010 in Finland. *BMJ Open* 2014; (11): DOI:10.1136/bmjopen-2014-004883.
518 5. Leff-Gelman P, Mancilla-Herrera I, Flores-Ramos M, et al. The Immune System and the Role of
519 Inflammation in Perinatal Depression. *Neurosci Bull* 2016; **32**(4): 398-420.
520 6. Osborne LM, Monk C. Perinatal depression--the fourth inflammatory morbidity of pregnancy?:
521 Theory and literature review. *Psychoneuroendocrinology* 2013; **38**(10): 1929-52.
522 7. Milgrom J, Gemmill AW, Bilszta JL, et al. Antenatal risk factors for postnatal depression: a large
523 prospective study. *J Affect Disord* 2008; **108**: 147-57.
524 8. Grote NK, Bridge JA, Gavin AR, Melville JL, Iyengar S, Katon WJ. A meta-analysis of depression
525 during pregnancy and the risk of preterm birth, low birth weight, and intrauterine growth restriction.
526 *Arch Gen Psychiatry* 2010; **67**(10): 1012-24.
527 9. Jarde A, Morais M, Kingston D, et al. Neonatal outcomes in women with untreated antenatal
528 depression compared with women without depression: a systematic review and meta-analysis. *JAMA*
529 *Psychiatry* 2016: DOI:10.1001/jamapsychiatry.2016.0934.
530 10. Talge NM, Neal C, Glover V, the Early Stress, Translational Research and Prevention Science
531 Network: Fetal and Neonatal Experience on Child and Adolescent Mental Health. Antenatal maternal
532 stress and long-term effects on child neurodevelopment: how and why? *J Child Psychol Psychiatry* 2007;
533 **48**(3-4): 245-61.
534 11. Hentges RF, Graham SA, Plamondon A, Tough S, Madigan S. A Developmental Cascade from
535 Prenatal Stress to Child Internalizing and Externalizing Problems. *J Pediatr Psychol* 2019.
536 12. Pearson RM, Evans J, Kounali D, et al. Maternal depression during pregnancy and the postnatal
537 period: risks and possible mechanisms for offspring depression at age 18 years. *JAMA Psychiatry* 2013;
538 **70**(12): 1312-9.
539 13. Glover V, O'Connor TG, O'Donnell K. Prenatal stress and the programming of the HPA axis.
540 *Neurosci Biobehav Rev* 2010; **35**(1): 17-22.

For peer review only

14. Dierckx B, Tulen JH, van den Berg MP, et al. Maternal psychopathology influences infant heart rate variability: Generation R Study. *Psychosom Med* 2009; **71**(3): 313-21.
15. Goedhart G, Vrijkotte TG, Roseboom TJ, van der Wal MF, Cuijpers P, Bonsel GJ. Maternal cortisol and offspring birthweight: results from a large prospective cohort study. *Psychoneuroendocrinology* 2010; **35**(5): 644-52.
16. Henrichs J, Schenk JJ, Roza SJ, et al. Maternal psychological distress and fetal growth trajectories: the Generation R Study. *Psychol Med* 2010; **40**(4): 633-43.
17. Zijlmans MA, Riksen-Walraven JM, de Weerth C. Associations between maternal prenatal cortisol concentrations and child outcomes: A systematic review. *Neurosci Biobehav Rev* 2015; **53**: 1-24.
18. Steegers EA, Barker ME, Steegers-Theunissen RP, Williams MA. Societal Valorisation of New Knowledge to Improve Perinatal Health: Time to Act. *Paediatr Perinat Epidemiol* 2016; **30**(2): 201-4.
19. Byatt N, Deligiannidis KM, Freeman MP. Antidepressant use in pregnancy: a critical review focused on risks and controversies. *Acta Psychiatr Scand* 2013; **127**(2): 94-114.
20. Hanley GE, Oberlander TF. The effect of perinatal exposures on the infant: antidepressants and depression. *Best Pract Res Clin Obstet Gynaecol* 2014; **28**(1): 37-48.
21. Kieler H, Artama M, Engeland A, et al. Selective serotonin reuptake inhibitors during pregnancy and risk of persistent pulmonary hypertension in the newborn: population based cohort study from the five Nordic countries. *BMJ* 2012; **344**: d8012.
22. Simoncelli M, Martin BZ, Berard A. Antidepressant use during pregnancy: a critical systematic review of the literature. *Curr Drug Saf* 2010; **5**(2): 153-70.
23. Battle CL, Salisbury AL, Schofield CA, Ortiz-Hernandez S. Perinatal antidepressant use: understanding women's preferences and concerns. *J Psychiatr Pract* 2013; **19**(6): 443-53.
24. Kothari A, de Laat J, Dulhunty JM, Bruxner G. Perceptions of pregnant women regarding antidepressant and anxiolytic medication use during pregnancy. *Australas Psychiatry* 2019; **27**(2): 117-20.
25. Molenaar NM, Brouwer ME, Duvekot JJ, et al. Antidepressants during pregnancy: Guideline adherence and current practice amongst Dutch gynaecologists and midwives. *Midwifery* 2018; **61**: 29-35.
26. Molenaar NM, Kamperman AM, Boyce P, Bergink V. Guidelines on treatment of perinatal depression with antidepressants: An international review. *Aust N Z J Psychiatry* 2018; **52**(4): 320-7.
27. Bakker MK, Kolling P, van den Berg PB, de Walle HE, de Jong van den Berg LT. Increase in use of selective serotonin reuptake inhibitors in pregnancy during the last decade, a population-based cohort study from the Netherlands. *Br J Clin Pharmacol* 2008; **65**(4): 600-6.
28. Molenaar NM, Lambregtse-van den Berg MP, Bonsel GJ. Dispensing patterns of selective serotonin reuptake inhibitors before, during and after pregnancy: a 16-year population-based cohort study from the Netherlands. *Arch Womens Ment Health* 2019.
29. Charlton RA, Jordan S, Pierini A, et al. Selective serotonin reuptake inhibitor prescribing before, during and after pregnancy: a population-based study in six European regions. *Bjog-Int J Obstet Gy* 2015; **122**(7): 1010-20.
30. Cooper WO, Willy ME, Pont SJ, Ray WA. Increasing use of antidepressants in pregnancy. *American Journal of Obstetrics and Gynecology* 2007; **196**(6): 544-5.
31. Jimenez-Solem E, Andersen JT, Petersen M, et al. Prevalence of Antidepressant Use during Pregnancy in Denmark, a Nation-Wide Cohort Study. *Plos One* 2013; **8**(4).
32. van Gelder MMHJ, Bos JHJ, Roeleveld N, de Jong-van den Berg LTW. Drugs associated with teratogenic mechanisms. Part I: dispensing rates among pregnant women in the Netherlands, 1998-2009. *Hum Reprod* 2014; **29**(1): 161-7.
33. Ververs T, Kaasenbrood H, Visser G, Schobben F, de Jong-van den Berg L, Egberts T. Prevalence and patterns of antidepressant drug use during pregnancy. *Eur J Clin Pharmacol* 2006; **62**(10): 863-70.

For peer review only

34. Ailes EC, Simeone RM, Dawson AL, Petersen EE, Gilboa SM. Using insurance claims data to identify and estimate critical periods in pregnancy: An application to antidepressants. *Birth Defects Res A Clin Mol Teratol* 2016; **106**(11): 927-34.

35. Andrade SE, Reichman ME, Mott K, et al. Use of selective serotonin reuptake inhibitors (SSRIs) in women delivering liveborn infants and other women of child-bearing age within the U.S. Food and Drug Administration's Mini-Sentinel program. *Arch Womens Ment Health* 2016; **19**(6): 969-77.

36. Taylor LG, Thelus Jean R, Gordon G, Fram D, Coster T. Development of a mother-child database for drug exposure and adverse event detection in the Military Health System. *Pharmacoepidemiol Drug Saf* 2015; **24**(5): 510-7.

37. Hanley GE, Mintzes B. Patterns of psychotropic medicine use in pregnancy in the United States from 2006 to 2011 among women with private insurance. *BMC Pregnancy Childbirth* 2014; **14**: 242.

38. van Ravesteyn LM, Lambregtse-van den Berg MP, Hoogendijk WJ, Kamperman AM. Interventions to treat mental disorders during pregnancy: A systematic review and multiple treatment meta-analysis. *PLoS One* 2017; **12**(3): e0173397.

39. Blume C, Garbazza C, Spitschan M. Effects of light on human circadian rhythms, sleep and mood. *Somnologie* 2019.

40. LeGates TA, Fernandez DC, Hattar S. Light as a central modulator of circadian rhythms, sleep and affect. *Nature Reviews Neuroscience* 2014; **15**(7): 443-54.

41. Prayag AS, Münch M, Aeschbach D, Chellappa SL, Gronfier C. Light modulation of human clocks, wake and sleep. *Clocks & Sleep* 2019; **1**: 193-208.

42. Germain A, Kupfer DJ. Circadian rhythm disturbances in depression. *Hum Psychopharmacol* 2008; **23**(7): 571-85.

43. Lam RW, Levitan RD. Pathophysiology of seasonal affective disorder: a review. *J Psychiatry Neurosci* 2000; **25**(5): 469-80.

44. Magnusson A, Boivin D. Seasonal affective disorder: an overview. *Chronobiol Int* 2003; **20**(2): 189-207.

45. Tuunainen A, Kripke DF, Endo T. Light therapy for non-seasonal depression. *Cochrane Database Syst Rev* 2004.

46. Golden RN, Gaynes BN, Ekstrom RD, et al. The efficacy of light therapy in the treatment of mood disorders: a review and meta-analysis of the evidence. *Am J Psychiatry* 2005; **162**(4): 656-62.

47. Al-Karawi D, Jubair L. Bright light therapy for nonseasonal depression: Meta-analysis of clinical trials. *J Affect Disord* 2016; **198**: 64-71.

48. Perera S, Eisen R, Bhatt M, et al. Light therapy for non-seasonal depression: systematic review and meta-analysis. *BJPsych Open* 2016; **2**(2): 116-26.

49. Martensson B, Pettersson A, Berglund L, Ekselius L. Bright white light therapy in depression: A critical review of the evidence. *J Affect Disord* 2015; **182**: 1-7.

50. Oren DA, Wisner KL, Spinelli M, et al. An open trial of morning light therapy for treatment of antepartum depression. *Am J Psychiatry* 2002; **159**(4): 666-9.

51. Epperson CN, Terman M, Terman JS, et al. Randomized clinical trial of bright light therapy for antepartum depression: preliminary findings. *J Clin Psychiatry* 2004; **65**(3): 421-5.

52. Wirz-Justice A, Bader A, Frisch U, et al. A randomized, double-blind, placebo-controlled study of light therapy for antepartum depression. *J Clin Psychiatry* 2011; **72**(7): 986-93.

53. Ioannidis JP. Why most published research findings are false. *PLoS Med* 2005; **2**(8): e124.

54. Bais B, Kamperman AM, van der Zwaag MD, et al. Bright light therapy in pregnant women with major depressive disorder: study protocol for a randomized, double-blind, controlled clinical trial. *BMC Psychiatry* 2016; **16**(1): 381.

55. First MB GM, Spitzer RL, Williams JBW. User's guide for the SCID-I. Structured clinical interview for DSM-IV TR axis I disorders (research version). New York: New York Psychiatric Institute; 2002.

For peer review only

56. Lieverse R, Van Someren EJ, Nielen MM, Uitdehaag BM, Smit JH, Hoogendijk WJ. Bright light treatment in elderly patients with nonseasonal major depressive disorder: a randomized placebo-controlled trial. *Arch Gen Psychiatry* 2011; **68**(1): 61-70.

57. Kreidler SM, Muller KE, Grunwald GK, et al. GLIMMPSE: Online Power Computation for Linear Models with and without a Baseline Covariate. *J Stat Softw* 2013; **54**(10).

58. Meesters Y, Dekker V, Schlangen LJ, Bos EH, Ruiter MJ. Low-intensity blue-enriched white light (750 lux) and standard bright light (10,000 lux) are equally effective in treating SAD. A randomized controlled study. *BMC Psychiatry* 2011; **11**: 17.

59. Project TAI. ALEA Randomisation Software (Version 2.2). Amsterdam: Netherlands Cancer Institute; 2006. p. http://tenalea.net/.

60. Bockting CL, Hollon SD, Jarrett RB, Kuyken W, Dobson K. A lifetime approach to major depressive disorder: The contributions of psychological interventions in preventing relapse and recurrence. *Clin Psychol Rev* 2015; **41**: 16-26.

61. Cox JL, Holden JM, Sagovsky R. Detection of postnatal depression. Development of the 10-item Edinburgh Postnatal Depression Scale. *Br J Psychiatry* 1987; **150**: 782-6.

62. Bergink V, Kooistra L, Lambregtse-van den Berg MP, et al. Validation of the Edinburgh Depression Scale during pregnancy. *J Psychosom Res* 2011; **70**(4): 385-9.

63. Roenneberg T, Wirz-Justice A, Merrow M. Life between clocks: daily temporal patterns of human chronotypes. *J Biol Rhythms* 2003; **18**(1): 80-90.

64. Merikanto I, Kronholm E, Peltonen M, Laatikainen T, Vartiainen E, Partonen T. Circadian preference links to depression in general adult population. *J Affect Disord* 2015; **188**: 143-8.

65. Merikanto I, Lahti T, Kronholm E, et al. Evening types are prone to depression. *Chronobiol Int* 2013; **30**(5): 719-25.

66. Schulz KF, Altman DG, Moher D, Group C. CONSORT 2010 Statement: updated guidelines for reporting parallel group randomised trials. *BMC Med* 2010; **8**: 18.

67. Friedman ES, Davis LL, Zisook S, et al. Baseline depression severity as a predictor of single and combination antidepressant treatment outcome: results from the CO-MED trial. *Eur Neuropsychopharmacol* 2012; **22**(3): 183-99.

68. Corral M, Wardrop AA, Zhang H, Grewal AK, Patton S. Morning light therapy for postpartum depression. *Arch Womens Ment Health* 2007; **10**(5): 221-4.

69. Rief W, Nestoriuc Y, Weiss S, Welzel E, Barsky AJ, Hofmann SG. Meta-analysis of the placebo response in antidepressant trials. *J Affect Disord* 2009; **118**(1-3): 1-8.

70. Posternak MA, Miller I. Untreated short-term course of major depression: a meta-analysis of outcomes from studies using wait-list control groups. *J Affect Disord* 2001; **66**(2-3): 139-46.

71. Yazici E, Kirkan TS, Aslan PA, Aydin N, Yazici AB. Untreated depression in the first trimester of pregnancy leads to postpartum depression: high rates from a natural follow-up study. *Neuropsychiatr Dis Treat* 2015; **11**: 405-11.

72. Khoury B, Lecomte T, Fortin G, et al. Mindfulness-based therapy: a comprehensive meta-analysis. *Clin Psychol Rev* 2013; **33**(6): 763-71.

73. Hofmann SG, Sawyer AT, Witt AA, Oh D. The effect of mindfulness-based therapy on anxiety and depression: A meta-analytic review. *J Consult Clin Psychol* 2010; **78**(2): 169-83.

74. Dimidjian S, Goodman SH, Felder JN, Gallop R, Brown AP, Beck A. An open trial of mindfulness-based cognitive therapy for the prevention of perinatal depressive relapse/recurrence. *Arch Womens Ment Health* 2015; **18**(1): 85-94.

75. Vieten C, Astin J. Effects of a mindfulness-based intervention during pregnancy on prenatal stress and mood: results of a pilot study. *Arch Womens Ment Health* 2008; **11**(1): 67-74.

76. Nagare R, Plitnick B, Figueiro MG. Effect of exposure duration and light spectra on nighttime melatonin suppression in adolescents and adults. *Light Res Technol* 2019; **51**(4): 530-43.

77. Zeitzer JM, Dijk DJ, Kronauer R, Brown E, Czeisler C. Sensitivity of the human circadian pacemaker to nocturnal light: melatonin phase resetting and suppression. *J Physiol* 2000; **526 Pt 3**: 695-702.

Table 1. Inclusion and exclusion criteria for the Bright Up Study.

**Inclusion criteria**	**Women**
	18-45 years of age
	12-32 weeks pregnant (as confirmed by ultrasound)
	Current DSM-5 diagnosis of depressive disorder (as assessed by the SCID*)
**Exclusion criteria**	Insufficient proficiency in Dutch or English
	Multiple pregnancy
	Current use of antidepressants shorter than 2 months
	Lifetime diagnosis of bipolar I or II disorder
	Any psychotic episode
	Current substance abuse
	Current primary anxiety disorder
	Recent history of suicide attempt
	Current shift-work
	Somatic and/or obstetric conditions that override study participation
	Previous treatment with BLT
	Eye condition (macular degeneration, eye diseases, recent eye surgery)

* SCID = Structured Clinical Interview for DSM disorders

Table 2. Overview of participant characteristics at inclusion.

	BLT (n=33)	DRLT (n=34)
**Age in years, mean (SD)**	31.9 (4.4)	31.9 (5.3)
**Gestational age in weeks, mean (SD)**	20.6 (6.2)	19.7 (6.3)

For peer review only

**Ethnicity**		
**Dutch**	27 (81.8%)	26 (76.5%)
**Other**	6 (19.2%)	8 (33.5%)
**Marital status**		
**Married or cohabiting**	33 (100%)	32 (94.1%)
**Committed relationship, not cohabiting**	0 (0%)	1 (2.9%)
**Single**	0 (0%)	1 (2.9%)
**Education**		
**Elementary or (pre-)vocational education**	11 (33.3%)	13 (38.2%)
**Higher professional education**	8 (24.2%)	11 (32.4%)
**(Pre-) academic education**	14 (42.4%)	10 (29.4%)
**Parity**		
**Nulliparous**	15 (45.5%)	20 (58.8%)
**Primiparous**	13 (39.4%)	9 (26.5%)
**Multiparous**	5 (15.2%)	5 (14.7%)
**BMI in kg/m^2 or st/ft^2, mean (SD)**	25.5 (4.5)	26.3 (5.4)
**Planned pregnancy**	22 (66.7%)	22 (64.7%)
**Antidepressant medication**	3 (9.1%)	5 (14.7%)
**Sleep medication**	3 (9.1%)	2 (5.9%)
**Psychotherapy**	14 (48.5%)	16 (47.1%)
**Comorbidities**		
**0**	17 (51.5%)	13 (38.2%)
**1**	9 (27.3%)	13 (38.2%)
**>1**	7 (21.2%)	8 (23.5%)
**Duration of depression in weeks, mean (SD)**	24.6 (16.9)	45.1 (121.9)
**Depressive episodes in past**		
**0**	12 (36.4%)	11 (32.4%)
**1**	9 (27.2%)	14 (41.2%)

For peer review only

**>1**	12 (36.4%)	9 (26.5%)
**Chronotype**		
**Early (extremely, moderately and slightly)**	20 (80%)	25 (92.6%)
**Normal**	1 (4%)	1 (3.7%)
**Late (extremely, moderately and slightly)**	4 (16%)	1 (3.7%)

BLT = bright light therapy; DRLT = dim red light therapy

Table 3. Effects of allocation on the course of depressive symptoms through the intervention period and follow-up (until two months postpartum): crude analysis

	**β (95% CI) of intervention***	**β (95% CI) of follow-up****
**SIGH-SAD**	-0.68 (-1.84, 0.49)	-0.16 (-0.82, 0.51)
**HAM-D**	-0.18 (-0.74, 0.37)	0.04 (-0.29, 0.37)
**EPDS**	0.01 (-0.51, 0.53)	-0.05 (-0.35, 0.24)

* From start of study until end of treatment; ** From start of study until follow-up 2 months postpartum

Figure legends

Figure 1. Flow-chart of the Bright Up study.

Figure 2. Estimated marginal means of depression scores in women with antepartum depression until two months postpartum. Shown are SIGH-SAD, HAM-D and EPDS scores. Black lines represent treatment with BLT, gray lines with DRLT. Bars represent standard error of the mean.

BLT = bright light therapy; DRLT = dim red light therapy; SIGH- SAD = Structured Interview Guide for the Hamilton Depression Scale – Seasonal Affective Disorder version; HAM-D = Hamilton Rating Scale for Depression; EPDS = Edinburgh Postnatal Depression Scale; T0 = baseline, before treatment; T0+1, T0+2 ... T0+5 = weeks during intervention period; T1 = end of treatment; T2 = 3 weeks after end of treatment; T3 = 10 weeks after end of treatment; P1 = 2 months postpartum

For peer review only

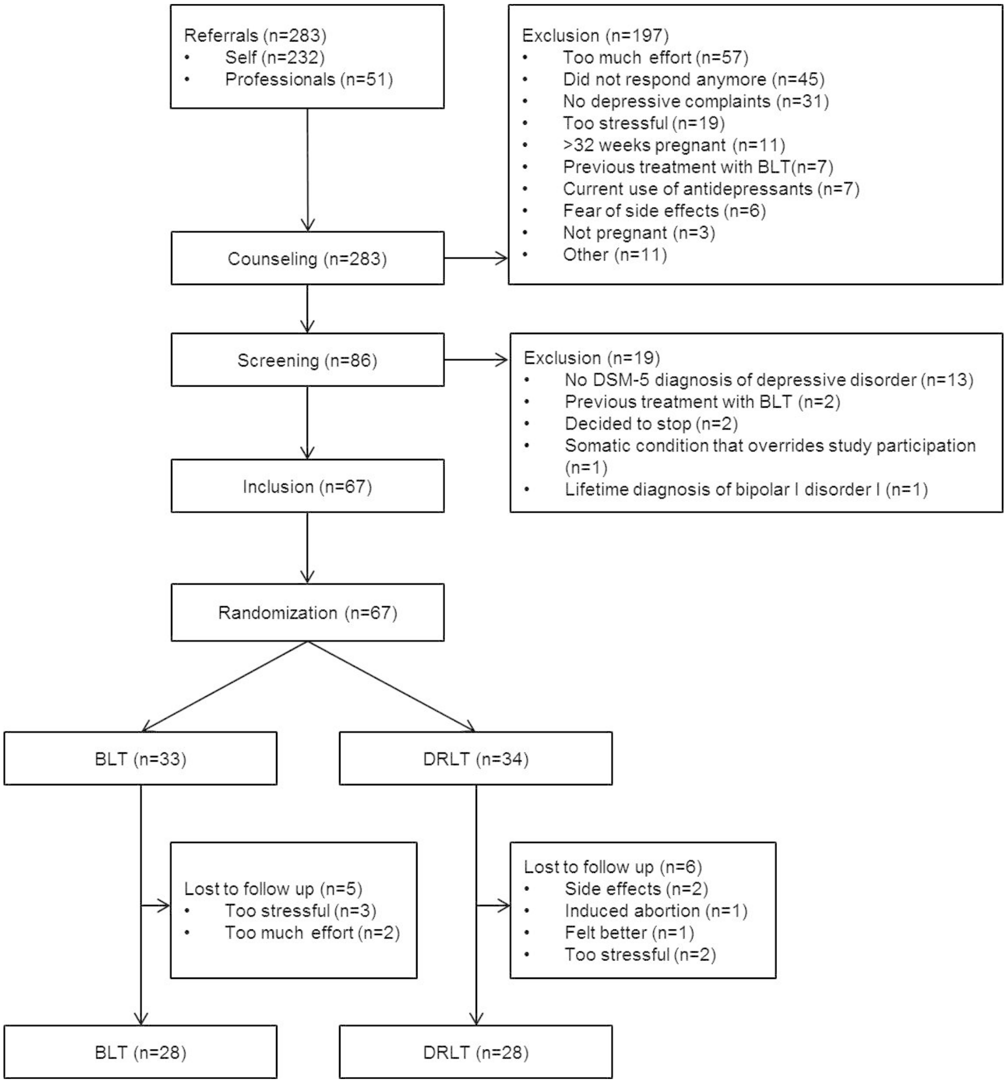

Flow-chart of the Bright Up study.

193x207mm (150 x 150 DPI)

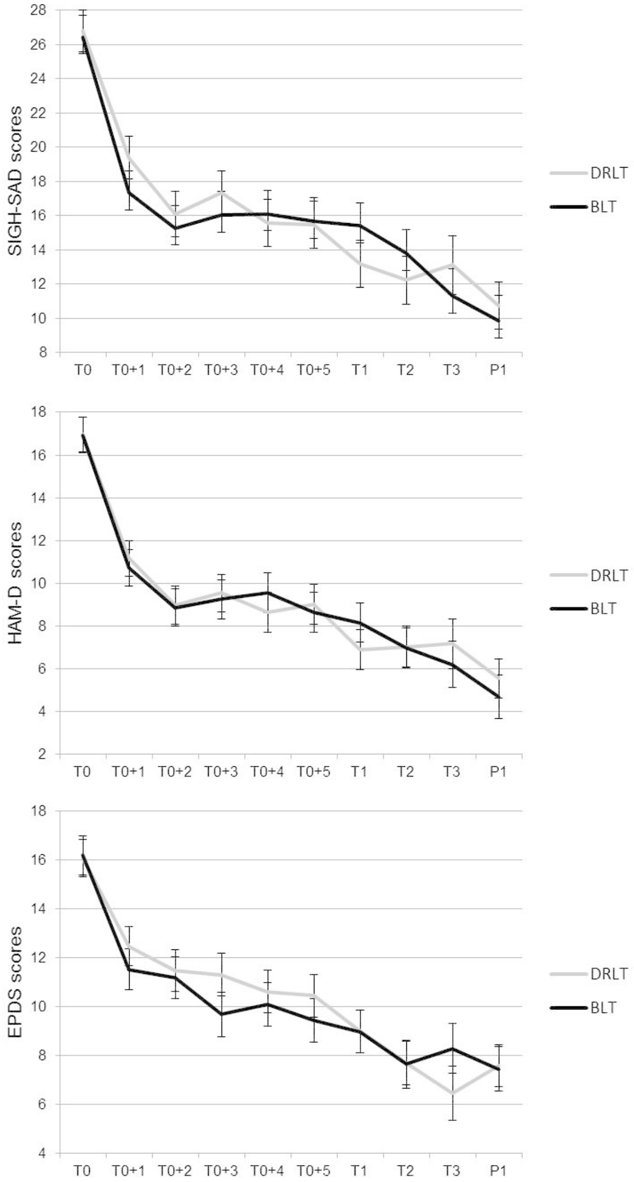

Estimated marginal means of depression scores in women with antepartum depression until two months postpartum. Shown are SIGH-SAD, HAM-D and EPDS scores. Black lines represent treatment with BLT, gray lines with DRLT. Bars represent standard error of the mean.
BLT = bright light therapy; DRLT = dim red light therapy; SIGH- SAD = Structured Interview Guide for the Hamilton Depression Scale – Seasonal Affective Disorder version; HAM-D = Hamilton Rating Scale for Depression; EPDS = Edinburgh Postnatal Depression Scale; T0 = baseline, before treatment; T0+1, T0+2 ... T0+5 = weeks during intervention period; T1 = end of treatment; T2 = 3 weeks after end of treatment; T3 = 10 weeks after end of treatment; P1 = 2 months postpartum

127x228mm (150 x 150 DPI)

Supplementary Table 1. Photobiological characterizations of light therapy in both treatment arms.

	BLT	DRLT
Cyanopic irradiance (μW · cm^{-2})	578.7	2.24
Melanopic irradiance (μW · cm^{-2})	891	5.53
Chloropic irradiance (μW · cm^{-2})	1032.3	7.23
Erythropic irradiance (μW · cm^{-2})	1212.3	11.37
Rhodopic irradiance (μW · cm^{-2})	16.61	16.61

BLT = bright light therapy; DRLT = dim red light therapy

For peer review only

Supplementary Table 2. Observed median SIGH-SAD, HAM-D and EPDS scores with ranges and number of participants over the course of the study for both treatment arms.

Measure	T0	T0+1	T0+2	T0+3	T0+4	T0+5	T1	T2	T3	P1
**SIGH-SAD**										
**BLT (mdn, range, N)**	27 (14-44; 33)	16.5 (1-33; 30)	16 (2-43; 29)	15 (0-41; 25)	18 (0-32; 25)	17.5 (1-37; 24)	15.5 (0-29; 26)	13 (1-26; 25)	11 (0-29; 17)	8 (1-23; 20)
**DRLT (mdn, range, N)**	26.5 (13-42; 34)	19 (8-33; 31)	17 (2-35; 27)	18 (4-30; 29)	15 (3-28; 24)	16 (2-31; 25)	13 (2-34; 25)	11.5 (1-26; 24)	9.5 (1-31; 14)	8 (0-28; 25)
**HAM-D**										
**BLT (mdn, range, N)**	16 (7-29; 33)	9 (0-25; 30)	9 (1-30; 29)	8 (0-28; 25)	10 (0-22; 25)	10 (0-21; 24)	7.5 (0-20; 26)	8 (0-17; 25)	5 (0-16; 17)	3 (0-11; 20)
**DRLT (mdn, range, N)**	18 (4-29; 34)	10 (3-20; 31)	9 (1-22; 27)	9 (2-20; 29)	8 (0-18; 24)	8 (1-20; 25)	6 (1-18; 25)	4.5 (0-20; 24)	4 (0-15; 14)	4 (0-19; 25)
**EPDS**										
**BLT (mdn, range, N)**	16 (7-25; 31)	11 (3-23; 26)	11 (0-23; 26)	10 (0-19; 21)	8 (0-25; 23)	7 (0-18; 23)	9.5 (1-18; 26)	8.5 (0-15; 18)	8.5 (1-24; 16)	7 (0-13; 22)

**DRLT (mdn, range, N)**	16 (3-25; 34)	12 (6-19; 28)	12 (3-20; 25)	11.5 (3-21; 24)	10 (1-18; 24)	10 (2-19; 23)	6.5 (1-22; 24)	6 (0-21; 23)	4 (1-10; 12)	7 (0-18; 26)

BLT = bright light therapy; DRLT = dim red light therapy; SIGH- SAD = Structured Interview Guide for the Hamilton Depression Scale – Seasonal Affective Disorder version; HAM-D = Hamilton Rating Scale for Depression; EPDS = Edinburgh Postnatal Depression Scale; T0 = baseline, before treatment; T0+1, T0+2 ... T0+5 = weeks during intervention period; T1 = end of treatment; T2 = 3 weeks after end of treatment; T3 = 10 weeks after end of treatment; P1 = 2 months postpartum; mdn = median

Supplementary Table 3. Effects of allocation on the course of depressive symptoms through the intervention period and follow-up (until two months postpartum): sensitivity analyses.

	**β (95% CI) of intervention***	**β (95% CI) of follow-up****
**Adjusted analysis[a]**		
**SIGH-SAD**	-0.24 (-1.68, 1.20)	-0.24 (-1.68, 1.20)
**HAM-D**	0.13 (-0.49, 0.75)	0.13 (-0.49, 0.75)
**EPDS**	0.25 (-0.38, 0.89)	0.25 (-0.38, 0.89)
**Data imputation[b]**		
**SIGH-SAD**	-0.45 (-1.44, 0.53)	-0.08 (-0.63, 0.46)
**HAM-D**	-0.09 (-0.63, 0.44)	0.06 (-0.25, 0.37)
**EPDS**	0.19 (-0.30, 0.68)	0.04 (-0.24, 0.32)
**Post-hoc analysis: high treatment compliance[c]**		
**SIGH-SAD**	-0.40 (-1.36, 0.55)	-0.32 (-0.88, 0.24)
**HAM-D**	-0.12 (-0.79, 0.54)	-0.06 (-0.43, 0.31)
**EPDS**	0.03 (-0.58, 0.65)	-0.05 (-0.40, 0.30)

For peer review only

**Post-hoc analysis: high symptom severity[d]**		
**SIGH-SAD**	-0.84 (-2.33, 0.65)	-0.20 (-1.14, 0.75)
**HAM-D**	-0.16 (-1.12, 0.87)	0.13 (-0.48, 0.73)
**EPDS**	-0.05 (-0.92, 0.82)	0.20 (-0.33, 0.74)

* From start of study until end of treatment; ** From start of study until follow-up 2 months postpartum; [a] Propensity score composed of psychiatric history, ethnicity, level of education, an unplanned pregnancy, maternal age, parity, gestational age, duration of actual depression and other psychiatric or psychotherapeutic treatment interventions; [b] Last observation carried forward; [c] <7 missed treatments; [d] Based on median split baseline SIGH-SAD scores

For peer review only

CONSORT 2010 checklist of information to include when reporting a randomised trial*

Section/Topic	Item No	Checklist item	Reported on page No
**Title and abstract**			
	1a	Identification as a randomised trial in the title	p. 1
	1b	Structured summary of trial design, methods, results, and conclusions (for specific guidance see CONSORT for abstracts)	p. 2
**Introduction**			
Background and objectives	2a	Scientific background and explanation of rationale	p. 4-5
	2b	Specific objectives or hypotheses	p. 5
**Methods**			
Trial design	3a	Description of trial design (such as parallel, factorial) including allocation ratio	p. 5
	3b	Important changes to methods after trial commencement (such as eligibility criteria), with reasons	p. 6
Participants	4a	Eligibility criteria for participants	p. 6; Table 1
	4b	Settings and locations where the data were collected	p. 6
Interventions	5	The interventions for each group with sufficient details to allow replication, including how and when they were actually administered	p. 7-8
Outcomes	6a	Completely defined pre-specified primary and secondary outcome measures, including how and when they were assessed	p. 8-11
	6b	Any changes to trial outcomes after the trial commenced, with reasons	n.a.
Sample size	7a	How sample size was determined	p. 6
	7b	When applicable, explanation of any interim analyses and stopping guidelines	n.a.
Randomisation:			
Sequence generation	8a	Method used to generate the random allocation sequence	p. 9
	8b	Type of randomisation; details of any restriction (such as blocking and block size)	p. 9
Allocation concealment mechanism	9	Mechanism used to implement the random allocation sequence (such as sequentially numbered containers), describing any steps taken to conceal the sequence until interventions were assigned	p. 7-9
Implementation	10	Who generated the random allocation sequence, who enrolled participants, and who assigned participants to interventions	p. 9
Blinding	11a	If done, who was blinded after assignment to interventions (for example, participants, care providers, those	p. 7

For peer review only

		assessing outcomes) and how	
	11b	If relevant, description of the similarity of interventions	p. 7-8
Statistical methods	12a	Statistical methods used to compare groups for primary and secondary outcomes	p. 11-12
	12b	Methods for additional analyses, such as subgroup analyses and adjusted analyses	p. 11-12
**Results**			
Participant flow (a diagram is strongly recommended)	13a	For each group, the numbers of participants who were randomly assigned, received intended treatment, and were analysed for the primary outcome	p. 12, Fig. 1, Supp. Table 2
	13b	For each group, losses and exclusions after randomisation, together with reasons	Fig. 1
Recruitment	14a	Dates defining the periods of recruitment and follow-up	p. 6
	14b	Why the trial ended or was stopped	p. 6
Baseline data	15	A table showing baseline demographic and clinical characteristics for each group	Table 2
Numbers analysed	16	For each group, number of participants (denominator) included in each analysis and whether the analysis was by original assigned groups	Sup. Table 2
Outcomes and estimation	17a	For each primary and secondary outcome, results for each group, and the estimated effect size and its precision (such as 95% confidence interval)	Sup. Table 2, Fig. 2, p.13-15, Table 3
	17b	For binary outcomes, presentation of both absolute and relative effect sizes is recommended	p. 14
Ancillary analyses	18	Results of any other analyses performed, including subgroup analyses and adjusted analyses, distinguishing pre-specified from exploratory	p. 14, Sup. Table 3
Harms	19	All important harms or unintended effects in each group (for specific guidance see CONSORT for harms)	p. 14
**Discussion**			
Limitations	20	Trial limitations, addressing sources of potential bias, imprecision, and, if relevant, multiplicity of analyses	p. 17-18
Generalisability	21	Generalisability (external validity, applicability) of the trial findings	p. 15-17
Interpretation	22	Interpretation consistent with results, balancing benefits and harms, and considering other relevant evidence	p. 15-17
**Other information**			
Registration	23	Registration number and name of trial registry	p. 2, 6
Protocol	24	Where the full trial protocol can be accessed, if available	p. 6
Funding	25	Sources of funding and other support (such as supply of drugs), role of funders	p. 19

*We strongly recommend reading this statement in conjunction with the CONSORT 2010 Explanation and Elaboration for important clarifications on all the items. If relevant, we also recommend reading CONSORT extensions for cluster randomised trials, non-inferiority and equivalence trials, non-pharmacological treatments, herbal interventions, and pragmatic trials. Additional extensions are forthcoming: for those and for up to date references relevant to this checklist, see www.consort-statement.org.

For peer review only - http://bmjopen.bmj.com/site/about/guidelines.xhtml

Effects of bright light therapy for depression during pregnancy: a randomized, double-blind controlled trial

Journal:	*BMJ Open*
Manuscript ID	bmjopen-2020-038030.R1
Article Type:	Original research
Date Submitted by the Author:	27-Aug-2020

Complete List of Authors:	Bais, Babette; Erasmus Medical Center, Psychiatry<br>Kamperman, Astrid M.; Erasmus MC, Psychiatry<br>Bijma, Hilmar; Erasmus Medical Center, Obstetrics and Gynaecology<br>Hoogendijk, Witte; Erasmus Medical Center, Psychiatry<br>Souman, Jan; Signify NV, Lighting Applications<br>Knijff, Esther; Erasmus Medical Center, Psychiatry<br>Lambregtse-van den Berg, Mijke; Erasmus Medical Center, Child and Adolescent Psychiatry/Psychology; Erasmus Medical Center, Psychiatry

**Primary Subject Heading**:	Mental health
Secondary Subject Heading:	Obstetrics and gynaecology
Keywords:	Depression & mood disorders < PSYCHIATRY, OBSTETRICS, PSYCHIATRY

SCHOLARONE™
Manuscripts

I, the Submitting Author has the right to grant and does grant on behalf of all authors of the Work (as defined in the below author licence), an exclusive licence and/or a non-exclusive licence for contributions from authors who are: i) UK Crown employees; ii) where BMJ has agreed a CC-BY licence shall apply, and/or iii) in accordance with the terms applicable for US Federal Government officers or employees acting as part of their official duties; on a worldwide, perpetual, irrevocable, royalty-free basis to BMJ Publishing Group Ltd ("BMJ") its licensees and where the relevant Journal is co-owned by BMJ to the co-owners of the Journal, to publish the Work in this journal and any other BMJ products and to exploit all rights, as set out in our licence.

The Submitting Author accepts and understands that any supply made under these terms is made by BMJ to the Submitting Author unless you are acting as an employee on behalf of your employer or a postgraduate student of an affiliated institution which is paying any applicable article publishing charge ("APC") for Open Access articles. Where the Submitting Author wishes to make the Work available on an Open Access basis (and intends to pay the relevant APC), the terms of reuse of such Open Access shall be governed by a Creative Commons licence – details of these licences and which Creative Commons licence will apply to this Work are set out in our licence referred to above.

Other than as permitted in any relevant BMJ Author's Self Archiving Policies, I confirm this Work has not been accepted for publication elsewhere, is not being considered for publication elsewhere and does not duplicate material already published. I confirm all authors consent to publication of this Work and authorise the granting of this licence.

For peer review only

Effects of bright light therapy for depression during pregnancy: a randomized, double-blind controlled trial

Babette Bais, MSc[1]*, Astrid M Kamperman, PhD[2], Hilmar H Bijma, MD, PhD[3], Witte JG Hoogendijk, MD, PhD[4], Jan L Souman, PhD[5], Esther Knijff, MD, PhD[6], Mijke P Lambregtse-van den Berg, MD, PhD[7]

[1] Department of Psychiatry, Erasmus University Medical Centre Rotterdam, Rotterdam, The Netherlands

[2] Epidemiological and Social Psychiatric Research Institute, Department of Psychiatry, Erasmus University Medical Centre Rotterdam, Rotterdam, The Netherlands

[3] Department of Obstetrics and Gynaecology, Erasmus University Medical Centre Rotterdam, Rotterdam, The Netherlands

[4] Department of Psychiatry, Erasmus University Medical Centre Rotterdam, Rotterdam, The Netherlands

[5] Department Lighting Applications, Signify Research, Eindhoven, The Netherlands;

[6] Department of Psychiatry, Erasmus University Medical Centre Rotterdam, Rotterdam, The Netherlands

[7] Department of Child and Adolescent Psychiatry/Psychology, department of Psychiatry, Erasmus University Medical Centre Rotterdam, Rotterdam, The Netherlands

*Corresponding author: P.O. Box 2040, 3000CA Rotterdam, The Netherlands; b.bais@erasmusmc.nl

For peer review only

Abstract

Objectives Approximately 11-13% of pregnant women suffer from depression. Bright light therapy (BLT) is a promising treatment, combining direct availability, sufficient efficacy, low costs, and high safety for both mother and child. Here, we examined the effects of BLT on depression during pregnancy.

Design Randomized, double-blind controlled trial.

Setting Primary and secondary care in The Netherlands, from November 2016 to March 2019.

Participants 67 pregnant women (12-32 weeks gestational age) with a DSM-5 diagnosis of depressive disorder.

Interventions Participants were randomly allocated to treatment with either BLT (9,000 lux, 5,000 K) or dim red light therapy (DRLT, 100 lux, 2,700 K), which is considered placebo. For six weeks, both groups were treated daily at home for 30 minutes upon awakening. Follow-up took place weekly during the intervention, after six weeks of therapy, three and ten weeks after treatment, and two months postpartum.

Primary and secondary outcome measures Depressive symptoms were measured primarily with the Structured Interview Guide for the Hamilton Depression Scale – Seasonal Affective Disorder. Secondary measures were the Hamilton Rating Scale for Depression and the Edinburgh Postnatal Depression Scale. Changes in rating scale scores of these questionnaires over time were analysed using generalized linear mixed models.

Results Median depression scores decreased by 40.6-53.1% in the BLT group and by 50.9-66.7% in the DRLT group. We found no statistically significant difference in symptom change scores between BLT and DRLT. Sensitivity and post-hoc analyses did not change our findings.

Conclusions BLT and DRLT both reduced depressive symptoms in pregnant women with depression. More research is necessary to determine whether these responses represent true treatment effects, non-specific treatment responses, placebo effects, or a combination hereof.

Trial Registration Bright Up, NTR5476, http://www.trialregister.nl

Strengths and limitations of this study

For peer review only

- We conducted various follow up measurements, including postpartum, to study the effects of withdrawal of treatment and to study whether treatment during pregnancy would protect against postpartum depression.
- The setting of treatment was within a real world setting.
- A strength of this study was the comprehensive assessment of side effects, as well as acceptability and satisfaction of treatment.
- An unforeseen lack of resources prevented us from including 150 participants, as we aimed to do according to our sample size calculation.
- Depressive symptoms during the study are assessed by questionnaires, rather than diagnostic criteria.

For peer review only

Introduction

Antepartum depression is a common and high impact disease, with approximately 11-13% of pregnant women suffering from depression [1]. Antepartum depression is not only seen in autumn and winter, but is a year-round phenomenon, with certain subgroups even showing more symptoms in summer [2]. Many risk factors for antepartum depression have been identified [3,4]. Possible causes for antepartum depression may include alterations in endocrine systems, such as the hypothalamus-pituitary-adrenal axis [5], and inflammation [6,7]. Women who suffer from antepartum depression are more likely to suffer from postpartum depression as well [8]. Children who are exposed to maternal depression during pregnancy have a higher risk of adverse birth outcomes, such as prematurity and being small for gestational age [9,10]. Additionally, children of mothers with antepartum depression show more often cognitive, emotional, and behavioral problems in childhood, adolescence, and adulthood [11,12] and they have a higher risk of suffering from depression later in life [13]. During pregnancy, fetal programming of the hypothalamus-pituitary-adrenal gland (HPA) axis takes place, which can be affected by maternal depression during pregnancy and may have long-lasting effects on stress response [14]. Possible mechanisms are 1) maternal cortisol crossing the placenta and thus increasing fetal cortisol levels, 2) placental secretion of corticotropin-releasing factor, which stimulates both maternal and fetal cortisol, and 3) reduced blood flow to the fetus, causing fetal growth restriction [9,15-18]. In addition, epigenetic programming takes place within the antepartum period, which influences not only the health of the (unborn) infant, but also that of following generations [19]. Therefore, early detection and treatment of antepartum depression is highly important for both mother and infant.

In non-pregnant women, guidelines propose psychotherapy, antidepressant medication, or a combination of both as treatment. However, psychotherapy might not be readily available and the safety of maternal use of antidepressants, which cross the placenta, still remains to be established. The use of antidepressants is controversial, because of potential teratogenicity [20,21]. For example, increased risks have been found for persistent pulmonary hypertension of the neonate [22] and cardiovascular malformations [23]. Furthermore, pregnant women express a strong preference for non-pharmacologic treatment because of the possible harm for their unborn child [24,25]. Moreover, current adherence to national guidelines by midwives and gynaecologists is low [26] and international guidelines on the

For peer review only

pharmacological treatment of antepartum depression are not consistent [27], which might result in unwanted variation in practice. Despite this, antidepressant use during pregnancy is increasing, not only in the Netherlands [28,29], but in other European countries and the United States as well [30-32]. In the Netherlands, approximately 2-3% of pregnant women use antidepressants [29,33,34]. In the United States, this prevalence is approximately 6-7% [35-37], but could even be as high as 15% in some states [38]. Therefore, it is urgent and clinically relevant to investigate alternative approaches to treat antepartum depression, such as bright light therapy (BLT) [39].

Light synchronizes the suprachiasmatic nucleus (SCN), or the 'biological clock', with the environmental day-night rhythm [40]. Light hits the retina and intrinsically photosensitive retinal ganglion cells (ipRGCs) in the retina project, via the retino-hypthalamic tract to the SCN and thus influences circadian rhythm [40-42], which may indirectly benefit depressive symptoms [43]. However, not only do ipRGCs project to the SCN, but also directly to brain regions important in the regulation of mood, such as the medial amygdala and the lateral habenula [40-42].

Although BLT is the first-choice treatment for seasonal affective disorder, a condition of reoccurring depressions during fall and winter, with remissions in spring and summer [44,45], the effects of BLT have been shown both in seasonal affective disorder and in non-seasonal depression, which is not only shown by a Cochrane review [46], but also by more recent systematic reviews and meta-analyses [47-50]. An open trial of BLT in pregnant women showed improvement of mean depression ratings by 49% [51]. Two small randomized controlled trials showed significant improvement of depression among pregnant women exposed to BLT compared to placebo [52,53]. Although these results seem promising, the sample sizes of these studies were small, making them at risk for chance-findings [54].

In this study, we compared the effectiveness of BLT compared to placebo light among pregnant women with a depressive disorder in a larger randomized clinical trial. Moreover, we followed women until the postpartum period, to study whether treatment with light therapy during pregnancy might protect against postpartum depression. We hypothesized that daily treatment with six weeks of morning BLT will improve depressive symptoms during pregnancy.

Material and Methods

For peer review only

Design

This study was a randomized, double-blind, placebo-controlled clinical trial (Bright Up, NTR5476, http://www.trialregister.nl). A detailed protocol can be found elsewhere [55]. In short, the aim of the Bright Up study was to evaluate the effectiveness of BLT for pregnant women with a depressive disorder, compared to placebo light.

Participants

Eligible participants were pregnant women (12-32 weeks of gestational age, confirmed by ultrasound) diagnosed with a depressive disorder, confirmed by a Structured Clinical Interview for DSM disorders (SCID) by one trained assessor [56]. The specific inclusion and exclusion criteria are listed in Table 1. In the earlier published study protocol [55], we aimed to include women who were 12-18 weeks pregnant. For pragmatic reasons, in particular the fact that a substantial number of women were referred after 18 weeks of pregnancy, we later decided to widen our inclusion criteria to 12-32 weeks pregnancy. In the Netherlands, maternity care for low-risk pregnancies is provided by midwives (primary care). High-risk pregnancies are cared for by gynaecologists in a general hospital (secondary care) or fetal-maternal medicine unit (tertiary care).

In this study, women were recruited not only via health care professionals, such as general practitioners, midwifes, gynaecologists, psychiatrists, and psychologists, but also via (social) media. A complete flow-chart of the recruitment can be found in Figure 1.

Initially, we calculated the number of women to be included, based on the results and research methodology of previous studies [51,52,57]. We expected a true treatment effect in the range of a 10-15% symptom reduction over the full course of treatment (6 weekly assessments), reflecting a small to medium effect size. To demonstrate this, with an α of 0.05 and a β of 0.8, time included as a continuous factor, a total sample size of 126 participants, 63 per arm was needed. To account for loss to follow up during and after treatment, we aimed at including 150 women. Power calculations were performed using GLIMMPSE 2.1.5. software [58]. Inclusion took place in The Netherlands and started on 9 November 2016 and lasted until 15 March 2019. By then, 67 women were included. However, due to limiting resources, we decided to stop the inclusion.

For peer review only

Patient and Public Involvement

No patients involved.

Ethics

All procedures performed involving human participants were in accordance with the ethical standards of the institutional and/or national research committee and with the 1964 Helsinki declaration and its later amendments or comparable ethical standards. Written informed consent was obtained from all participants. The study protocol and later amendments were approved by the medical ethical committee of the Erasmus University Medical Centre, Rotterdam, The Netherlands (registration number MEC-2015-731).

Blinding

Participants were blinded to allocation. Participants were informed that the study aimed to investigate the efficacy of different light colours. They were not informed that one treatment arm was considered placebo treatment. This was in accordance with approval of the medical ethical committee.

Outcome assessors were blinded to the allocation of the participants. Participants were asked not to share any details regarding their treatment towards the assessors. When blinding was broken, the assessor was replaced. The researcher performing the primary statistical analyses (AK) was blinded to the allocation. The field researcher (BB) was not blinded to the allocation for practical reasons. This researcher made sure lamps of the correct allocation were delivered to the participants. Also, this researcher asked participants about any side effects, keeping the independent assessors blinded to any adverse effects that might break the blinding, e.g. strained eyes, and answered any questions from the participants regarding their lamps.

At baseline, we asked about any expectations concerning the treatment with regards to their depressive symptoms. Women could choose whether they expected a negative effect, a small negative effect, no effect, a small positive effect or a positive effect. After the intervention period, the participants were asked whether they were aware of their allocation.

For peer review only

Light therapy

Light treatment consisted of either active BLT (9,000 lux, color temperature 5,000 K) or dim red light therapy (DRLT, 100 lux, color temperature 2,700 K). The photobiological characterizations of these treatments are shown in Supplementary Table 1. The original lamps were adjusted in the factory where these are produced (EnergyUp HF3419/01, Philips, Eindhoven, The Netherlands). To ensure that participants are exposed to the same light intensity, the output of the lamps was fixed. For the control condition, the standard LED's in the lamp were replaced by LED's with a lower intensity and a different color temperature. The lamps in the control condition were positioned at the same distance from the participant as in the experimental condition.

The active light therapy was shown to be effective in other studies [52,53,57,59]. DRLT can be considered to be biologically inactive and thus as placebo treatment [46]. In line with two previous RCT's among pregnant women, we chose six weeks of daily light exposure [52,53].

The lamps were delivered at the participants' home by one researcher (BB) who was not blinded to the allocation of the participants. This researcher did not share anything about the allocation with the participants. After delivery of the lamps and instructions, participants commenced their daily treatment with light for 30 minutes within 30 minutes of habitual wake up time for a six weeks period. This took place at the participants' home. Participants sat in front of two lamps with a distance of approximately 40 cm (15.8 inches). They received a plastic ruler of this length to ensure of the correct distance. The light boxes were placed in a custom-made scaffolding, so that the height of the light boxes could be adjusted per person and glare was avoided. Apart from the light treatment, participants in both treatment arms received treatment as usual: women were free to visit their general practitioner, obstetric care provider, or mental health care worker and start additional treatment, whenever they felt a need for this.

During the intervention period, self-reported compliance with the light treatment was checked weekly.

Method

A baseline interview was conducted by telephone by one researcher (BB). The baseline interview collected sociodemographic information (age, ethnicity, educational level, marital status, body mass index

For peer review only

(BMI)), obstetric information (gestational age, whether the pregnancy was planned, parity), psychiatric information (substance use (smoking, alcohol, drugs), present and past medication use, present depressive symptoms, psychiatric history), and information on somatic conditions. Also, participants were screened with the SCID for depressive disorder and various potential co-morbidities, such as generalized anxiety disorder and panic disorder. Previous depressive episodes were also assessed with the SCID. The general practitioner was contacted to verify present medication use and whether the participant met any exclusion criteria.

After baseline measurements and receiving written informed consent, the participants were randomly allocated to either receive BLT or DRLT in a 1:1 ratio. Randomization was done with the web-based computer-generated schedule ALEA (software for randomization in clinical trials, version 2.2) using random block sizes of 2-6 [60] by an independent researcher. Stratification factors were the use of any current antidepressant medication and the number of previous depressive episodes. The latter was dichotomized to three or less versus four or more [61].

Follow up took place at the following time points: weekly during the intervention period (T0+1, T0+2, etc.), after 6 weeks of treatment (T1), 3 weeks after end of treatment (T2), 10 weeks after end of treatment (T3), 2 months postpartum (P1), 6 months postpartum (P2), 18 months postpartum (P3).

At these time points, questionnaires were assessed and body material was collected. We collected urine, hair, and saliva from the participants, as can be found in our earlier published protocol [55].

This paper reports the short term effectiveness, i.e. up to two months postpartum.

Primary and secondary outcome measures

The primary outcome measure was the average change in depressive symptoms between the two groups, as measured by the Structured Interview Guide for the Hamilton Depression Scale – Seasonal Affective Disorder version (SIGH-SAD). Secondary outcome measures were these changes as measured by the 17-item Hamilton Rating Scale for Depression (HAM-D) and the Edinburgh Depression Scale (EPDS).

In the earlier published protocol [55], we were primarily interested in the effects of light therapy on depressive symptoms. Secondarily, we were interested in the effects on various other outcomes, such as

For peer review only

maternal hormonal levels, maternal sleep quality and infant outcomes. Depressive symptoms were measured by two questionnaires: the SIGH-SAD and the EPDS, with the original 17-item HAM-D being part of the SIGH-SAD, which consists of 21 HAM-D items and 8 atypical items. Therefore, in the original protocol [55], we mentioned these two questionnaires together as the primary outcome, as opposed to the other outcomes (maternal hormonal levels and others). However, it is not technically possible to have more than one primary outcome. Our power calculation was based on the SIGH-SAD, which makes this our true primary outcome. The HAM-D and the EPDS are the secondary outcomes for this manuscript. In the current manuscript, we only report our findings regarding the depressive symptoms. We will report the other outcomes elsewhere.

The SIGH-SAD is a 29-item structured interview, consisting of 21 HAM-D items and 8 atypical items. We used the entire SIGH-SAD questionnaire as primary measure, since this is the current benchmark for assessment of depression severity in light therapy trials. We chose the original 17-item HAM-D questionnaire as a secondary measure, since it is more commonly used in clinical practice and research. Blinded assessors conducted the SIGH-SAD interviews (including HAM-D questions) by telephone weekly in the intervention period and at follow up.

The EPDS is a structured 10-item questionnaire and was used as a self-report measure of depression during pregnancy and postpartum [62]. Items are scored with a value 0-3, resulting in a sum score of 0-30 [62]. The EPDS was developed for the detection of postpartum depression, but has been validated for screening depression during pregnancy as well [63]. The EPDS was assessed weekly in the intervention period and at follow up. Participants received a link by e-mail to fill out the questionnaire.

Side effects, acceptability and satisfaction

During the intervention period, participants were asked weekly about any possible side effects. Acceptability was assessed by asking participants about their subjective treatment experiences after the intervention period. Women could choose whether they experienced a negative effect, a small negative effect, no effect, a small positive effect, or a positive effect. Women were asked how easy or difficult they could implement the therapy in their daily schedule and how easy or difficult the lamp was in use: very difficult, difficult, neutral, easy, or very easy. Women could answer whether they found the light therapy

For peer review only

very unpleasant, unpleasant, neutral, pleasant, or very pleasant. Women were asked whether they would like to use the light therapy outside of the study (yes/no). Finally, women were asked how likely they would recommend light therapy to others on a scale of 1 to 10.

Baseline characteristics

The baseline interview collected information on various potential confounders, such as sociodemographic, obstetric, and psychiatric information, and information on somatic conditions (see Method for further specifications).

The participant's chronotype was assessed at inclusion with the Munich Chronotype Questionnaire (MCTQ), a structured 19-item self-report questionnaire [64], since evening types are more prone to depression compared to morning types [65,66]. The participant can be classified into one of seven chronotypes: extremely, moderately or slightly early, normal or slightly, moderately or extremely late. Sum scores range from 16 to 86, with low scores indicating extremely late chronotypes.

Statistical analysis

Continuous participant characteristics were summarized using mean and standard deviation (SD). Categorical variables, such as educational level, were summarized by count and percent. In line with the CONSORT statement, baseline differences between the two treatment arms were not tested [67].

For treatment effect analyses, we applied an intention-to-treat procedure, since none of the participants could switch to a different condition, and we included all observations of all participants until the study ended or the participant(s) dropped out of the study.

The primary outcome was changes in SIGH-SAD rating scale scores over time. Secondary outcomes were changes in HAM-D and EPDS rating scale scores over time. Analyses were conducted using general linear mixed modelling analyses. In a series of random-intercept models, we included time (continuous), allocation, and time x allocation interaction-term as an effect measure of allocation on the course of depression rating scale scores. The standardized baseline score was included in the model, since baseline depression severity is an important predictor for treatment outcome [68]. We studied the treatment effect for both the intervention period and follow-up period (two months postpartum).

For peer review only

Primary analyses were first crude, then adjusted. As adjusted primary analyses, we calculated propensity scores based on patient characteristics (psychiatric history, ethnicity, level of education, an unplanned pregnancy, maternal age, parity, gestational age, duration of actual depression, and other psychiatric or psychotherapeutic treatment interventions). Next, we adjusted separately for chronotype and the month of treatment. By means of sensitivity analyses, we repeated the primary analyses with last observation carried forward data imputation. As post-hoc analyses, we repeated the crude analyses for women with good compliance (<7 missed treatments) and for women with most severe depressive symptomatology (based on median split baseline SIGH-SAD scores). Effect parameters were supplied with a 95% confidence interval (CI).

Additionally, we tested responders versus non-responders with Fisher's exact test, where response was defined as a ≥50% decrease to a final score of ≤8 on the 17-item HAM-D and ≤5 on the EPDS at the end of the intervention period.

Data was analyzed using SPSS 21.0 (IBM Corporation, Chicago, IL, USA). Statistical significance was defined as p<.05.

Results

Demographic and clinical characteristics

In total, 283 women were referred to the study. The majority of the participants (82%) were recruited via (social) media. Of these referrals, we included and randomized 67 women, with 33 allocated to BLT and 34 to DRLT. In total, eleven women dropped out during the study, of whom five in the BLT group. Ten women dropped out in the intervention period, one at ten weeks after treatment. Figure 1 shows a flow-chart of the entire study sample.

Table 2 shows the participant characteristics at the time of inclusion. At inclusion, the mean (SD) of the SIGH-SAD was 26.5 (7.2), of the 17-item HAM-D was 16.9 (5.3) and of the EPDS was 16.1 (4.8). Median scores were respectively 27, 17 and 16.

The most common comorbidity was anxiety (25.4%), followed by obsessive compulsive disorder (17.9%), PTSS (11.9%), and social phobia (11.9%). Various somatic comorbidities were reported, such as asthma, Guillain-Barré syndrome, and fibromyalgia.

For peer review only

During the course of this study, as part of the care as usual, eleven additional women started with psychotherapy: three women in the intervention period, one after the intervention period during pregnancy, and seven in the postpartum period. During the entire study, four additional women started with psychotropic medication: one woman started with an SSRI in the intervention period and one woman in the postpartum period (both sertraline), one with an antipsychotic (quetiapine) and one with a benzodiazepine (temazepam) postpartum. Of one participant, the dose of the SSRI was increased in the postpartum period (escitalopram).

Compliance

Self-reported compliance was somewhat higher in the BLT group, compared to the DRLT group. Amongst the women treated with BLT, eight women (24.2%) never missed a treatment, in contrast to three women (8.8%) in the DRLT group. Sixteen women (48.5%) treated with BLT missed a maximum of six treatments, compared to twenty women (58.9%) in the DRLT group. In both groups, two women missed seven to thirteen treatments in the intervention period. One woman treated with BLT and two with DRLT missed fourteen or more treatments. One woman treated with BLT and two with DRLT missed the final two weeks of treatment, the first one due to complete remission of her symptoms.

Maintaining blinding

Before treatment, three women (4.8%) did not expect any effect from light therapy for their depressive symptoms. All other participants expected a (small) positive effect. After treatment, one participant treated with BLT (3.0%) and three women in the group treated with DRLT (8.8%) thought they were treated with placebo treatment. All other women had no specific ideas about their allocation.

Treatment effect

Supplementary Table 2 shows the observed median SIGH-SAD, HAM-D, and EPDS scores over the course of the study. In the women treated with BLT, median depression scores decreased by 42.6% (SIGH-SAD), 53.1% (HAM-D), and 40.6% (EPDS) in the intervention period. In the DRLT group, this was respectively 50.9%, 66.7%, and 59.4%. After women stopped with light treatment, median scores

For peer review only

continued to decrease for all questionnaires in both groups, three and ten weeks after treatment. At two months postpartum, women treated with BLT showed no increase in EPDS scores, whereas women treated with DRLT showed an increase in EPDS-scores. For both SIGH-SAD and HAM-D scores, a decrease was observed in both treatment arms.

We also calculated the median improvement scores without the baseline score. For women treated with BLT, these were 6.1% (SIGH-SAD), 16.7% (HAM-D), and 13.6% (EPDS). For women treated with DRLT, this was respectively 31.6%, 40%, and 45.8%.

No statistically significant difference was found between the two treatment arms for the intervention period, nor for the entire study (Figure 2 and Table 3). Adjusted primary analyses, where we repeated our primary analyses adjusted for propensity scores, and sensitivity analyses with imputed data did not show any other findings (Supplementary Table 3). Adjustment for chronotype and month of treatment did not change our findings as well. Post-hoc analyses, where we repeated the analyses for women with higher treatment compliance and for women with higher symptom severity at baseline, did not show a statistically significant difference between the two treatment arms (Supplementary Table 3).

For the HAM-D, 13 participants in the BLT group and 17 participants in the DRLT group were considered responders. This was respectively 11 and 9 when measured with the EPDS. When we studied responders versus non-responders, we found no statistically significant differences for both HAM-D scores (p=.46) and EPDS scores (p=.60).

Side effects

For women treated with BLT, the most frequently reported side effect was headaches (30.3%), followed by sleep problems (12.1%) and nausea (6.1%). For women treated with DRLT, the most reported side effect was headaches (20.6%), followed by sleep problems (8.9%) and irritable eyes (5.9%). Side effects were not reported more often by women treated with BLT, compared to DRLT (p=0.52). Most side effects were experienced for a maximum of three days. None of the women suffered from any (hypo)manic symptoms. We reduced the treatment duration for five women to 20 minutes daily due to their side effects. Interestingly, two women dropped out of the study due to side effects, but only in the DRLT group.

For peer review only

Acceptability and satisfaction

The majority of women experienced a (small) positive effect for their depressive symptoms (78.6% BLT; 61.5% DRLT; p=0.58). All participants found the lamp (very) easy in use. Most women found the light therapy pleasant (57.1% BLT; 50% DRLT; p=0.49). Twenty-six women reported that it was (very) easy to plan the light therapy in the morning (42.9% BLT; 53.8% DRLT; p=0.43). Thirty-two women reported that they would like to use light therapy outside of the study (57.1% BLT; 61.5% DRLT; p=0.79). On average, women reported it was likely they would recommend the light therapy to others (BLT mean 8.0, SD 1.3; DRLT mean 7.0, SD 2.7; p=0.08).

Discussion

We conducted a randomized controlled trial, evaluating the effectiveness of BLT in a sample of 67 pregnant women with major depressive disorder, compared to DRLT. We found no statistically significant difference between BLT and DRLT on depressive symptoms. Median depression scores decreased by 40.6-53.1% during the intervention in the women treated with BLT and by 50.9-66.7% in the women treated by DRLT.

Effects in the current study

This level of improvement is comparable to the studies by Oren *et al*. [51] and Corral *et al*. [69] who both found a reduction in mean depression scores of 49%. Oren *et al*. conducted an open trial in an antepartum population, whereas Corral *et al*. conducted a randomized controlled trial among women with a postpartum depression. Similar to Corral *et al*., we did not find a statistically significant difference between the effective and placebo conditions. The median improvement in the DRLT group can be explained by placebo effects, which could also be the case in the BLT group. A meta-analysis showed that the placebo response in antidepressant trials is approximately 68% [70], although this effect is not clear yet in light therapy trials specifically. Secondly, the improvement in both groups can be explained by non-specific treatment effects such the structure offered by the study [43], the interaction with the researchers, or increased awareness and self-care resulting from participating in the study. A systematic review on various studies in treating antepartum depression with a control condition showed that these trials often

For peer review only - http://bmjopen.bmj.com/site/about/guidelines.xhtml

show a considerable reduction in symptom scores in both treatment arms [39]. Furthermore, it might be that symptoms decrease related to the course of pregnancy, spontaneous remission, or regression to the mean. A meta-analysis showed that untreated depressive symptoms could decrease by 10-15%, on average [71]. However, untreated depression during pregnancy is an important predictor for postpartum depression [72]. We calculated the improvement of the depressive symptoms without the baseline scores, to study whether the improvement was especially notable in the first week of treatment. We found that the improvement was less, especially in the group treated with BLT, which may pinpoint to regression to the mean. For example, women may have the feeling of 'finally being heard', or feeling empowered about doing something about their symptoms, which may explain these findings.

Corral *et al*. mentioned that several participants commented positively on having 30 minutes of "quiet time" on a daily basis. Several of our participants mentioned this as well, which could reflect sinking into a state of more relaxation or more mindfulness which may have contributed to the improvement in both groups. Two meta-analyses showed that mindfulness-based therapy is an effective treatment for a variety of psychological problems [73,74]. An earlier pilot study and an open study of mindfulness also showed positive effects on mood specifically in pregnant women [75,76]. Corral *et al*. mentioned that many postpartum women are motivated to access recourses, such as psychological treatment, which could have exerted non-specific treatment effects. In their study however, no participant took part in any treatment during the study. In our study, several women started psychotherapy or antidepressant medication. However, adjustment for any intervention did however not change our findings.

Finally, it has been shown earlier in healthy volunteers that treatment with similar conditions as our placebo therapy might actually have some effects in melatonin suppression [77], which could explain why we actually see a decrease of symptoms in the DRLT group.

Differences with literature

The results of this study differ from the randomized controlled trials by Epperson *et al.* [52] and Wirz-Justice *et al.* [53], who did find superiority of bright light therapy over placebo in an antepartum population. Wirz-Justice *et al*. included only clinical patients and found that BLT had more effects in severe patients in their study. However, mean baseline SIGH-SAD score in the Wirz-Justice et al. and Epperson et al.

For peer review only

studies were 27.7 and 28.1, respectively, which were not clinically relevant different from the present study (26.5). Additionally, we included baseline depression scores in our model, which did not change our findings. Also, post-hoc analyses, where we repeated the analyses for women with higher baseline severity, did not show any significant findings.

Both Epperson *et al*. and Wirz-Justice *et al*. treated their patients for 1 hour a day and within 10 minutes of habitual wake-up time, which is different from the present study. Thus far, no studies have been executed comparing the effectiveness of shorter versus longer exposure to bright light in non-seasonal depression. Possibly, more light output in the BLT group would be necessary to show superiority of BLT over DRLT in a pregnant population. However, other studies that treated patients for 30 minutes also did show a statistical significant difference between the effective and the placebo intervention in non-seasonal depression [46]. One must keep in mind that these studies have been done in non-pregnant populations and different – yet unknown – underlying mechanisms may play a part during pregnancy, such as hormonal fluctuations and a shift in social role.

Our placebo condition, in which the possible effect of DRLT could be questioned, is not a plausible explanation for not finding a statistically significant effect between the treatment arms. Epperson *et al*. used a placebo condition with 500 lux white light, which is questionable as a placebo, for white light of 100 lux is able to phase-shift human circadian rhythms [78]. Since this study found a significant improvement in women treated with BLT when compared to this placebo, it is unlikely that the settings of our placebo would explain failing to achieve a significant difference between the two treatment arms.

In the study by Corral *et al*., depression scores worsened after withdrawal of treatment, indicating that spontaneous remission would be less likely. However, in the present study, median depressions scores of all questionnaires continued to improve after withdrawal of treatment in both groups, indicating that spontaneous remission in both groups is a possible explanation for this finding.

Strengths and limitations

Internationally, we conducted the largest randomized controlled trial studying light therapy in pregnant women with a depression. Moreover, we conducted various follow up measurements, including postpartum, to study the effects of withdrawal of treatment and to study whether treatment during

For peer review only

pregnancy would protect against postpartum depression. Another strength is using a single assessor to diagnose depression. Moreover, the setting of treatment was within a real world setting. Finally, a strength of this study was the comprehensive assessment of side effects, as well as acceptability and satisfaction of treatment.

The main limitation of our study was that an unforeseen lack of resources prevented us from including 150 participants, as we aimed to do according to our sample size calculation [55], which enables us to find only large treatment effects [55]. Another limitation is the fact that depressive symptoms during the study are assessed by questionnaires, rather than diagnostic criteria. Also, information about psychiatric history was collected via an interview and not through medical records, which may be influenced by recall bias. Moreover, various covariates are self-reported, such as BMI, substance use and medication. We noticed a different attrition rate at T3 (10 weeks after treatment) and P1 (2 months postpartum). At T3, this is due to the fact that more women treated with DRLT already gave birth at T3, which resulted in missing data. We do not have an explanation for the different attrition rate at P1. We cannot rule out the possibility that these differences in attrition might have impacted our follow-up results. However, our sensitivity analyses indicate our follow-up results to be robust for differences between the conditions and data imputation.

Conclusions

BLT has been shown effective in treating non-seasonal depression [46] and in women with antepartum depression as well [52,53]. In the present study, both BLT and DRLT showed improvement in pregnant women with a depressive disorder after 6 weeks of treatment. Given the very mild and short-lived side effects, the major improvement in a short time period, the high acceptability of the participants, the low costs, and the direct availability, more studies to the effectiveness of BLT during pregnancy are warranted. It is important to determine whether the responses observed in the present study represent true treatment effects, non-specific treatment responses, placebo effects, or a combination of these. This could be done by studying biological outcomes, such as cortisol and melatonin levels, which might show a statistically significant difference between the two treatment arms irrespective of perceived symptoms of depression. Additionally, it might show an indication of the positive effects of light therapy on the circadian rhythm and its inhibiting effects on HPA-axis hyperactivity.

For peer review only

Acknowledgements

We would like to thank all participants for participating in the study. We would also like to thank all general practitioners, midwifes, gynaecologists, psychiatrists and psychologists for their help with the recruitment. We are grateful for all co-workers, students and assistants who contributed to the data collection in this study: Nina Molenaar, PhD, Marlies Brouwer, PhD, Leo Genet, MSc, Sophie de Droog, MSc, Sofie Koomen, MSc, Diewertje Houtman, MSc, Maria Zepeda, MSc, Nicolle Croes, MSc, Rianne Winters, MSc. Lisanne van Kesteren, BSc, Finn Stofkoper, BSc, Indira Schouten, MSc and Mieke Roukema, MSc.

Funding

MLB received funding from the 'Light, Cognition, Behaviour and Health' program of The Netherlands Organization for Health Research and Development (NWO; The Hague, The Netherlands), in collaboration with Signify Research (grant number 058-14-003) to fund the current study.

Competing interests

Author JS is employed by Signify Research. The lamps used in this study were provided by Signify Research.

Author's contributions

MLB is the project's principle investigator and initiator of the study, obtained funding and designed the study. BB was responsible for recruiting and counselling participants, running the study and collecting data. AK is the project's methodologist and executed the primary statistical analysis. HB and EK were involved in the recruitment of the study. JS provided support. AK, WH and MLB supervised the study. BB, AK and MLB prepared the original draft. All authors reviewed, edited and approved the final manuscript.

Data availability statement

The datasets used and/or analysed during the current study are available from the corresponding author MLB on reasonable request.

For peer review only

Word count

5,372

References

1. Woody CA, Ferrari AJ, Siskind DJ, Whiteford HA, Harris MG. A systematic review and meta-regression of the prevalence and incidence of perinatal depression. *J Affect Disord* 2017; **219**: 86-92.

2. Bais B, de Groot N, Grootendorst-van Mil NH, et al. Seasonality of depressive symptoms during pregnancy. *Psychiatry Res* 2018; **268**: 257-62.

3. Lancaster CA, Gold KJ, Flynn HA, Yoo H, Marcus SM, Davis MM. Risk factors for depressive symptoms during pregnancy: a systematic review. *Am J Obstet Gynecol* 2010; **202**(1): 5-14.

4. Räisänen S, Lehto SM, Nielsen HS, Gissler M, Kramer MR, Heinonen S. Risk factors for and perinatal outcomes of major depression during pregnancy: a population-based analysis during 2002-2010 in Finland. *BMJ Open* 2014; (11): DOI:10.1136/bmjopen-2014-004883.

5. Seth S, Lewis AJ, Galbally M. Perinatal maternal depression and cortisol function in pregnancy and the postpartum period: a systematic literature review. *BMC Pregnancy Childbirth* 2016; **16**(1): 124.

6. Leff-Gelman P, Mancilla-Herrera I, Flores-Ramos M, et al. The Immune System and the Role of Inflammation in Perinatal Depression. *Neurosci Bull* 2016; **32**(4): 398-420.

7. Osborne LM, Monk C. Perinatal depression--the fourth inflammatory morbidity of pregnancy?: Theory and literature review. *Psychoneuroendocrinology* 2013; **38**(10): 1929-52.

8. Milgrom J, Gemmill AW, Bilszta JL, et al. Antenatal risk factors for postnatal depression: a large prospective study. *J Affect Disord* 2008; **108**: 147-57.

9. Grote NK, Bridge JA, Gavin AR, Melville JL, Iyengar S, Katon WJ. A meta-analysis of depression during pregnancy and the risk of preterm birth, low birth weight, and intrauterine growth restriction. *Arch Gen Psychiatry* 2010; **67**(10): 1012-24.

10. Jarde A, Morais M, Kingston D, et al. Neonatal outcomes in women with untreated antenatal depression compared with women without depression: a systematic review and meta-analysis. *JAMA Psychiatry* 2016: DOI:10.1001/jamapsychiatry.2016.0934.

For peer review only

532 11. Talge NM, Neal C, Glover V, the Early Stress, Translational Research and Prevention Science Network: Fetal and Neonatal Experience on Child and Adolescent Mental Health. Antenatal maternal stress and long-term effects on child neurodevelopment: how and why? *J Child Psychol Psychiatry* 2007; **48**(3-4): 245-61.

536 12. Hentges RF, Graham SA, Plamondon A, Tough S, Madigan S. A Developmental Cascade from Prenatal Stress to Child Internalizing and Externalizing Problems. *J Pediatr Psychol* 2019.

538 13. Pearson RM, Evans J, Kounali D, et al. Maternal depression during pregnancy and the postnatal period: risks and possible mechanisms for offspring depression at age 18 years. *JAMA Psychiatry* 2013; **70**(12): 1312-9.

541 14. Glover V, O'Connor TG, O'Donnell K. Prenatal stress and the programming of the HPA axis. *Neurosci Biobehav Rev* 2010; **35**(1): 17-22.

543 15. Dierckx B, Tulen JH, van den Berg MP, et al. Maternal psychopathology influences infant heart rate variability: Generation R Study. *Psychosom Med* 2009; **71**(3): 313-21.

545 16. Goedhart G, Vrijkotte TG, Roseboom TJ, van der Wal MF, Cuijpers P, Bonsel GJ. Maternal cortisol and offspring birthweight: results from a large prospective cohort study. *Psychoneuroendocrinology* 2010; **35**(5): 644-52.

548 17. Henrichs J, Schenk JJ, Roza SJ, et al. Maternal psychological distress and fetal growth trajectories: the Generation R Study. *Psychol Med* 2010; **40**(4): 633-43.

550 18. Zijlmans MA, Riksen-Walraven JM, de Weerth C. Associations between maternal prenatal cortisol concentrations and child outcomes: A systematic review. *Neurosci Biobehav Rev* 2015; **53**: 1-24.

552 19. Steegers EA, Barker ME, Steegers-Theunissen RP, Williams MA. Societal Valorisation of New Knowledge to Improve Perinatal Health: Time to Act. *Paediatr Perinat Epidemiol* 2016; **30**(2): 201-4.

554 20. Byatt N, Deligiannidis KM, Freeman MP. Antidepressant use in pregnancy: a critical review focused on risks and controversies. *Acta Psychiatr Scand* 2013; **127**(2): 94-114.

556 21. Hanley GE, Oberlander TF. The effect of perinatal exposures on the infant: antidepressants and depression. *Best Pract Res Clin Obstet Gynaecol* 2014; **28**(1): 37-48.

For peer review only

22. Kieler H, Artama M, Engeland A, et al. Selective serotonin reuptake inhibitors during pregnancy and risk of persistent pulmonary hypertension in the newborn: population based cohort study from the five Nordic countries. *BMJ* 2012; **344**: d8012.

23. Simoncelli M, Martin BZ, Berard A. Antidepressant use during pregnancy: a critical systematic review of the literature. *Curr Drug Saf* 2010; **5**(2): 153-70.

24. Battle CL, Salisbury AL, Schofield CA, Ortiz-Hernandez S. Perinatal antidepressant use: understanding women's preferences and concerns. *J Psychiatr Pract* 2013; **19**(6): 443-53.

25. Kothari A, de Laat J, Dulhunty JM, Bruxner G. Perceptions of pregnant women regarding antidepressant and anxiolytic medication use during pregnancy. *Australas Psychiatry* 2019; **27**(2): 117-20.

26. Molenaar NM, Brouwer ME, Duvekot JJ, et al. Antidepressants during pregnancy: Guideline adherence and current practice amongst Dutch gynaecologists and midwives. *Midwifery* 2018; **61**: 29-35.

27. Molenaar NM, Kamperman AM, Boyce P, Bergink V. Guidelines on treatment of perinatal depression with antidepressants: An international review. *Aust N Z J Psychiatry* 2018; **52**(4): 320-7.

28. Bakker MK, Kolling P, van den Berg PB, de Walle HE, de Jong van den Berg LT. Increase in use of selective serotonin reuptake inhibitors in pregnancy during the last decade, a population-based cohort study from the Netherlands. *Br J Clin Pharmacol* 2008; **65**(4): 600-6.

29. Molenaar NM, Lambregtse-van den Berg MP, Bonsel GJ. Dispensing patterns of selective serotonin reuptake inhibitors before, during and after pregnancy: a 16-year population-based cohort study from the Netherlands. *Arch Womens Ment Health* 2019.

30. Charlton RA, Jordan S, Pierini A, et al. Selective serotonin reuptake inhibitor prescribing before, during and after pregnancy: a population-based study in six European regions. *Bjog-Int J Obstet Gy* 2015; **122**(7): 1010-20.

31. Cooper WO, Willy ME, Pont SJ, Ray WA. Increasing use of antidepressants in pregnancy. *American Journal of Obstetrics and Gynecology* 2007; **196**(6): 544-5.

32. Jimenez-Solem E, Andersen JT, Petersen M, et al. Prevalence of Antidepressant Use during Pregnancy in Denmark, a Nation-Wide Cohort Study. *Plos One* 2013; **8**(4).

For peer review only

33. van Gelder MMHJ, Bos JHJ, Roeleveld N, de Jong-van den Berg LTW. Drugs associated with teratogenic mechanisms. Part I: dispensing rates among pregnant women in the Netherlands, 1998-2009. *Hum Reprod* 2014; **29**(1): 161-7.

34. Ververs T, Kaasenbrood H, Visser G, Schobben F, de Jong-van den Berg L, Egberts T. Prevalence and patterns of antidepressant drug use during pregnancy. *Eur J Clin Pharmacol* 2006; **62**(10): 863-70.

35. Ailes EC, Simeone RM, Dawson AL, Petersen EE, Gilboa SM. Using insurance claims data to identify and estimate critical periods in pregnancy: An application to antidepressants. *Birth Defects Res A Clin Mol Teratol* 2016; **106**(11): 927-34.

36. Andrade SE, Reichman ME, Mott K, et al. Use of selective serotonin reuptake inhibitors (SSRIs) in women delivering liveborn infants and other women of child-bearing age within the U.S. Food and Drug Administration's Mini-Sentinel program. *Arch Womens Ment Health* 2016; **19**(6): 969-77.

37. Taylor LG, Thelus Jean R, Gordon G, Fram D, Coster T. Development of a mother-child database for drug exposure and adverse event detection in the Military Health System. *Pharmacoepidemiol Drug Saf* 2015; **24**(5): 510-7.

38. Hanley GE, Mintzes B. Patterns of psychotropic medicine use in pregnancy in the United States from 2006 to 2011 among women with private insurance. *BMC Pregnancy Childbirth* 2014; **14**: 242.

39. van Ravesteyn LM, Lambregtse-van den Berg MP, Hoogendijk WJ, Kamperman AM. Interventions to treat mental disorders during pregnancy: A systematic review and multiple treatment meta-analysis. *PLoS One* 2017; **12**(3): e0173397.

40. Blume C, Garbazza C, Spitschan M. Effects of light on human circadian rhythms, sleep and mood. *Somnologie* 2019.

41. LeGates TA, Fernandez DC, Hattar S. Light as a central modulator of circadian rhythms, sleep and affect. *Nature Reviews Neuroscience* 2014; **15**(7): 443-54.

42. Prayag AS, Münch M, Aeschbach D, Chellappa SL, Gronfier C. Light modulation of human clocks, wake and sleep. *Clocks & Sleep* 2019; **1**: 193-208.

43. Germain A, Kupfer DJ. Circadian rhythm disturbances in depression. *Hum Psychopharmacol* 2008; **23**(7): 571-85.

For peer review only

For peer review only - http://bmjopen.bmj.com/site/about/guidelines.xhtml

44. Lam RW, Levitan RD. Pathophysiology of seasonal affective disorder: a review. *J Psychiatry Neurosci* 2000; **25**(5): 469-80.

45. Magnusson A, Boivin D. Seasonal affective disorder: an overview. *Chronobiol Int* 2003; **20**(2): 189-207.

46. Tuunainen A, Kripke DF, Endo T. Light therapy for non-seasonal depression. *Cochrane Database Syst Rev* 2004.

47. Golden RN, Gaynes BN, Ekstrom RD, et al. The efficacy of light therapy in the treatment of mood disorders: a review and meta-analysis of the evidence. *Am J Psychiatry* 2005; **162**(4): 656-62.

48. Al-Karawi D, Jubair L. Bright light therapy for nonseasonal depression: Meta-analysis of clinical trials. *J Affect Disord* 2016; **198**: 64-71.

49. Perera S, Eisen R, Bhatt M, et al. Light therapy for non-seasonal depression: systematic review and meta-analysis. *BJPsych Open* 2016; **2**(2): 116-26.

50. Martensson B, Pettersson A, Berglund L, Ekselius L. Bright white light therapy in depression: A critical review of the evidence. *J Affect Disord* 2015; **182**: 1-7.

51. Oren DA, Wisner KL, Spinelli M, et al. An open trial of morning light therapy for treatment of antepartum depression. *Am J Psychiatry* 2002; **159**(4): 666-9.

52. Epperson CN, Terman M, Terman JS, et al. Randomized clinical trial of bright light therapy for antepartum depression: preliminary findings. *J Clin Psychiatry* 2004; **65**(3): 421-5.

53. Wirz-Justice A, Bader A, Frisch U, et al. A randomized, double-blind, placebo-controlled study of light therapy for antepartum depression. *J Clin Psychiatry* 2011; **72**(7): 986-93.

54. Ioannidis JP. Why most published research findings are false. *PLoS Med* 2005; **2**(8): e124.

55. Bais B, Kamperman AM, van der Zwaag MD, et al. Bright light therapy in pregnant women with major depressive disorder: study protocol for a randomized, double-blind, controlled clinical trial. *BMC Psychiatry* 2016; **16**(1): 381.

56. First MB GM, Spitzer RL, Williams JBW. User's guide for the SCID-I. Structured clinical interview for DSM-IV TR axis I disorders (research version). New York: New York Psychiatric Institute; 2002.

For peer review only

57. Lieverse R, Van Someren EJ, Nielen MM, Uitdehaag BM, Smit JH, Hoogendijk WJ. Bright light treatment in elderly patients with nonseasonal major depressive disorder: a randomized placebo-controlled trial. *Arch Gen Psychiatry* 2011; **68**(1): 61-70.

58. Kreidler SM, Muller KE, Grunwald GK, et al. GLIMMPSE: Online Power Computation for Linear Models with and without a Baseline Covariate. *J Stat Softw* 2013; **54**(10).

59. Meesters Y, Dekker V, Schlangen LJ, Bos EH, Ruiter MJ. Low-intensity blue-enriched white light (750 lux) and standard bright light (10,000 lux) are equally effective in treating SAD. A randomized controlled study. *BMC Psychiatry* 2011; **11**: 17.

60. Project TAI. ALEA Randomisation Software (Version 2.2). Amsterdam: Netherlands Cancer Institute; 2006. p. http://tenalea.net/.

61. Bockting CL, Hollon SD, Jarrett RB, Kuyken W, Dobson K. A lifetime approach to major depressive disorder: The contributions of psychological interventions in preventing relapse and recurrence. *Clin Psychol Rev* 2015; **41**: 16-26.

62. Cox JL, Holden JM, Sagovsky R. Detection of postnatal depression. Development of the 10-item Edinburgh Postnatal Depression Scale. *Br J Psychiatry* 1987; **150**: 782-6.

63. Bergink V, Kooistra L, Lambregtse-van den Berg MP, et al. Validation of the Edinburgh Depression Scale during pregnancy. *J Psychosom Res* 2011; **70**(4): 385-9.

64. Roenneberg T, Wirz-Justice A, Merrow M. Life between clocks: daily temporal patterns of human chronotypes. *J Biol Rhythms* 2003; **18**(1): 80-90.

65. Merikanto I, Kronholm E, Peltonen M, Laatikainen T, Vartiainen E, Partonen T. Circadian preference links to depression in general adult population. *J Affect Disord* 2015; **188**: 143-8.

66. Merikanto I, Lahti T, Kronholm E, et al. Evening types are prone to depression. *Chronobiol Int* 2013; **30**(5): 719-25.

67. Schulz KF, Altman DG, Moher D, Group C. CONSORT 2010 Statement: updated guidelines for reporting parallel group randomised trials. *BMC Med* 2010; **8**: 18.

68. Friedman ES, Davis LL, Zisook S, et al. Baseline depression severity as a predictor of single and combination antidepressant treatment outcome: results from the CO-MED trial. *Eur Neuropsychopharmacol* 2012; **22**(3): 183-99.

For peer review only

69. Corral M, Wardrop AA, Zhang H, Grewal AK, Patton S. Morning light therapy for postpartum depression. *Arch Womens Ment Health* 2007; **10**(5): 221-4.

70. Rief W, Nestoriuc Y, Weiss S, Welzel E, Barsky AJ, Hofmann SG. Meta-analysis of the placebo response in antidepressant trials. *J Affect Disord* 2009; **118**(1-3): 1-8.

71. Posternak MA, Miller I. Untreated short-term course of major depression: a meta-analysis of outcomes from studies using wait-list control groups. *J Affect Disord* 2001; **66**(2-3): 139-46.

72. Yazici E, Kirkan TS, Aslan PA, Aydin N, Yazici AB. Untreated depression in the first trimester of pregnancy leads to postpartum depression: high rates from a natural follow-up study. *Neuropsychiatr Dis Treat* 2015; **11**: 405-11.

73. Khoury B, Lecomte T, Fortin G, et al. Mindfulness-based therapy: a comprehensive meta-analysis. *Clin Psychol Rev* 2013; **33**(6): 763-71.

74. Hofmann SG, Sawyer AT, Witt AA, Oh D. The effect of mindfulness-based therapy on anxiety and depression: A meta-analytic review. *J Consult Clin Psychol* 2010; **78**(2): 169-83.

75. Dimidjian S, Goodman SH, Felder JN, Gallop R, Brown AP, Beck A. An open trial of mindfulness-based cognitive therapy for the prevention of perinatal depressive relapse/recurrence. *Arch Womens Ment Health* 2015; **18**(1): 85-94.

76. Vieten C, Astin J. Effects of a mindfulness-based intervention during pregnancy on prenatal stress and mood: results of a pilot study. *Arch Womens Ment Health* 2008; **11**(1): 67-74.

77. Nagare R, Plitnick B, Figueiro MG. Effect of exposure duration and light spectra on nighttime melatonin suppression in adolescents and adults. *Light Res Technol* 2019; **51**(4): 530-43.

78. Zeitzer JM, Dijk DJ, Kronauer R, Brown E, Czeisler C. Sensitivity of the human circadian pacemaker to nocturnal light: melatonin phase resetting and suppression. *J Physiol* 2000; **526 Pt 3**: 695-702.

Table 1. Inclusion and exclusion criteria for the Bright Up Study.

**Inclusion criteria**	**Women**
	18-45 years of age
	12-32 weeks pregnant (as confirmed by ultrasound)

For peer review only

	Current DSM-5 diagnosis of depressive disorder (as assessed by the SCID*)
**Exclusion criteria**	Insufficient proficiency in Dutch or English
	Multiple pregnancy
	Current use of antidepressants shorter than 2 months
	Lifetime diagnosis of bipolar I or II disorder
	Any psychotic episode
	Current substance abuse
	Current primary anxiety disorder
	Recent history of suicide attempt
	Current shift-work
	Somatic and/or obstetric conditions that override study participation
	Previous treatment with BLT
	Eye condition (macular degeneration, eye diseases, recent eye surgery)

* SCID = Structured Clinical Interview for DSM disorders

Table 2. Overview of participant characteristics at inclusion.

	**BLT (n=33)**	**DRLT (n=34)**
**Age in years, mean (SD)**	31.9 (4.4)	31.9 (5.3)
**Gestational age in weeks, mean (SD)**	20.6 (6.2)	19.7 (6.3)
**Ethnicity**		
**Dutch**	27 (81.8%)	26 (76.5%)
**Other**	6 (19.2%)	8 (33.5%)
**Marital status**		
**Married or cohabiting**	33 (100%)	32 (94.1%)
**Committed relationship, not cohabiting**	0 (0%)	1 (2.9%)
**Single**	0 (0%)	1 (2.9%)

For peer review only

**Education**		
**Elementary or (pre-)vocational education**	11 (33.3%)	13 (38.2%)
**Higher professional education**	8 (24.2%)	11 (32.4%)
**(Pre-) academic education**	14 (42.4%)	10 (29.4%)
**Parity**		
**Nulliparous**	15 (45.5%)	20 (58.8%)
**Primiparous**	13 (39.4%)	9 (26.5%)
**Multiparous**	5 (15.2%)	5 (14.7%)
**BMI in kg/m² or st/ft², mean (SD)**	25.5 (4.5)	26.3 (5.4)
**Planned pregnancy**	22 (66.7%)	22 (64.7%)
**Antidepressant medication**	3 (9.1%)	5 (14.7%)
**Sleep medication**	3 (9.1%)	2 (5.9%)
**Psychotherapy**	14 (48.5%)	16 (47.1%)
**Comorbidities**		
**0**	17 (51.5%)	13 (38.2%)
**1**	9 (27.3%)	13 (38.2%)
**>1**	7 (21.2%)	8 (23.5%)
**Duration of depression in weeks, mean (SD)**	24.6 (16.9)	45.1 (121.9)
**Depressive episodes in past**		
**0**	12 (36.4%)	11 (32.4%)
**1**	9 (27.2%)	14 (41.2%)
**>1**	12 (36.4%)	9 (26.5%)
**Chronotype**		
**Early (extremely, moderately and slightly)**	20 (80%)	25 (92.6%)
**Normal**	1 (4%)	1 (3.7%)
**Late (extremely, moderately and slightly)**	4 (16%)	1 (3.7%)

695 BLT = bright light therapy; DRLT = dim red light therapy

696

For peer review only

Table 3. Effects of allocation on the course of depressive symptoms through the intervention period and follow-up (until two months postpartum): crude analysis

	β (95% CI) of intervention*	β (95% CI) of follow-up**
**SIGH-SAD**	-0.68 (-1.84, 0.49)	-0.16 (-0.82, 0.51)
**HAM-D**	-0.18 (-0.74, 0.37)	0.04 (-0.29, 0.37)
**EPDS**	0.01 (-0.51, 0.53)	-0.05 (-0.35, 0.24)

* From start of study until end of treatment; ** From start of study until follow-up 2 months postpartum

Figure legends

Figure 1. Flow-chart of the Bright Up study.

Figure 2. Estimated marginal means of depression scores in women with antepartum depression until two months postpartum. Shown are SIGH-SAD, HAM-D and EPDS scores. Black lines represent treatment with BLT, gray lines with DRLT. Bars represent standard error of the mean.

BLT = bright light therapy; DRLT = dim red light therapy; SIGH- SAD = Structured Interview Guide for the Hamilton Depression Scale – Seasonal Affective Disorder version; HAM-D = Hamilton Rating Scale for Depression; EPDS = Edinburgh Postnatal Depression Scale; T0 = baseline, before treatment; T0+1, T0+2 ... T0+5 = weeks during intervention period; T1 = end of treatment; T2 = 3 weeks after end of treatment; T3 = 10 weeks after end of treatment; P1 = 2 months postpartum

For peer review only

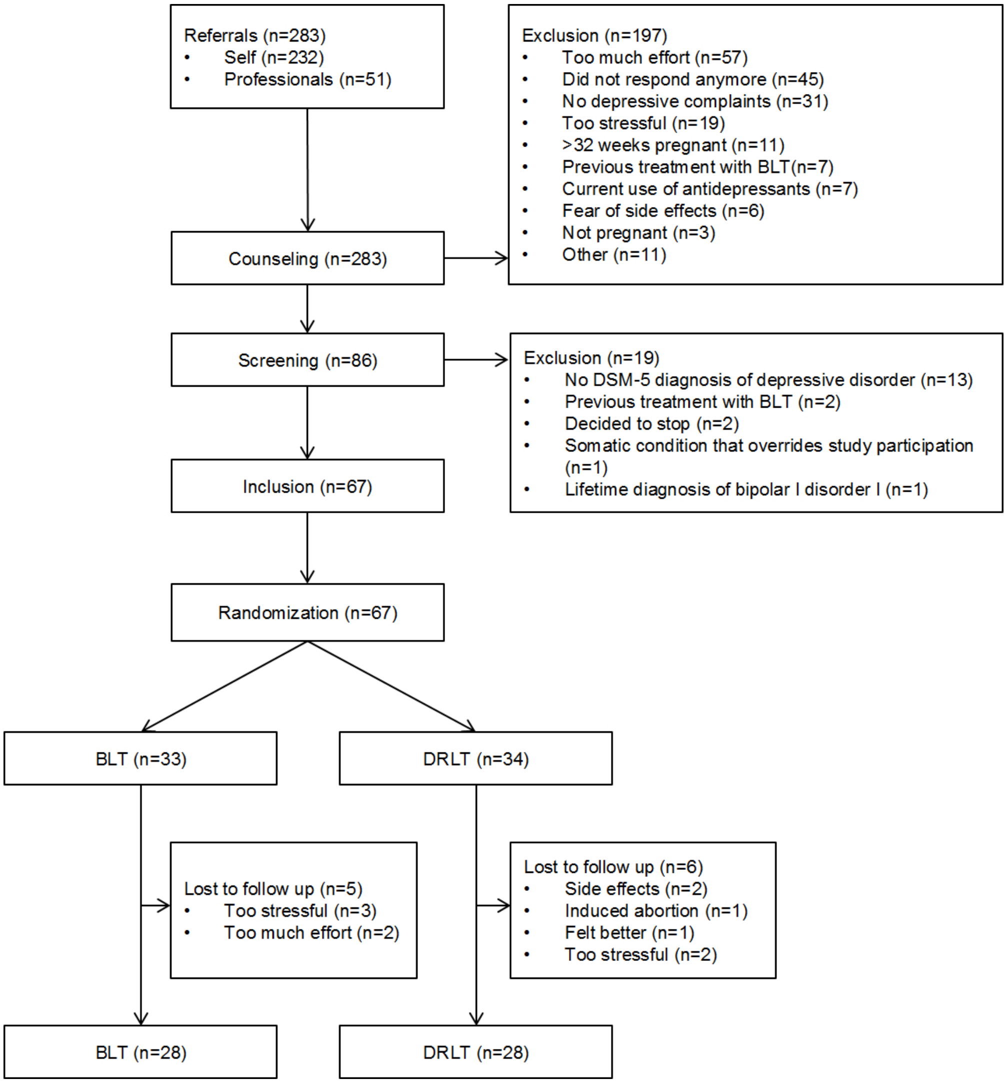

Figure 1. Flow-chart of the Bright Up study.

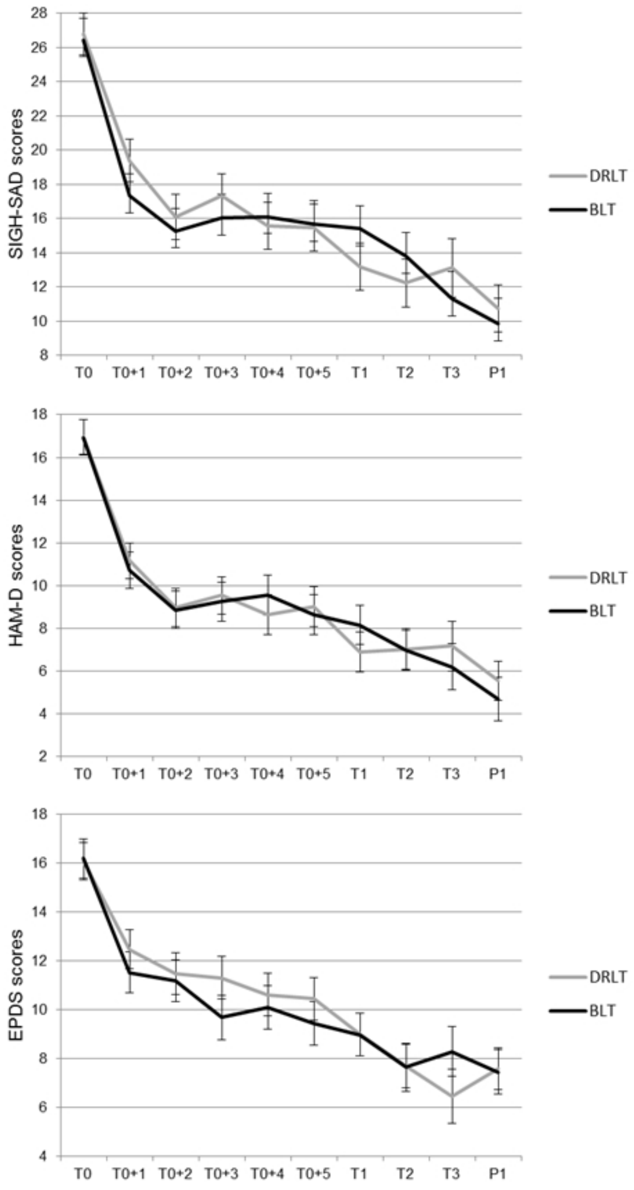

Figure 2. Estimated marginal means of depression scores in women with antepartum depression until two months postpartum. Shown are SIGH-SAD, HAM-D and EPDS scores. Black lines represent treatment with BLT, gray lines with DRLT. Bars represent standard error of the mean.
BLT = bright light therapy; DRLT = dim red light therapy; SIGH- SAD = Structured Interview Guide for the Hamilton Depression Scale – Seasonal Affective Disorder version; HAM-D = Hamilton Rating Scale for Depression; EPDS = Edinburgh Postnatal Depression Scale; T0 = baseline, before treatment; T0+1, T0+2 ... T0+5 = weeks during intervention period; T1 = end of treatment; T2 = 3 weeks after end of treatment; T3 = 10 weeks after end of treatment; P1 = 2 months postpartum

Supplementary Table 1. Photobiological characterizations of light therapy in both treatment arms.

	BLT	DRLT
Cyanopic irradiance (μW · cm^{-2})	578.7	2.24
Melanopic irradiance (μW · cm^{-2})	891	5.53
Chloropic irradiance (μW · cm^{-2})	1032.3	7.23
Erythropic irradiance (μW · cm^{-2})	1212.3	11.37
Rhodopic irradiance (μW · cm^{-2})	16.61	16.61

BLT = bright light therapy; DRLT = dim red light therapy

For peer review only

Supplementary Table 2. Observed median SIGH-SAD, HAM-D and EPDS scores with ranges and number of participants over the course of the study for both treatment arms.

Measure	T0	T0+1	T0+2	T0+3	T0+4	T0+5	T1	T2	T3	P1
**SIGH-SAD**										
**BLT (mdn, range, N)**	27 (14-44; 33)	16.5 (1-33; 30)	16 (2-43; 29)	15 (0-41; 25)	18 (0-32; 25)	17.5 (1-37; 24)	15.5 (0-29; 26)	13 (1-26; 25)	11 (0-29; 17)	8 (1-23; 20)
**DRLT (mdn, range, N)**	26.5 (13-42; 34)	19 (8-33; 31)	17 (2-35; 27)	18 (4-30; 29)	15 (3-28; 24)	16 (2-31; 25)	13 (2-34; 25)	11.5 (1-26; 24)	9.5 (1-31; 14)	8 (0-28; 25)
**HAM-D**										
**BLT (mdn, range, N)**	16 (7-29; 33)	9 (0-25; 30)	9 (1-30; 29)	8 (0-28; 25)	10 (0-22; 25)	10 (0-21; 24)	7.5 (0-20; 26)	8 (0-17; 25)	5 (0-16; 17)	3 (0-11; 20)
**DRLT (mdn, range, N)**	18 (4-29; 34)	10 (3-20; 31)	9 (1-22; 27)	9 (2-20; 29)	8 (0-18; 24)	8 (1-20; 25)	6 (1-18; 25)	4.5 (0-20; 24)	4 (0-15; 14)	4 (0-19; 25)
**EPDS**										
**BLT (mdn, range, N)**	16 (7-25; 31)	11 (3-23; 26)	11 (0-23; 26)	10 (0-19; 21)	8 (0-25; 23)	7 (0-18; 23)	9.5 (1-18; 26)	8.5 (0-15; 18)	8.5 (1-24; 16)	7 (0-13; 22)
**DRLT (mdn, range, N)**	16 (3-25; 34)	12 (6-19; 28)	12 (3-20; 25)	11.5 (3-21; 24)	10 (1-18; 24)	10 (2-19; 23)	6.5 (1-22; 24)	6 (0-21; 23)	4 (1-10; 12)	7 (0-18; 26)

BLT = bright light therapy; DRLT = dim red light therapy; SIGH- SAD = Structured Interview Guide for the Hamilton Depression Scale – Seasonal Affective Disorder version; HAM-D = Hamilton Rating Scale for Depression; EPDS = Edinburgh Postnatal Depression Scale; T0 = baseline, before treatment; T0+1, T0+2 ... T0+5 = weeks during intervention period; T1 = end of treatment; T2 = 3 weeks after end of treatment; T3 = 10 weeks after end of treatment; P1 = 2 months postpartum; mdn = median

For peer review only

Supplementary Table 3. Effects of allocation on the course of depressive symptoms through the intervention period and follow-up (until two months postpartum): sensitivity analyses.

	β (95% CI) of intervention*	β (95% CI) of follow-up**
**Adjusted analysis[a]**		
**SIGH-SAD**	-0.24 (-1.68, 1.20)	-0.24 (-1.68, 1.20)
**HAM-D**	0.13 (-0.49, 0.75)	0.13 (-0.49, 0.75)
**EPDS**	0.25 (-0.38, 0.89)	0.25 (-0.38, 0.89)
**Data imputation[b]**		
**SIGH-SAD**	-0.45 (-1.44, 0.53)	-0.08 (-0.63, 0.46)
**HAM-D**	-0.09 (-0.63, 0.44)	0.06 (-0.25, 0.37)
**EPDS**	0.19 (-0.30, 0.68)	0.04 (-0.24, 0.32)
**Post-hoc analysis: high treatment compliance[c]**		
**SIGH-SAD**	-0.40 (-1.36, 0.55)	-0.32 (-0.88, 0.24)
**HAM-D**	-0.12 (-0.79, 0.54)	-0.06 (-0.43, 0.31)
**EPDS**	0.03 (-0.58, 0.65)	-0.05 (-0.40, 0.30)
**Post-hoc analysis: high symptom severity[d]**		
**SIGH-SAD**	-0.84 (-2.33, 0.65)	-0.20 (-1.14, 0.75)
**HAM-D**	-0.16 (-1.12, 0.87)	0.13 (-0.48, 0.73)
**EPDS**	-0.05 (-0.92, 0.82)	0.20 (-0.33, 0.74)

For peer review only

* From start of study until end of treatment; ** From start of study until follow-up 2 months postpartum; [a] Propensity score composed of psychiatric history, ethnicity, level of education, an unplanned pregnancy, maternal age, parity, gestational age, duration of actual depression and other psychiatric or psychotherapeutic treatment interventions; [b] Last observation carried forward; [c] <7 missed treatments; [d] Based on median split baseline SIGH-SAD scores

For peer review only

CONSORT 2010 checklist of information to include when reporting a randomised trial*

Section/Topic	Item No	Checklist item	Reported on page No
**Title and abstract**			
	1a	Identification as a randomised trial in the title	p. 1
	1b	Structured summary of trial design, methods, results, and conclusions (for specific guidance see CONSORT for abstracts)	p. 2
**Introduction**			
Background and objectives	2a	Scientific background and explanation of rationale	p. 4-5
	2b	Specific objectives or hypotheses	p. 5
**Methods**			
Trial design	3a	Description of trial design (such as parallel, factorial) including allocation ratio	p. 5
	3b	Important changes to methods after trial commencement (such as eligibility criteria), with reasons	p. 6
Participants	4a	Eligibility criteria for participants	p. 6; Table 1
	4b	Settings and locations where the data were collected	p. 6
Interventions	5	The interventions for each group with sufficient details to allow replication, including how and when they were actually administered	p. 7-8
Outcomes	6a	Completely defined pre-specified primary and secondary outcome measures, including how and when they were assessed	p. 8-11
	6b	Any changes to trial outcomes after the trial commenced, with reasons	n.a.
Sample size	7a	How sample size was determined	p. 6
	7b	When applicable, explanation of any interim analyses and stopping guidelines	n.a.
Randomisation:			
Sequence generation	8a	Method used to generate the random allocation sequence	p. 9
	8b	Type of randomisation; details of any restriction (such as blocking and block size)	p. 9
Allocation concealment mechanism	9	Mechanism used to implement the random allocation sequence (such as sequentially numbered containers), describing any steps taken to conceal the sequence until interventions were assigned	p. 7-9
Implementation	10	Who generated the random allocation sequence, who enrolled participants, and who assigned participants to interventions	p. 9
Blinding	11a	If done, who was blinded after assignment to interventions (for example, participants, care providers, those	p. 7

For peer review only

		assessing outcomes) and how	
	11b	If relevant, description of the similarity of interventions	p. 7-8
Statistical methods	12a	Statistical methods used to compare groups for primary and secondary outcomes	p. 11-12
	12b	Methods for additional analyses, such as subgroup analyses and adjusted analyses	p. 11-12
**Results**			
Participant flow (a diagram is strongly recommended)	13a	For each group, the numbers of participants who were randomly assigned, received intended treatment, and were analysed for the primary outcome	p. 12, Fig. 1, Supp. Table 2
	13b	For each group, losses and exclusions after randomisation, together with reasons	Fig. 1
Recruitment	14a	Dates defining the periods of recruitment and follow-up	p. 6
	14b	Why the trial ended or was stopped	p. 6
Baseline data	15	A table showing baseline demographic and clinical characteristics for each group	Table 2
Numbers analysed	16	For each group, number of participants (denominator) included in each analysis and whether the analysis was by original assigned groups	Sup. Table 2
Outcomes and estimation	17a	For each primary and secondary outcome, results for each group, and the estimated effect size and its precision (such as 95% confidence interval)	Sup. Table 2, Fig. 2, p.13-15, Table 3
	17b	For binary outcomes, presentation of both absolute and relative effect sizes is recommended	p. 14
Ancillary analyses	18	Results of any other analyses performed, including subgroup analyses and adjusted analyses, distinguishing pre-specified from exploratory	p. 14, Sup. Table 3
Harms	19	All important harms or unintended effects in each group (for specific guidance see CONSORT for harms)	p. 14
**Discussion**			
Limitations	20	Trial limitations, addressing sources of potential bias, imprecision, and, if relevant, multiplicity of analyses	p. 17-18
Generalisability	21	Generalisability (external validity, applicability) of the trial findings	p. 15-17
Interpretation	22	Interpretation consistent with results, balancing benefits and harms, and considering other relevant evidence	p. 15-17
**Other information**			
Registration	23	Registration number and name of trial registry	p. 2, 6
Protocol	24	Where the full trial protocol can be accessed, if available	p. 6
Funding	25	Sources of funding and other support (such as supply of drugs), role of funders	p. 19

*We strongly recommend reading this statement in conjunction with the CONSORT 2010 Explanation and Elaboration for important clarifications on all the items. If relevant, we also recommend reading CONSORT extensions for cluster randomised trials, non-inferiority and equivalence trials, non-pharmacological treatments, herbal interventions, and pragmatic trials. Additional extensions are forthcoming: for those and for up to date references relevant to this checklist, see www.consort-statement.org.

For peer review only - http://bmjopen.bmj.com/site/about/guidelines.xhtml

BMJ Open

Effects of bright light therapy for depression during pregnancy: a randomized, double-blind controlled trial

Journal:	*BMJ Open*
Manuscript ID	bmjopen-2020-038030.R2
Article Type:	Original research
Date Submitted by the Author:	30-Sep-2020
Complete List of Authors:	Bais, Babette; Erasmus Medical Center, Psychiatry<br>Kamperman, Astrid M.; Erasmus MC, Psychiatry<br>Bijma, Hilmar; Erasmus Medical Center, Obstetrics and Gynaecology<br>Hoogendijk, Witte; Erasmus Medical Center, Psychiatry<br>Souman, Jan; Signify NV, Lighting Applications<br>Knijff, Esther; Erasmus Medical Center, Psychiatry<br>Lambregtse-van den Berg, Mijke; Erasmus Medical Center, Child and Adolescent Psychiatry/Psychology; Erasmus Medical Center, Psychiatry
**Primary Subject Heading**:	Mental health
Secondary Subject Heading:	Obstetrics and gynaecology
Keywords:	Depression & mood disorders < PSYCHIATRY, OBSTETRICS, PSYCHIATRY

SCHOLARONE™
Manuscripts

I, the Submitting Author has the right to grant and does grant on behalf of all authors of the Work (as defined in the below author licence), an exclusive licence and/or a non-exclusive licence for contributions from authors who are: i) UK Crown employees; ii) where BMJ has agreed a CC-BY licence shall apply, and/or iii) in accordance with the terms applicable for US Federal Government officers or employees acting as part of their official duties; on a worldwide, perpetual, irrevocable, royalty-free basis to BMJ Publishing Group Ltd ("BMJ") its licensees and where the relevant Journal is co-owned by BMJ to the co-owners of the Journal, to publish the Work in this journal and any other BMJ products and to exploit all rights, as set out in our licence.

The Submitting Author accepts and understands that any supply made under these terms is made by BMJ to the Submitting Author unless you are acting as an employee on behalf of your employer or a postgraduate student of an affiliated institution which is paying any applicable article publishing charge ("APC") for Open Access articles. Where the Submitting Author wishes to make the Work available on an Open Access basis (and intends to pay the relevant APC), the terms of reuse of such Open Access shall be governed by a Creative Commons licence – details of these licences and which Creative Commons licence will apply to this Work are set out in our licence referred to above.

Other than as permitted in any relevant BMJ Author's Self Archiving Policies, I confirm this Work has not been accepted for publication elsewhere, is not being considered for publication elsewhere and does not duplicate material already published. I confirm all authors consent to publication of this Work and authorise the granting of this licence.

For peer review only

Effects of bright light therapy for depression during pregnancy: a randomized, double-blind controlled trial

Babette Bais, MSc[1]*, Astrid M Kamperman, PhD[2], Hilmar H Bijma, MD, PhD[3], Witte JG Hoogendijk, MD, PhD[4], Jan L Souman, PhD[5], Esther Knijff, MD, PhD[6], Mijke P Lambregtse-van den Berg, MD, PhD[7]

[1] Department of Psychiatry, Erasmus University Medical Centre Rotterdam, Rotterdam, The Netherlands

[2] Epidemiological and Social Psychiatric Research Institute, Department of Psychiatry, Erasmus University Medical Centre Rotterdam, Rotterdam, The Netherlands

[3] Department of Obstetrics and Gynaecology, Erasmus University Medical Centre Rotterdam, Rotterdam, The Netherlands

[4] Department of Psychiatry, Erasmus University Medical Centre Rotterdam, Rotterdam, The Netherlands

[5] Department Lighting Applications, Signify Research, Eindhoven, The Netherlands;

[6] Department of Psychiatry, Erasmus University Medical Centre Rotterdam, Rotterdam, The Netherlands

[7] Department of Child and Adolescent Psychiatry/Psychology, department of Psychiatry, Erasmus University Medical Centre Rotterdam, Rotterdam, The Netherlands

*Corresponding author: P.O. Box 2040, 3000CA Rotterdam, The Netherlands; b.bais@erasmusmc.nl

For peer review only

Abstract

Objectives Approximately 11-13% of pregnant women suffer from depression. Bright light therapy (BLT) is a promising treatment, combining direct availability, sufficient efficacy, low costs, and high safety for both mother and child. Here, we examined the effects of BLT on depression during pregnancy.

Design Randomized, double-blind controlled trial.

Setting Primary and secondary care in The Netherlands, from November 2016 to March 2019.

Participants 67 pregnant women (12-32 weeks gestational age) with a DSM-5 diagnosis of depressive disorder.

Interventions Participants were randomly allocated to treatment with either BLT (9,000 lux, 5,000 K) or dim red light therapy (DRLT, 100 lux, 2,700 K), which is considered placebo. For six weeks, both groups were treated daily at home for 30 minutes upon awakening. Follow-up took place weekly during the intervention, after six weeks of therapy, three and ten weeks after treatment, and two months postpartum.

Primary and secondary outcome measures Depressive symptoms were measured primarily with the Structured Interview Guide for the Hamilton Depression Scale – Seasonal Affective Disorder. Secondary measures were the Hamilton Rating Scale for Depression and the Edinburgh Postnatal Depression Scale. Changes in rating scale scores of these questionnaires over time were analysed using generalized linear mixed models.

Results Median depression scores decreased by 40.6-53.1% in the BLT group and by 50.9-66.7% in the DRLT group. We found no statistically significant difference in symptom change scores between BLT and DRLT. Sensitivity and post-hoc analyses did not change our findings.

Conclusions Depressive symptoms of pregnant women with depression improved in both treatment arms. More research is necessary to determine whether these responses represent true treatment effects, non-specific treatment responses, placebo effects, or a combination hereof.

Trial Registration Bright Up, NTR5476, http://www.trialregister.nl

Strengths and limitations of this study

For peer review only

- We conducted various follow up measurements, including postpartum, to study the effects of withdrawal of treatment and to study whether treatment during pregnancy would protect against postpartum depression.
- The setting of treatment was within a real world setting.
- A strength of this study was the comprehensive assessment of side effects, as well as acceptability and satisfaction of treatment.
- An unforeseen lack of resources prevented us from including 150 participants, as we aimed to do according to our sample size calculation.
- Depressive symptoms during the study are assessed by questionnaires, rather than diagnostic criteria.

For peer review only

Introduction

Antepartum depression is a common and high impact disease, with approximately 11-13% of pregnant women suffering from depression [1]. Antepartum depression is not only seen in autumn and winter, but is a year-round phenomenon, with certain subgroups even showing more symptoms in summer [2]. Many risk factors for antepartum depression have been identified [3,4]. Possible causes for antepartum depression may include alterations in endocrine systems, such as the hypothalamus-pituitary-adrenal axis [5], and inflammation [6,7]. Women who suffer from antepartum depression are more likely to suffer from postpartum depression as well [8]. Children who are exposed to maternal depression during pregnancy have a higher risk of adverse birth outcomes, such as prematurity and being small for gestational age [9,10]. Additionally, children of mothers with antepartum depression show more often cognitive, emotional, and behavioral problems in childhood, adolescence, and adulthood [11,12] and they have a higher risk of suffering from depression later in life [13]. During pregnancy, fetal programming of the hypothalamus-pituitary-adrenal gland (HPA) axis takes place, which can be affected by maternal depression during pregnancy and may have long-lasting effects on stress response [14]. Possible mechanisms are 1) maternal cortisol crossing the placenta and thus increasing fetal cortisol levels, 2) placental secretion of corticotropin-releasing factor, which stimulates both maternal and fetal cortisol, and 3) reduced blood flow to the fetus, causing fetal growth restriction [9,15-18]. In addition, epigenetic programming takes place within the antepartum period, which influences not only the health of the (unborn) infant, but also that of following generations [19]. Therefore, early detection and treatment of antepartum depression is highly important for both mother and infant.

In non-pregnant women, guidelines propose psychotherapy, antidepressant medication, or a combination of both as treatment. However, psychotherapy might not be readily available and the safety of maternal use of antidepressants, which cross the placenta, still remains to be established. The use of antidepressants is controversial, because of potential teratogenicity [20,21]. For example, increased risks have been found for persistent pulmonary hypertension of the neonate [22] and cardiovascular malformations [23]. Furthermore, pregnant women express a strong preference for non-pharmacologic treatment because of the possible harm for their unborn child [24,25]. Moreover, current adherence to national guidelines by midwives and gynaecologists is low [26] and international guidelines on the

For peer review only

pharmacological treatment of antepartum depression are not consistent [27], which might result in unwanted variation in practice. Despite this, antidepressant use during pregnancy is increasing, not only in the Netherlands [28,29], but in other European countries and the United States as well [30-32]. In the Netherlands, approximately 2-3% of pregnant women use antidepressants [29,33,34]. In the United States, this prevalence is approximately 6-7% [35-37], but could even be as high as 15% in some states [38]. Therefore, it is urgent and clinically relevant to investigate alternative approaches to treat antepartum depression, such as bright light therapy (BLT) [39].

Light synchronizes the suprachiasmatic nucleus (SCN), or the ‘biological clock’, with the environmental day-night rhythm [40]. Light hits the retina and intrinsically photosensitive retinal ganglion cells (ipRGCs) in the retina project, via the retino-hypthalamic tract to the SCN and thus influences circadian rhythm [40-42], which may indirectly benefit depressive symptoms [43]. However, not only do ipRGCs project to the SCN, but also directly to brain regions important in the regulation of mood, such as the medial amygdala and the lateral habenula [40-42].

Although BLT is the first-choice treatment for seasonal affective disorder, a condition of reoccurring depressions during fall and winter, with remissions in spring and summer [44,45], the effects of BLT have been shown both in seasonal affective disorder and in non-seasonal depression, which is not only shown by a Cochrane review [46], but also by more recent systematic reviews and meta-analyses [47-50]. An open trial of BLT in pregnant women showed improvement of mean depression ratings by 49% [51]. Two small randomized controlled trials showed significant improvement of depression among pregnant women exposed to BLT compared to placebo [52,53]. Although these results seem promising, the sample sizes of these studies were small, making them at risk for chance-findings [54].

In this study, we compared the effectiveness of BLT compared to placebo light among pregnant women with a depressive disorder in a larger randomized clinical trial. Moreover, we followed women until the postpartum period, to study whether treatment with light therapy during pregnancy might protect against postpartum depression. We hypothesized that daily treatment with six weeks of morning BLT will improve depressive symptoms during pregnancy.

Material and Methods

For peer review only

Design

This study was a randomized, double-blind, placebo-controlled clinical trial (Bright Up, NTR5476, http://www.trialregister.nl). A detailed protocol can be found elsewhere [55]. In short, the aim of the Bright Up study was to evaluate the effectiveness of BLT for pregnant women with a depressive disorder, compared to placebo light.

Participants

Eligible participants were pregnant women (12-32 weeks of gestational age, confirmed by ultrasound) diagnosed with a depressive disorder, confirmed by a Structured Clinical Interview for DSM disorders (SCID) by one trained assessor [56]. The specific inclusion and exclusion criteria are listed in Table 1. In the earlier published study protocol [55], we aimed to include women who were 12-18 weeks pregnant. For pragmatic reasons, in particular the fact that a substantial number of women were referred after 18 weeks of pregnancy, we later decided to widen our inclusion criteria to 12-32 weeks pregnancy. In the Netherlands, maternity care for low-risk pregnancies is provided by midwives (primary care). High-risk pregnancies are cared for by gynaecologists in a general hospital (secondary care) or fetal-maternal medicine unit (tertiary care).

In this study, women were recruited not only via health care professionals, such as general practitioners, midwifes, gynaecologists, psychiatrists, and psychologists, but also via (social) media. A complete flow-chart of the recruitment can be found in Figure 1.

Initially, we calculated the number of women to be included, based on the results and research methodology of previous studies [51,52,57]. We expected a true treatment effect in the range of a 10-15% symptom reduction over the full course of treatment (6 weekly assessments), reflecting a small to medium effect size. A sample size calculation was performed using GLIMMPSE 2.1.5. software [58], with the following parameters: alpha 0.05; beta 0.80; 6 time assessments (continuous, equally spaced); primary test: time*treatment interaction; SIGH-SAD scores assumed at baseline: M: 28.0 and SD: 7.0, with a linear decrease in symptom scores up to a mean score of 24.0 in the BLT condition. No symptom change was assumed for the DRLT condition; Hotelling-Lawley Trace correction; base correlation 0.4; decay rate 0.05; no additional scaling factors included.

For peer review only

To demonstrate this a total sample size of 126 participants, 63 per arm was needed. To account for loss to follow up during and after treatment, we aimed at including 150 women. Inclusion took place in The Netherlands and started on 9 November 2016 and lasted until 15 March 2019. By then, 67 women were included. However, due to limiting resources, we decided to stop the inclusion.

Patient and Public Involvement

No patients involved.

Ethics

All procedures performed involving human participants were in accordance with the ethical standards of the institutional and/or national research committee and with the 1964 Helsinki declaration and its later amendments or comparable ethical standards. Written informed consent was obtained from all participants. The study protocol and later amendments were approved by the medical ethical committee of the Erasmus University Medical Centre, Rotterdam, The Netherlands (registration number MEC-2015-731).

Blinding

Participants were blinded to allocation. Participants were informed that the study aimed to investigate the efficacy of different light colours. They were not informed that one treatment arm was considered placebo treatment. This was in accordance with approval of the medical ethical committee.

Outcome assessors were blinded to the allocation of the participants. Participants were asked not to share any details regarding their treatment towards the assessors. When blinding was broken, the assessor was replaced. The researcher performing the primary statistical analyses (AK) was blinded to the allocation. The field researcher (BB) was not blinded to the allocation for practical reasons. This researcher made sure lamps of the correct allocation were delivered to the participants. Also, this researcher asked participants about any side effects, keeping the independent assessors blinded to any adverse effects that might break the blinding, e.g. strained eyes, and answered any questions from the participants regarding their lamps.

For peer review only

At baseline, we asked about any expectations concerning the treatment with regards to their depressive symptoms. Women could choose whether they expected a negative effect, a small negative effect, no effect, a small positive effect or a positive effect. After the intervention period, the participants were asked whether they were aware of their allocation.

Light therapy

Light treatment consisted of either active BLT (9,000 lux, color temperature 5,000 K) or dim red light therapy (DRLT, 100 lux, color temperature 2,700 K). The photobiological characterizations of these treatments are shown in Supplementary Table 1. The original lamps were adjusted in the factory where these are produced (EnergyUp HF3419/01, Philips, Eindhoven, The Netherlands). To ensure that participants are exposed to the same light intensity, the output of the lamps was fixed. For the control condition, the standard LED's in the lamp were replaced by LED's with a lower intensity and a different color temperature. The lamps in the control condition were positioned at the same distance from the participant as in the experimental condition.

The active light therapy was shown to be effective in other studies [52,53,57,59]. DRLT can be considered to be biologically inactive and thus as placebo treatment [46]. In line with two previous RCT's among pregnant women, we chose six weeks of daily light exposure [52,53].

The lamps were delivered at the participants' home by one researcher (BB) who was not blinded to the allocation of the participants. This researcher did not share anything about the allocation with the participants. After delivery of the lamps and instructions, participants commenced their daily treatment with light for 30 minutes within 30 minutes of habitual wake up time for a six weeks period. This took place at the participants' home. Participants sat in front of two lamps with a distance of approximately 40 cm (15.8 inches). They received a plastic ruler of this length to ensure of the correct distance. The light boxes were placed in a custom-made scaffolding, so that the height of the light boxes could be adjusted per person and glare was avoided. Apart from the light treatment, participants in both treatment arms received treatment as usual: women were free to visit their general practitioner, obstetric care provider, or mental health care worker and start additional treatment, whenever they felt a need for this.

During the intervention period, self-reported compliance with the light treatment was checked weekly.

For peer review only

Method

A baseline interview was conducted by telephone by one researcher (BB). The baseline interview collected sociodemographic information (age, ethnicity, educational level, marital status, body mass index (BMI)), obstetric information (gestational age, whether the pregnancy was planned, parity), psychiatric information (substance use (smoking, alcohol, drugs), present and past medication use, present depressive symptoms, psychiatric history), and information on somatic conditions. Also, participants were screened with the SCID for depressive disorder and various potential co-morbidities, such as generalized anxiety disorder and panic disorder. Previous depressive episodes were also assessed with the SCID. The general practitioner was contacted to verify present medication use and whether the participant met any exclusion criteria.

After baseline measurements and receiving written informed consent, the participants were randomly allocated to either receive BLT or DRLT in a 1:1 ratio. Randomization was done with the web-based computer-generated schedule ALEA (software for randomization in clinical trials, version 2.2) using random block sizes of 2-6 [60] by an independent researcher. Stratification factors were the use of any current antidepressant medication and the number of previous depressive episodes. The latter was dichotomized to three or less versus four or more [61].

Follow up took place at the following time points: weekly during the intervention period (T0+1, T0+2, etc.), after 6 weeks of treatment (T1), 3 weeks after end of treatment (T2), 10 weeks after end of treatment (T3), 2 months postpartum (P1), 6 months postpartum (P2), 18 months postpartum (P3).

At these time points, questionnaires were assessed and body material was collected. We collected urine, hair, and saliva from the participants, as can be found in our earlier published protocol [55].

This paper reports the short term effectiveness, i.e. up to two months postpartum.

Primary and secondary outcome measures

The primary outcome measure was the average change in depressive symptoms between the two groups, as measured by the Structured Interview Guide for the Hamilton Depression Scale – Seasonal Affective Disorder version (SIGH-SAD). Secondary outcome measures were these changes as measured

by the 17-item Hamilton Rating Scale for Depression (HAM-D) and the Edinburgh Depression Scale (EPDS).

In the earlier published protocol [55], we were primarily interested in the effects of light therapy on depressive symptoms. Secondarily, we were interested in the effects on various other outcomes, such as maternal hormonal levels, maternal sleep quality and infant outcomes. Depressive symptoms were measured by two questionnaires: the SIGH-SAD and the EPDS, with the original 17-item HAM-D being part of the SIGH-SAD, which consists of 21 HAM-D items and 8 atypical items. Therefore, in the original protocol [55], we mentioned these two questionnaires together as the primary outcome, as opposed to the other outcomes (maternal hormonal levels and others). However, it is not technically possible to have more than one primary outcome. Our power calculation was based on the SIGH-SAD, which makes this our true primary outcome. The HAM-D and the EPDS are the secondary outcomes for this manuscript. In the current manuscript, we only report our findings regarding the depressive symptoms. We will report the other outcomes elsewhere. Second, in the trial register, we mention the HAM-D and EPDS as primary outcome, which has been a mistake. The mix-up results from the fact that the SIGH-SAD is in fact the original 17-item HAM-D with an additional 4 HAM-D and 8 atypical depressive items [62], and the inclusion of women with antepartum depressive mood disorder instead of seasonal affective disorder.

The SIGH-SAD is a 29-item structured interview, consisting of 21 HAM-D items and 8 atypical items. We used the entire SIGH-SAD questionnaire as primary measure, since this is the current benchmark for assessment of depression severity in light therapy trials [63]. We chose the original 17-item HAM-D questionnaire as a secondary measure, since it is more commonly used in clinical practice and research. Blinded assessors conducted the SIGH-SAD interviews (including HAM-D questions) by telephone weekly in the intervention period and at follow up.

The EPDS is a structured 10-item questionnaire and was used as a self-report measure of depression during pregnancy and postpartum [64]. Items are scored with a value 0-3, resulting in a sum score of 0-30 [64]. The EPDS was developed for the detection of postpartum depression, but has been validated for screening depression during pregnancy as well [65]. The EPDS was assessed weekly in the intervention period and at follow up. Participants received a link by e-mail to fill out the questionnaire.

For peer review only

Side effects, acceptability and satisfaction

During the intervention period, participants were asked weekly about any possible side effects. Acceptability was assessed by asking participants about their subjective treatment experiences after the intervention period. Women could choose whether they experienced a negative effect, a small negative effect, no effect, a small positive effect, or a positive effect. Women were asked how easy or difficult they could implement the therapy in their daily schedule and how easy or difficult the lamp was in use: very difficult, difficult, neutral, easy, or very easy. Women could answer whether they found the light therapy very unpleasant, unpleasant, neutral, pleasant, or very pleasant. Women were asked whether they would like to use the light therapy outside of the study (yes/no). Finally, women were asked how likely they would recommend light therapy to others on a scale of 1 to 10.

Baseline characteristics

The baseline interview collected information on various potential confounders, such as sociodemographic, obstetric, and psychiatric information, and information on somatic conditions (see Method for further specifications).

The participant's chronotype was assessed at inclusion with the Munich Chronotype Questionnaire (MCTQ), a structured 19-item self-report questionnaire [66], since evening types are more prone to depression compared to morning types [67,68]. The participant can be classified into one of seven chronotypes: extremely, moderately or slightly early, normal or slightly, moderately or extremely late. Sum scores range from 16 to 86, with low scores indicating extremely late chronotypes.

Statistical analysis

Continuous participant characteristics were summarized using mean and standard deviation (SD). Categorical variables, such as educational level, were summarized by count and percent. In line with the CONSORT statement, baseline differences between the two treatment arms were not tested [69]. For treatment effect analyses, we applied an intention-to-treat procedure, since none of the participants could switch to a different condition, and we included all observations of all participants until the study ended or the participant(s) dropped out of the study.

For peer review only

The primary outcome was changes in SIGH-SAD rating scale scores over time. Secondary outcomes were changes in HAM-D and EPDS rating scale scores over time. Analyses were conducted using general linear mixed modelling analyses. In a series of random-intercept models, we included time (continuous), allocation, and time x allocation interaction-term as an effect measure of allocation on the course of depression rating scale scores. The standardized baseline score was included in the model, since baseline depression severity is an important predictor for treatment outcome [70]. We studied the treatment effect for both the intervention period and follow-up period (two months postpartum).

Primary analyses were first crude, then adjusted. As adjusted primary analyses, we calculated propensity scores based on patient characteristics (psychiatric history, ethnicity, level of education, an unplanned pregnancy, maternal age, parity, gestational age, duration of actual depression, and other psychiatric or psychotherapeutic treatment interventions). Next, we adjusted separately for chronotype and the month of treatment. By means of sensitivity analyses, we repeated the primary analyses with last observation carried forward data imputation. As post-hoc analyses, we repeated the crude analyses for women with good compliance (<7 missed treatments) and for women with most severe depressive symptomatology (based on median split baseline SIGH-SAD scores). Effect parameters were supplied with a 95% confidence interval (CI).

Additionally, we tested responders versus non-responders with Fisher's exact test, where response was defined as a ≥50% decrease to a final score of ≤8 on the 17-item HAM-D and ≤5 on the EPDS at the end of the intervention period.

Data was analyzed using SPSS 21.0 (IBM Corporation, Chicago, IL, USA). Statistical significance was defined as $p<.05$.

Results

Demographic and clinical characteristics

In total, 283 women were referred to the study. The majority of the participants (82%) were recruited via (social) media. Of these referrals, we included and randomized 67 women, with 33 allocated to BLT and 34 to DRLT. In total, eleven women dropped out during the study, of whom five in the BLT group. Ten

For peer review only

women dropped out in the intervention period, one at ten weeks after treatment. Figure 1 shows a flow-chart of the entire study sample.

Table 2 shows the participant characteristics at the time of inclusion. At inclusion, the mean (SD) of the SIGH-SAD was 26.5 (7.2), of the 17-item HAM-D was 16.9 (5.3) and of the EPDS was 16.1 (4.8). Median scores were respectively 27, 17 and 16.

The most common comorbidity was anxiety (25.4%), followed by obsessive compulsive disorder (17.9%), PTSS (11.9%), and social phobia (11.9%). Various somatic comorbidities were reported, such as asthma, Guillain-Barré syndrome, and fibromyalgia.

During the course of this study, as part of the care as usual, eleven additional women started with psychotherapy: three women in the intervention period, one after the intervention period during pregnancy, and seven in the postpartum period. During the entire study, four additional women started with psychotropic medication: one woman started with an SSRI in the intervention period and one woman in the postpartum period (both sertraline), one with an antipsychotic (quetiapine) and one with a benzodiazepine (temazepam) postpartum. Of one participant, the dose of the SSRI was increased in the postpartum period (escitalopram).

Compliance

Self-reported compliance was somewhat higher in the BLT group, compared to the DRLT group. Amongst the women treated with BLT, eight women (24.2%) never missed a treatment, in contrast to three women (8.8%) in the DRLT group. Sixteen women (48.5%) treated with BLT missed a maximum of six treatments, compared to twenty women (58.9%) in the DRLT group. In both groups, two women missed seven to thirteen treatments in the intervention period. One woman treated with BLT and two with DRLT missed fourteen or more treatments. One woman treated with BLT and two with DRLT missed the final two weeks of treatment, the first one due to complete remission of her symptoms.

Maintaining blinding

Before treatment, three women (4.8%) did not expect any effect from light therapy for their depressive symptoms. All other participants expected a (small) positive effect. After treatment, one participant treated

For peer review only

with BLT (3.0%) and three women in the group treated with DRLT (8.8%) thought they were treated with placebo treatment. All other women had no specific ideas about their allocation.

Treatment effect

Supplementary Table 2 shows the observed median SIGH-SAD, HAM-D, and EPDS scores over the course of the study. In the women treated with BLT, median depression scores decreased by 42.6% (SIGH-SAD), 53.1% (HAM-D), and 40.6% (EPDS) in the intervention period. In the DRLT group, this was respectively 50.9%, 66.7%, and 59.4%. After women stopped with light treatment, median scores continued to decrease for all questionnaires in both groups, three and ten weeks after treatment. At two months postpartum, women treated with BLT showed no increase in EPDS scores, whereas women treated with DRLT showed an increase in EPDS-scores. For both SIGH-SAD and HAM-D scores, a decrease was observed in both treatment arms.

We also calculated the median improvement scores without the baseline score. For women treated with BLT, these were 6.1% (SIGH-SAD), 16.7% (HAM-D), and 13.6% (EPDS). For women treated with DRLT, this was respectively 31.6%, 40%, and 45.8%.

No statistically significant difference was found between the two treatment arms for the intervention period, nor for the entire study. For the SIGH-SAD, our primary endpoint, we found β=-0.68 (95% CI -1.84, 0.49) for the intervention period and β=-0.16 (95% CI -0.82, 0.51) for the entire study (Figure 2 and Table 3). Adjusted primary analyses, where we repeated our primary analyses adjusted for propensity scores, and sensitivity analyses with imputed data did not show any other findings (Supplementary Table 3). Adjustment for chronotype and month of treatment did not change our findings as well. Post-hoc analyses, where we repeated the analyses for women with higher treatment compliance and for women with higher symptom severity at baseline, did not show a statistically significant difference between the two treatment arms (Supplementary Table 3).

For the HAM-D, 13 participants in the BLT group and 17 participants in the DRLT group were considered responders. This was respectively 11 and 9 when measured with the EPDS. When we studied responders versus non-responders, we found no statistically significant differences for both HAM-D scores (p=.46) and EPDS scores (p=.60).

For peer review only

Side effects

For women treated with BLT, the most frequently reported side effect was headaches (30.3%), followed by sleep problems (12.1%) and nausea (6.1%). For women treated with DRLT, the most reported side effect was headaches (20.6%), followed by sleep problems (8.9%) and irritable eyes (5.9%). Side effects were not reported more often by women treated with BLT, compared to DRLT (p=0.52). Most side effects were experienced for a maximum of three days. None of the women suffered from any (hypo)manic symptoms. We reduced the treatment duration for five women to 20 minutes daily due to their side effects. Interestingly, two women dropped out of the study due to side effects, but only in the DRLT group.

Acceptability and satisfaction

The majority of women experienced a (small) positive effect for their depressive symptoms (78.6% BLT; 61.5% DRLT; p=0.58). All participants found the lamp (very) easy in use. Most women found the light therapy pleasant (57.1% BLT; 50% DRLT; p=0.49). Twenty-six women reported that it was (very) easy to plan the light therapy in the morning (42.9% BLT; 53.8% DRLT; p=0.43). Thirty-two women reported that they would like to use light therapy outside of the study (57.1% BLT; 61.5% DRLT; p=0.79). On average, women reported it was likely they would recommend the light therapy to others (BLT mean 8.0, SD 1.3; DRLT mean 7.0, SD 2.7; p=0.08).

Discussion

We conducted a randomized controlled trial, evaluating the effectiveness of BLT in a sample of 67 pregnant women with major depressive disorder, compared to DRLT. We found no statistically significant difference between BLT and DRLT on depressive symptoms. Median depression scores decreased by 40.6-53.1% during the intervention in the women treated with BLT and by 50.9-66.7% in the women treated by DRLT.

Effects in the current study

For peer review only

This level of improvement is comparable to the studies by Oren *et al*. [51] and Corral *et al*. [71] who both found a reduction in mean depression scores of 49%. Oren *et al*. conducted an open trial in an antepartum population, whereas Corral *et al*. conducted a randomized controlled trial among women with a postpartum depression. Similar to Corral *et al*., we did not find a statistically significant difference between the effective and placebo conditions. The median improvement in the DRLT group can be explained by placebo effects, which could also be the case in the BLT group. A meta-analysis showed that the placebo response in antidepressant trials is approximately 68% [72], although this effect is not clear yet in light therapy trials specifically. Secondly, the improvement in both groups can be explained by non-specific treatment effects such the structure offered by the study [43], the interaction with the researchers, or increased awareness and self-care resulting from participating in the study. A systematic review on various studies in treating antepartum depression with a control condition showed that these trials often show a considerable reduction in symptom scores in both treatment arms [39]. Furthermore, it might be that symptoms decrease related to the course of pregnancy, spontaneous remission, or regression to the mean. A meta-analysis showed that untreated depressive symptoms could decrease by 10-15%, on average [73]. However, untreated depression during pregnancy is an important predictor for postpartum depression [74]. We calculated the improvement of the depressive symptoms without the baseline scores, to study whether the improvement was especially notable in the first week of treatment. We found that the improvement was less, especially in the group treated with BLT, which may pinpoint to regression to the mean. For example, women may have the feeling of ‘finally being heard’, or feeling empowered about doing something about their symptoms, which may explain these findings.

Corral *et al*. mentioned that several participants commented positively on having 30 minutes of “quiet time” on a daily basis. Several of our participants mentioned this as well, which could reflect sinking into a state of more relaxation or more mindfulness which may have contributed to the improvement in both groups. Two meta-analyses showed that mindfulness-based therapy is an effective treatment for a variety of psychological problems [75,76]. An earlier pilot study and an open study of mindfulness also showed positive effects on mood specifically in pregnant women [77,78]. Corral *et al*. mentioned that many postpartum women are motivated to access recourses, such as psychological treatment, which could have exerted non-specific treatment effects. In their study however, no participant took part in any

For peer review only

treatment during the study. In our study, several women started psychotherapy or antidepressant medication. However, adjustment for any intervention did however not change our findings. Finally, it has been shown earlier in healthy volunteers that treatment with similar conditions as our placebo therapy might actually have some effects in melatonin suppression [79], which could explain why we actually see a decrease of symptoms in the DRLT group.

Differences with literature

The results of this study differ from the randomized controlled trials by Epperson *et al.* [52] and Wirz-Justice *et al*. [53], who did find superiority of bright light therapy over placebo in an antepartum population. Wirz-Justice *et al*. included only clinical patients and found that BLT had more effects in severe patients in their study. However, mean baseline SIGH-SAD score in the Wirz-Justice et al. and Epperson et al. studies were 27.7 and 28.1, respectively, which were not clinically relevant different from the present study (26.5). Additionally, we included baseline depression scores in our model, which did not change our findings. Also, post-hoc analyses, where we repeated the analyses for women with higher baseline severity, did not show any significant findings.

Both Epperson *et al*. and Wirz-Justice *et al*. treated their patients for 1 hour a day and within 10 minutes of habitual wake-up time, which is different from the present study. Thus far, no studies have been executed comparing the effectiveness of shorter versus longer exposure to bright light in non-seasonal depression. Possibly, more light output in the BLT group would be necessary to show superiority of BLT over DRLT in a pregnant population. However, other studies that treated patients for 30 minutes also did show a statistical significant difference between the effective and the placebo intervention in non-seasonal depression [46]. One must keep in mind that these studies have been done in non-pregnant populations and different – yet unknown – underlying mechanisms may play a part during pregnancy, such as hormonal fluctuations and a shift in social role.

Our placebo condition, in which the possible effect of DRLT could be questioned, is not a plausible explanation for not finding a statistically significant effect between the treatment arms. Epperson *et al.* used a placebo condition with 500 lux white light, which is questionable as a placebo, for white light of 100 lux is able to phase-shift human circadian rhythms [80]. Since this study found a significant

For peer review only - http://bmjopen.bmj.com/site/about/guidelines.xhtml

improvement in women treated with BLT when compared to this placebo, it is unlikely that the settings of our placebo would explain failing to achieve a significant difference between the two treatment arms. In the study by Corral *et al.*, depression scores worsened after withdrawal of treatment, indicating that spontaneous remission would be less likely. However, in the present study, median depressions scores of all questionnaires continued to improve after withdrawal of treatment in both groups, indicating that spontaneous remission in both groups is a possible explanation for this finding.

Strengths and limitations

Internationally, we conducted the largest randomized controlled trial studying light therapy in pregnant women with a depression. Moreover, we conducted various follow up measurements, including postpartum, to study the effects of withdrawal of treatment and to study whether treatment during pregnancy would protect against postpartum depression. Another strength is using a single assessor to diagnose depression. Moreover, the setting of treatment was within a real world setting. Finally, a strength of this study was the comprehensive assessment of side effects, as well as acceptability and satisfaction of treatment.

The main limitation of our study was that an unforeseen lack of resources prevented us from including 150 participants, as we aimed to do according to our sample size calculation [55], which enables us to find only large treatment effects [55]. Another limitation is the fact that depressive symptoms during the study are assessed by questionnaires, rather than diagnostic criteria. Also, information about psychiatric history was collected via an interview and not through medical records, which may be influenced by recall bias. Moreover, various covariates are self-reported, such as BMI, substance use and medication. We noticed a different attrition rate at T3 (10 weeks after treatment) and P1 (2 months postpartum). At T3, this is due to the fact that more women treated with DRLT already gave birth at T3, which resulted in missing data. We do not have an explanation for the different attrition rate at P1. We cannot rule out the possibility that these differences in attrition might have impacted our follow-up results. However, our sensitivity analyses indicate our follow-up results to be robust for differences between the conditions and data imputation.

Conclusions

For peer review only

BLT has been shown effective in treating non-seasonal depression [46] and in women with antepartum depression as well [52,53]. In the present study, depressive symptoms of pregnant women with depression improved in both treatment arms after 6 weeks of treatment. Given the very mild and short-lived side effects, the major improvement in a short time period, the high acceptability of the participants, the low costs, and the direct availability, more studies to the effectiveness of BLT during pregnancy are warranted. It is important to determine whether the responses observed in the present study represent true treatment effects, non-specific treatment responses, placebo effects, or a combination of these. This could be done by studying biological outcomes, such as cortisol and melatonin levels, which might show a statistically significant difference between the two treatment arms irrespective of perceived symptoms of depression. Additionally, it might show an indication of the positive effects of light therapy on the circadian rhythm and its inhibiting effects on HPA-axis hyperactivity.

Acknowledgements

We would like to thank all participants for participating in the study. We would also like to thank all general practitioners, midwifes, gynaecologists, psychiatrists and psychologists for their help with the recruitment. We are grateful for all co-workers, students and assistants who contributed to the data collection in this study: Nina Molenaar, PhD, Marlies Brouwer, PhD, Leo Genet, MSc, Sophie de Droog, MSc, Sofie Koomen, MSc, Diewertje Houtman, MSc, Maria Zepeda, MSc, Nicolle Croes, MSc, Rianne Winters, MSc. Lisanne van Kesteren, BSc, Finn Stofkoper, BSc, Indira Schouten, MSc and Mieke Roukema, MSc.

Funding

MLB received funding from the 'Light, Cognition, Behaviour and Health' program of The Netherlands Organization for Health Research and Development (NWO; The Hague, The Netherlands), in collaboration with Signify Research (grant number 058-14-003) to fund the current study.

Competing interests

Author JS is employed by Signify Research. The lamps used in this study were provided by Signify Research.

For peer review only

502

503 **Author's contributions**

504 MLB is the project's principle investigator and initiator of the study, obtained funding and designed the
505 study. BB was responsible for recruiting and counselling participants, running the study and collecting
506 data. AK is the project's methodologist and executed the primary statistical analysis. HB and EK were
507 involved in the recruitment of the study. JS provided support. AK, WH and MLB supervised the study. BB,
508 AK and MLB prepared the original draft. All authors reviewed, edited and approved the final manuscript.

509

510 **Data availability statement**

511 The datasets used and/or analysed during the current study are available from the corresponding author
512 MLB on reasonable request.

513

514 **Word count**

515 5,608

516

517 **References**

518 1. Woody CA, Ferrari AJ, Siskind DJ, Whiteford HA, Harris MG. A systematic review and meta-
519 regression of the prevalence and incidence of perinatal depression. *J Affect Disord* 2017; **219**: 86-92.
520 2. Bais B, de Groot N, Grootendorst-van Mil NH, et al. Seasonality of depressive symptoms during
521 pregnancy. *Psychiatry Res* 2018; **268**: 257-62.
522 3. Lancaster CA, Gold KJ, Flynn HA, Yoo H, Marcus SM, Davis MM. Risk factors for depressive
523 symptoms during pregnancy: a systematic review. *Am J Obstet Gynecol* 2010; **202**(1): 5-14.
524 4. Räisänen S, Lehto SM, Nielsen HS, Gissler M, Kramer MR, Heinonen S. Risk factors for and
525 perinatal outcomes of major depression during pregnancy: a population-based analysis during 2002-2010
526 in Finland. *BMJ Open* 2014; (11): DOI:10.1136/bmjopen-2014-004883.
527 5. Seth S, Lewis AJ, Galbally M. Perinatal maternal depression and cortisol function in pregnancy
528 and the postpartum period: a systematic literature review. *BMC Pregnancy Childbirth* 2016; **16**(1): 124.

For peer review only

529 6. Leff-Gelman P, Mancilla-Herrera I, Flores-Ramos M, et al. The Immune System and the Role of Inflammation in Perinatal Depression. *Neurosci Bull* 2016; **32**(4): 398-420.

531 7. Osborne LM, Monk C. Perinatal depression--the fourth inflammatory morbidity of pregnancy?: Theory and literature review. *Psychoneuroendocrinology* 2013; **38**(10): 1929-52.

533 8. Milgrom J, Gemmill AW, Bilszta JL, et al. Antenatal risk factors for postnatal depression: a large prospective study. *J Affect Disord* 2008; **108**: 147-57.

535 9. Grote NK, Bridge JA, Gavin AR, Melville JL, Iyengar S, Katon WJ. A meta-analysis of depression during pregnancy and the risk of preterm birth, low birth weight, and intrauterine growth restriction. *Arch Gen Psychiatry* 2010; **67**(10): 1012-24.

538 10. Jarde A, Morais M, Kingston D, et al. Neonatal outcomes in women with untreated antenatal depression compared with women without depression: a systematic review and meta-analysis. *JAMA Psychiatry* 2016: DOI:10.1001/jamapsychiatry.2016.0934.

541 11. Talge NM, Neal C, Glover V, the Early Stress, Translational Research and Prevention Science Network: Fetal and Neonatal Experience on Child and Adolescent Mental Health. Antenatal maternal stress and long-term effects on child neurodevelopment: how and why? *J Child Psychol Psychiatry* 2007; **48**(3-4): 245-61.

545 12. Hentges RF, Graham SA, Plamondon A, Tough S, Madigan S. A Developmental Cascade from Prenatal Stress to Child Internalizing and Externalizing Problems. *J Pediatr Psychol* 2019.

547 13. Pearson RM, Evans J, Kounali D, et al. Maternal depression during pregnancy and the postnatal period: risks and possible mechanisms for offspring depression at age 18 years. *JAMA Psychiatry* 2013; **70**(12): 1312-9.

550 14. Glover V, O'Connor TG, O'Donnell K. Prenatal stress and the programming of the HPA axis. *Neurosci Biobehav Rev* 2010; **35**(1): 17-22.

552 15. Dierckx B, Tulen JH, van den Berg MP, et al. Maternal psychopathology influences infant heart rate variability: Generation R Study. *Psychosom Med* 2009; **71**(3): 313-21.

554 16. Goedhart G, Vrijkotte TG, Roseboom TJ, van der Wal MF, Cuijpers P, Bonsel GJ. Maternal cortisol and offspring birthweight: results from a large prospective cohort study. *Psychoneuroendocrinology* 2010; **35**(5): 644-52.

For peer review only

557 17. Henrichs J, Schenk JJ, Roza SJ, et al. Maternal psychological distress and fetal growth trajectories: the Generation R Study. *Psychol Med* 2010; **40**(4): 633-43.

559 18. Zijlmans MA, Riksen-Walraven JM, de Weerth C. Associations between maternal prenatal cortisol concentrations and child outcomes: A systematic review. *Neurosci Biobehav Rev* 2015; **53**: 1-24.

561 19. Steegers EA, Barker ME, Steegers-Theunissen RP, Williams MA. Societal Valorisation of New Knowledge to Improve Perinatal Health: Time to Act. *Paediatr Perinat Epidemiol* 2016; **30**(2): 201-4.

563 20. Byatt N, Deligiannidis KM, Freeman MP. Antidepressant use in pregnancy: a critical review focused on risks and controversies. *Acta Psychiatr Scand* 2013; **127**(2): 94-114.

565 21. Hanley GE, Oberlander TF. The effect of perinatal exposures on the infant: antidepressants and depression. *Best Pract Res Clin Obstet Gynaecol* 2014; **28**(1): 37-48.

567 22. Kieler H, Artama M, Engeland A, et al. Selective serotonin reuptake inhibitors during pregnancy and risk of persistent pulmonary hypertension in the newborn: population based cohort study from the five Nordic countries. *BMJ* 2012; **344**: d8012.

570 23. Simoncelli M, Martin BZ, Berard A. Antidepressant use during pregnancy: a critical systematic review of the literature. *Curr Drug Saf* 2010; **5**(2): 153-70.

572 24. Battle CL, Salisbury AL, Schofield CA, Ortiz-Hernandez S. Perinatal antidepressant use: understanding women's preferences and concerns. *J Psychiatr Pract* 2013; **19**(6): 443-53.

574 25. Kothari A, de Laat J, Dulhunty JM, Bruxner G. Perceptions of pregnant women regarding antidepressant and anxiolytic medication use during pregnancy. *Australas Psychiatry* 2019; **27**(2): 117-20.

577 26. Molenaar NM, Brouwer ME, Duvekot JJ, et al. Antidepressants during pregnancy: Guideline adherence and current practice amongst Dutch gynaecologists and midwives. *Midwifery* 2018; **61**: 29-35.

579 27. Molenaar NM, Kamperman AM, Boyce P, Bergink V. Guidelines on treatment of perinatal depression with antidepressants: An international review. *Aust N Z J Psychiatry* 2018; **52**(4): 320-7.

581 28. Bakker MK, Kolling P, van den Berg PB, de Walle HE, de Jong van den Berg LT. Increase in use of selective serotonin reuptake inhibitors in pregnancy during the last decade, a population-based cohort study from the Netherlands. *Br J Clin Pharmacol* 2008; **65**(4): 600-6.

For peer review only

29. Molenaar NM, Lambregtse-van den Berg MP, Bonsel GJ. Dispensing patterns of selective serotonin reuptake inhibitors before, during and after pregnancy: a 16-year population-based cohort study from the Netherlands. *Arch Womens Ment Health* 2019.

30. Charlton RA, Jordan S, Pierini A, et al. Selective serotonin reuptake inhibitor prescribing before, during and after pregnancy: a population-based study in six European regions. *Bjog-Int J Obstet Gy* 2015; **122**(7): 1010-20.

31. Cooper WO, Willy ME, Pont SJ, Ray WA. Increasing use of antidepressants in pregnancy. *American Journal of Obstetrics and Gynecology* 2007; **196**(6): 544-5.

32. Jimenez-Solem E, Andersen JT, Petersen M, et al. Prevalence of Antidepressant Use during Pregnancy in Denmark, a Nation-Wide Cohort Study. *Plos One* 2013; **8**(4).

33. van Gelder MMHJ, Bos JHJ, Roeleveld N, de Jong-van den Berg LTW. Drugs associated with teratogenic mechanisms. Part I: dispensing rates among pregnant women in the Netherlands, 1998-2009. *Hum Reprod* 2014; **29**(1): 161-7.

34. Ververs T, Kaasenbrood H, Visser G, Schobben F, de Jong-van den Berg L, Egberts T. Prevalence and patterns of antidepressant drug use during pregnancy. *Eur J Clin Pharmacol* 2006; **62**(10): 863-70.

35. Ailes EC, Simeone RM, Dawson AL, Petersen EE, Gilboa SM. Using insurance claims data to identify and estimate critical periods in pregnancy: An application to antidepressants. *Birth Defects Res A Clin Mol Teratol* 2016; **106**(11): 927-34.

36. Andrade SE, Reichman ME, Mott K, et al. Use of selective serotonin reuptake inhibitors (SSRIs) in women delivering liveborn infants and other women of child-bearing age within the U.S. Food and Drug Administration's Mini-Sentinel program. *Arch Womens Ment Health* 2016; **19**(6): 969-77.

37. Taylor LG, Thelus Jean R, Gordon G, Fram D, Coster T. Development of a mother-child database for drug exposure and adverse event detection in the Military Health System. *Pharmacoepidemiol Drug Saf* 2015; **24**(5): 510-7.

38. Hanley GE, Mintzes B. Patterns of psychotropic medicine use in pregnancy in the United States from 2006 to 2011 among women with private insurance. *BMC Pregnancy Childbirth* 2014; **14**: 242.

For peer review only

For peer review only - http://bmjopen.bmj.com/site/about/guidelines.xhtml

39. van Ravesteyn LM, Lambregtse-van den Berg MP, Hoogendijk WJ, Kamperman AM. Interventions to treat mental disorders during pregnancy: A systematic review and multiple treatment meta-analysis. *PLoS One* 2017; **12**(3): e0173397.

40. Blume C, Garbazza C, Spitschan M. Effects of light on human circadian rhythms, sleep and mood. *Somnologie* 2019.

41. LeGates TA, Fernandez DC, Hattar S. Light as a central modulator of circadian rhythms, sleep and affect. *Nature Reviews Neuroscience* 2014; **15**(7): 443-54.

42. Prayag AS, Münch M, Aeschbach D, Chellappa SL, Gronfier C. Light modulation of human clocks, wake and sleep. *Clocks & Sleep* 2019; **1**: 193-208.

43. Germain A, Kupfer DJ. Circadian rhythm disturbances in depression. *Hum Psychopharmacol* 2008; **23**(7): 571-85.

44. Lam RW, Levitan RD. Pathophysiology of seasonal affective disorder: a review. *J Psychiatry Neurosci* 2000; **25**(5): 469-80.

45. Magnusson A, Boivin D. Seasonal affective disorder: an overview. *Chronobiol Int* 2003; **20**(2): 189-207.

46. Tuunainen A, Kripke DF, Endo T. Light therapy for non-seasonal depression. *Cochrane Database Syst Rev* 2004.

47. Golden RN, Gaynes BN, Ekstrom RD, et al. The efficacy of light therapy in the treatment of mood disorders: a review and meta-analysis of the evidence. *Am J Psychiatry* 2005; **162**(4): 656-62.

48. Al-Karawi D, Jubair L. Bright light therapy for nonseasonal depression: Meta-analysis of clinical trials. *J Affect Disord* 2016; **198**: 64-71.

49. Perera S, Eisen R, Bhatt M, et al. Light therapy for non-seasonal depression: systematic review and meta-analysis. *BJPsych Open* 2016; **2**(2): 116-26.

50. Martensson B, Pettersson A, Berglund L, Ekselius L. Bright white light therapy in depression: A critical review of the evidence. *J Affect Disord* 2015; **182**: 1-7.

51. Oren DA, Wisner KL, Spinelli M, et al. An open trial of morning light therapy for treatment of antepartum depression. *Am J Psychiatry* 2002; **159**(4): 666-9.

For peer review only

52. Epperson CN, Terman M, Terman JS, et al. Randomized clinical trial of bright light therapy for antepartum depression: preliminary findings. *J Clin Psychiatry* 2004; **65**(3): 421-5.

53. Wirz-Justice A, Bader A, Frisch U, et al. A randomized, double-blind, placebo-controlled study of light therapy for antepartum depression. *J Clin Psychiatry* 2011; **72**(7): 986-93.

54. Ioannidis JP. Why most published research findings are false. *PLoS Med* 2005; **2**(8): e124.

55. Bais B, Kamperman AM, van der Zwaag MD, et al. Bright light therapy in pregnant women with major depressive disorder: study protocol for a randomized, double-blind, controlled clinical trial. *BMC Psychiatry* 2016; **16**(1): 381.

56. First MB GM, Spitzer RL, Williams JBW. User's guide for the SCID-I. Structured clinical interview for DSM-IV TR axis I disorders (research version). New York: New York Psychiatric Institute; 2002.

57. Lieverse R, Van Someren EJ, Nielen MM, Uitdehaag BM, Smit JH, Hoogendijk WJ. Bright light treatment in elderly patients with nonseasonal major depressive disorder: a randomized placebo-controlled trial. *Arch Gen Psychiatry* 2011; **68**(1): 61-70.

58. Kreidler SM, Muller KE, Grunwald GK, et al. GLIMMPSE: Online Power Computation for Linear Models with and without a Baseline Covariate. *J Stat Softw* 2013; **54**(10).

59. Meesters Y, Dekker V, Schlangen LJ, Bos EH, Ruiter MJ. Low-intensity blue-enriched white light (750 lux) and standard bright light (10,000 lux) are equally effective in treating SAD. A randomized controlled study. *BMC Psychiatry* 2011; **11**: 17.

60. Project TAI. ALEA Randomisation Software (Version 2.2). Amsterdam: Netherlands Cancer Institute; 2006. p. http://tenalea.net/.

61. Bockting CL, Hollon SD, Jarrett RB, Kuyken W, Dobson K. A lifetime approach to major depressive disorder: The contributions of psychological interventions in preventing relapse and recurrence. *Clin Psychol Rev* 2015; **41**: 16-26.

62. Williams JBW LM, Rosenthal NE, Amira L, Terman M. Structured Interview Guide for the Hamilton Depression Rating Scale, Seasonal Affective Disorders version (SIGH-SAD). New York: New York Psychiatric Institute; 1988.

For peer review only

63. Pjrek E, Friedrich ME, Cambioli L, et al. The Efficacy of Light Therapy in the Treatment of Seasonal Affective Disorder: A Meta-Analysis of Randomized Controlled Trials. *Psychother Psychosom* 2020; **89**(1): 17-24.

64. Cox JL, Holden JM, Sagovsky R. Detection of postnatal depression. Development of the 10-item Edinburgh Postnatal Depression Scale. *Br J Psychiatry* 1987; **150**: 782-6.

65. Bergink V, Kooistra L, Lambregtse-van den Berg MP, et al. Validation of the Edinburgh Depression Scale during pregnancy. *J Psychosom Res* 2011; **70**(4): 385-9.

66. Roenneberg T, Wirz-Justice A, Merrow M. Life between clocks: daily temporal patterns of human chronotypes. *J Biol Rhythms* 2003; **18**(1): 80-90.

67. Merikanto I, Kronholm E, Peltonen M, Laatikainen T, Vartiainen E, Partonen T. Circadian preference links to depression in general adult population. *J Affect Disord* 2015; **188**: 143-8.

68. Merikanto I, Lahti T, Kronholm E, et al. Evening types are prone to depression. *Chronobiol Int* 2013; **30**(5): 719-25.

69. Schulz KF, Altman DG, Moher D, Group C. CONSORT 2010 Statement: updated guidelines for reporting parallel group randomised trials. *BMC Med* 2010; **8**: 18.

70. Friedman ES, Davis LL, Zisook S, et al. Baseline depression severity as a predictor of single and combination antidepressant treatment outcome: results from the CO-MED trial. *Eur Neuropsychopharmacol* 2012; **22**(3): 183-99.

71. Corral M, Wardrop AA, Zhang H, Grewal AK, Patton S. Morning light therapy for postpartum depression. *Arch Womens Ment Health* 2007; **10**(5): 221-4.

72. Rief W, Nestoriuc Y, Weiss S, Welzel E, Barsky AJ, Hofmann SG. Meta-analysis of the placebo response in antidepressant trials. *J Affect Disord* 2009; **118**(1-3): 1-8.

73. Posternak MA, Miller I. Untreated short-term course of major depression: a meta-analysis of outcomes from studies using wait-list control groups. *J Affect Disord* 2001; **66**(2-3): 139-46.

74. Yazici E, Kirkan TS, Aslan PA, Aydin N, Yazici AB. Untreated depression in the first trimester of pregnancy leads to postpartum depression: high rates from a natural follow-up study. *Neuropsychiatr Dis Treat* 2015; **11**: 405-11.

For peer review only

75. Khoury B, Lecomte T, Fortin G, et al. Mindfulness-based therapy: a comprehensive meta-analysis. *Clin Psychol Rev* 2013; **33**(6): 763-71.

76. Hofmann SG, Sawyer AT, Witt AA, Oh D. The effect of mindfulness-based therapy on anxiety and depression: A meta-analytic review. *J Consult Clin Psychol* 2010; **78**(2): 169-83.

77. Dimidjian S, Goodman SH, Felder JN, Gallop R, Brown AP, Beck A. An open trial of mindfulness-based cognitive therapy for the prevention of perinatal depressive relapse/recurrence. *Arch Womens Ment Health* 2015; **18**(1): 85-94.

78. Vieten C, Astin J. Effects of a mindfulness-based intervention during pregnancy on prenatal stress and mood: results of a pilot study. *Arch Womens Ment Health* 2008; **11**(1): 67-74.

79. Nagare R, Plitnick B, Figueiro MG. Effect of exposure duration and light spectra on nighttime melatonin suppression in adolescents and adults. *Light Res Technol* 2019; **51**(4): 530-43.

80. Zeitzer JM, Dijk DJ, Kronauer R, Brown E, Czeisler C. Sensitivity of the human circadian pacemaker to nocturnal light: melatonin phase resetting and suppression. *J Physiol* 2000; **526 Pt 3**: 695-702.

Table 1. Inclusion and exclusion criteria for the Bright Up Study.

**Inclusion criteria**	**Women**
	18-45 years of age
	12-32 weeks pregnant (as confirmed by ultrasound)
	Current DSM-5 diagnosis of depressive disorder (as assessed by the SCID*)
**Exclusion criteria**	Insufficient proficiency in Dutch or English
	Multiple pregnancy
	Current use of antidepressants shorter than 2 months
	Lifetime diagnosis of bipolar I or II disorder
	Any psychotic episode
	Current substance abuse
	Current primary anxiety disorder

For peer review only

	Recent history of suicide attempt
	Current shift-work
	Somatic and/or obstetric conditions that override study participation
	Previous treatment with BLT
	Eye condition (macular degeneration, eye diseases, recent eye surgery)

* SCID = Structured Clinical Interview for DSM disorders

Table 2. Overview of participant characteristics at inclusion.

	**BLT (n=33)**	**DRLT (n=34)**
**Age in years, mean (SD)**	31.9 (4.4)	31.9 (5.3)
**Gestational age in weeks, mean (SD)**	20.6 (6.2)	19.7 (6.3)
**Ethnicity**		
**Dutch**	27 (81.8%)	26 (76.5%)
**Other**	6 (19.2%)	8 (33.5%)
**Marital status**		
**Married or cohabiting**	33 (100%)	32 (94.1%)
**Committed relationship, not cohabiting**	0 (0%)	1 (2.9%)
**Single**	0 (0%)	1 (2.9%)
**Education**		
**Elementary or (pre-)vocational education**	11 (33.3%)	13 (38.2%)
**Higher professional education**	8 (24.2%)	11 (32.4%)
**(Pre-) academic education**	14 (42.4%)	10 (29.4%)
**Parity**		
**Nulliparous**	15 (45.5%)	20 (58.8%)
**Primiparous**	13 (39.4%)	9 (26.5%)
**Multiparous**	5 (15.2%)	5 (14.7%)
**BMI in kg/m^2 or st/ft^2, mean (SD)**	25.5 (4.5)	26.3 (5.4)

For peer review only

**Planned pregnancy**	22 (66.7%)	22 (64.7%)
**Antidepressant medication**	3 (9.1%)	5 (14.7%)
**Sleep medication**	3 (9.1%)	2 (5.9%)
**Psychotherapy**	14 (48.5%)	16 (47.1%)
**Comorbidities**		
**0**	17 (51.5%)	13 (38.2%)
**1**	9 (27.3%)	13 (38.2%)
**>1**	7 (21.2%)	8 (23.5%)
**Duration of depression in weeks, mean (SD)**	24.6 (16.9)	45.1 (121.9)
**Depressive episodes in past**		
**0**	12 (36.4%)	11 (32.4%)
**1**	9 (27.2%)	14 (41.2%)
**>1**	12 (36.4%)	9 (26.5%)
**Chronotype**		
**Early (extremely, moderately and slightly)**	20 (80%)	25 (92.6%)
**Normal**	1 (4%)	1 (3.7%)
**Late (extremely, moderately and slightly)**	4 (16%)	1 (3.7%)

BLT = bright light therapy; DRLT = dim red light therapy

Table 3. Effects of allocation on the course of depressive symptoms through the intervention period and follow-up (until two months postpartum): crude analysis.

	**β (95% CI) of intervention***	**β (95% CI) of follow-up****
**SIGH-SAD**	-0.68 (-1.84, 0.49)	-0.16 (-0.82, 0.51)
**HAM-D**	-0.18 (-0.74, 0.37)	0.04 (-0.29, 0.37)
**EPDS**	0.01 (-0.51, 0.53)	-0.05 (-0.35, 0.24)

* From start of study until end of treatment; ** From start of study until follow-up 2 months postpartum

Figure legends

For peer review only

Figure 1. Flow-chart of the Bright Up study.

Figure 2. Estimated marginal means of depression scores in women with antepartum depression until two months postpartum. Shown are SIGH-SAD, HAM-D and EPDS scores. Black lines represent treatment with BLT, gray lines with DRLT. Bars represent standard error of the mean.

BLT = bright light therapy; DRLT = dim red light therapy; SIGH- SAD = Structured Interview Guide for the Hamilton Depression Scale – Seasonal Affective Disorder version; HAM-D = Hamilton Rating Scale for Depression; EPDS = Edinburgh Postnatal Depression Scale; T0 = baseline, before treatment; T0+1, T0+2 ... T0+5 = weeks during intervention period; T1 = end of treatment; T2 = 3 weeks after end of treatment; T3 = 10 weeks after end of treatment; P1 = 2 months postpartum

For peer review only

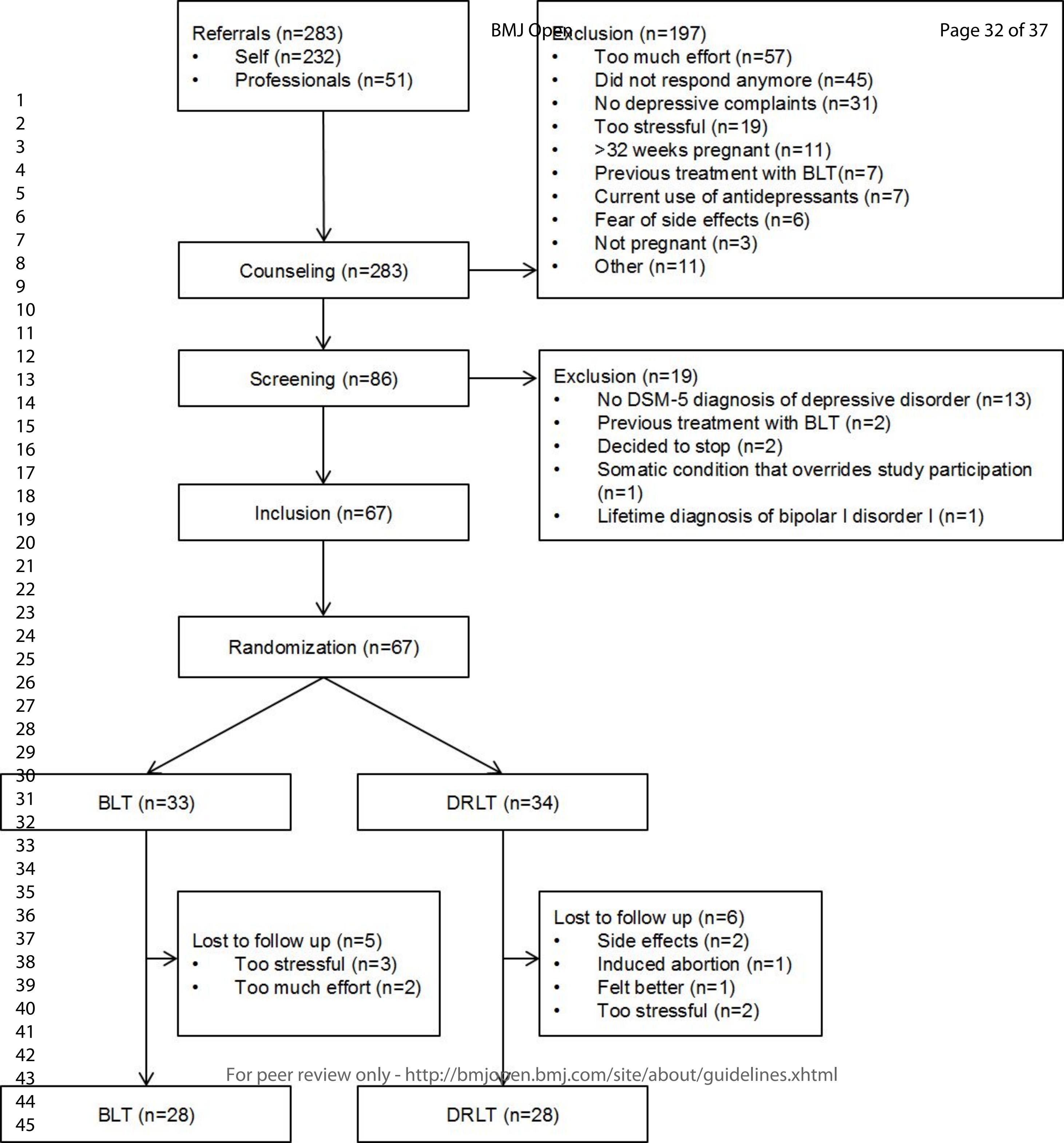

For peer review only - http://bmjopen.bmj.com/site/about/guidelines.xhtml

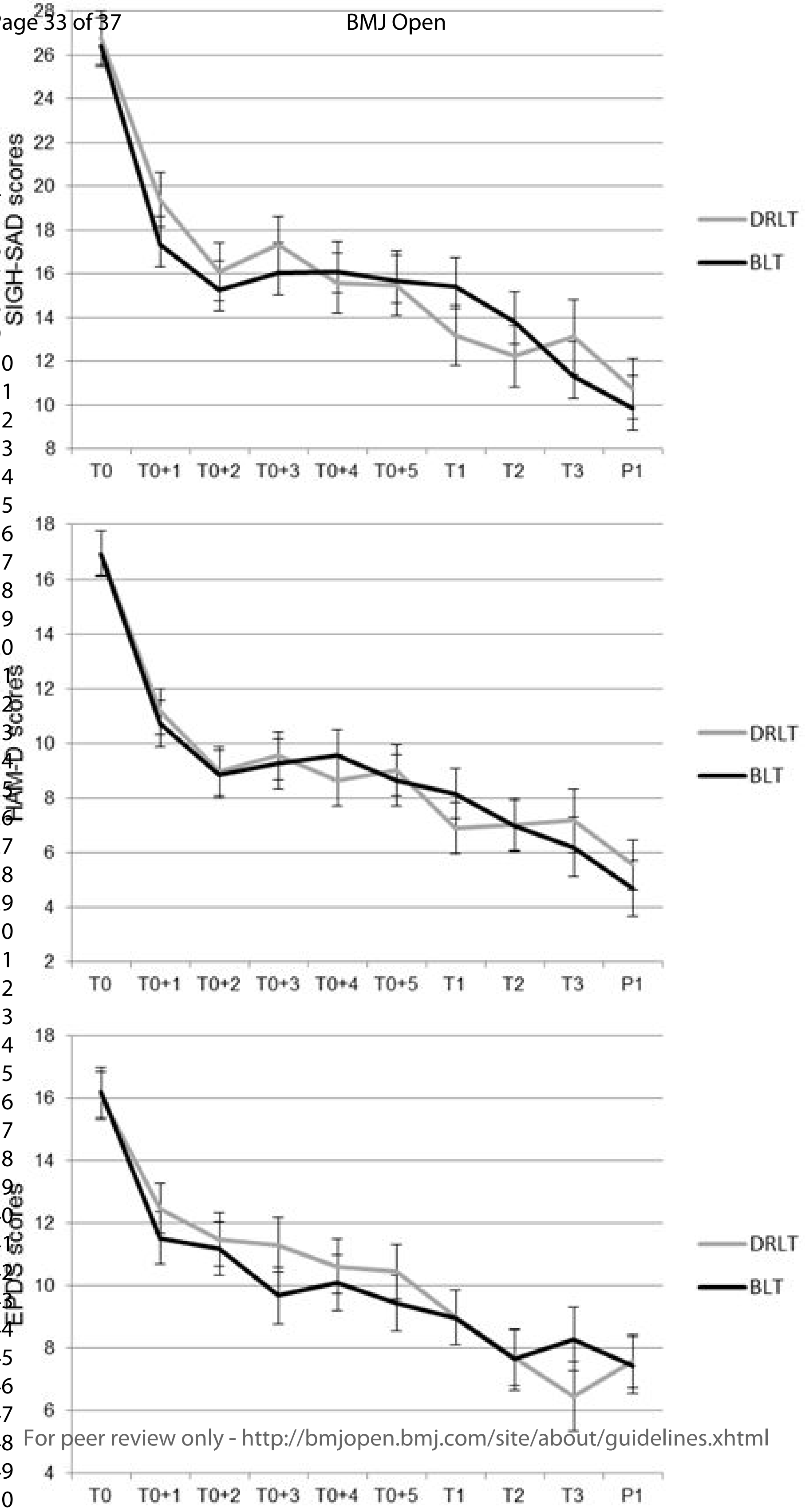
SIGH-SAD scores
DRLT
BLT
T0
T0+1
T0+2
T0+3
T0+4
T0+5
T1
T2
T3
P1
HAM-D scores
EPDS scores

Supplementary Table 1. Photobiological characterizations of light therapy in both treatment arms.

	BLT	DRLT
Cyanopic irradiance (μW · cm^{-2})	578.7	2.24
Melanopic irradiance (μW · cm^{-2})	891	5.53
Chloropic irradiance (μW · cm^{-2})	1032.3	7.23
Erythropic irradiance (μW · cm^{-2})	1212.3	11.37
Rhodopic irradiance (μW · cm^{-2})	16.61	16.61

BLT = bright light therapy; DRLT = dim red light therapy

Supplementary Table 2. Observed median SIGH-SAD, HAM-D and EPDS scores with ranges and number of participants over the course of the study for both treatment arms.

Measure	T0	T0+1	T0+2	T0+3	T0+4	T0+5	T1	T2	T3	P1
**SIGH-SAD**										
**BLT (mdn, range, N)**	27 (14-44; 33)	16.5 (1-33; 30)	16 (2-43; 29)	15 (0-41; 25)	18 (0-32; 25)	17.5 (1-37; 24)	15.5 (0-29; 26)	13 (1-26; 25)	11 (0-29; 17)	8 (1-23; 20)
**DRLT (mdn, range, N)**	26.5 (13-42; 34)	19 (8-33; 31)	17 (2-35; 27)	18 (4-30; 29)	15 (3-28; 24)	16 (2-31; 25)	13 (2-34; 25)	11.5 (1-26; 24)	9.5 (1-31; 14)	8 (0-28; 25)
**HAM-D**										
**BLT (mdn, range, N)**	16 (7-29; 33)	9 (0-25; 30)	9 (1-30; 29)	8 (0-28; 25)	10 (0-22; 25)	10 (0-21; 24)	7.5 (0-20; 26)	8 (0-17; 25)	5 (0-16; 17)	3 (0-11; 20)
**DRLT (mdn, range, N)**	18 (4-29; 34)	10 (3-20; 31)	9 (1-22; 27)	9 (2-20; 29)	8 (0-18; 24)	8 (1-20; 25)	6 (1-18; 25)	4.5 (0-20; 24)	4 (0-15; 14)	4 (0-19; 25)
**EPDS**										
**BLT (mdn, range, N)**	16 (7-25; 31)	11 (3-23; 26)	11 (0-23; 26)	10 (0-19; 21)	8 (0-25; 23)	7 (0-18; 23)	9.5 (1-18; 26)	8.5 (0-15; 18)	8.5 (1-24; 16)	7 (0-13; 22)

For peer review only

**DRLT (mdn, range, N)**	16 (3-25; 34)	12 (6-19; 28)	12 (3-20; 25)	11.5 (3-21; 24)	10 (1-18; 24)	10 (2-19; 23)	6.5 (1-22; 24)	6 (0-21; 23)	4 (1-10; 12)	7 (0-18; 26)

BLT = bright light therapy; DRLT = dim red light therapy; SIGH- SAD = Structured Interview Guide for the Hamilton Depression Scale – Seasonal Affective Disorder version; HAM-D = Hamilton Rating Scale for Depression; EPDS = Edinburgh Postnatal Depression Scale; T0 = baseline, before treatment; T0+1, T0+2 ... T0+5 = weeks during intervention period; T1 = end of treatment; T2 = 3 weeks after end of treatment; T3 = 10 weeks after end of treatment; P1 = 2 months postpartum; mdn = median

Supplementary Table 3. Effects of allocation on the course of depressive symptoms through the intervention period and follow-up (until two months postpartum): sensitivity analyses.

	**β (95% CI) of intervention***	**β (95% CI) of follow-up****
**Adjusted analysis[a]**		
**SIGH-SAD**	-0.27 (-1.70, 1.15)	-0.24 (-1.68, 1.20)
**HAM-D**	0.10 (-0.51, 0.72)	0.13 (-0.49, 0.75)
**EPDS**	0.27 (-0.36, 0.90)	0.25 (-0.38, 0.89)
**Data imputation[b]**		
**SIGH-SAD**	-0.45 (-1.44, 0.53)	-0.08 (-0.63, 0.46)
**HAM-D**	-0.09 (-0.63, 0.44)	0.06 (-0.25, 0.37)
**EPDS**	0.19 (-0.30, 0.68)	0.04 (-0.24, 0.32)
**Post-hoc analysis: high treatment compliance[c]**		
**SIGH-SAD**	-0.40 (-1.36, 0.55)	-0.32 (-0.88, 0.24)
**HAM-D**	-0.12 (-0.79, 0.54)	-0.06 (-0.43, 0.31)
**EPDS**	0.03 (-0.58, 0.65)	-0.05 (-0.40, 0.30)

For peer review only

**Post-hoc analysis: high symptom severity[d]**		
**SIGH-SAD**	-0.84 (-2.33, 0.65)	-0.20 (-1.14, 0.75)
**HAM-D**	-0.16 (-1.12, 0.87)	0.13 (-0.48, 0.73)
**EPDS**	-0.05 (-0.92, 0.82)	0.20 (-0.33, 0.74)

* From start of study until end of treatment; ** From start of study until follow-up 2 months postpartum; [a] Propensity score composed of psychiatric history, ethnicity, level of education, an unplanned pregnancy, maternal age, parity, gestational age, duration of actual depression and other psychiatric or psychotherapeutic treatment interventions; [b] Last observation carried forward; [c] <7 missed treatments; [d] Based on median split baseline SIGH-SAD scores

For peer review only

CONSORT 2010 checklist of information to include when reporting a randomised trial*

Section/Topic	Item No	Checklist item	Reported on page No
**Title and abstract**			
	1a	Identification as a randomised trial in the title	p. 1
	1b	Structured summary of trial design, methods, results, and conclusions (for specific guidance see CONSORT for abstracts)	p. 2
**Introduction**			
Background and objectives	2a	Scientific background and explanation of rationale	p. 4-5
	2b	Specific objectives or hypotheses	p. 5
**Methods**			
Trial design	3a	Description of trial design (such as parallel, factorial) including allocation ratio	p. 5
	3b	Important changes to methods after trial commencement (such as eligibility criteria), with reasons	p. 6
Participants	4a	Eligibility criteria for participants	p. 6; Table 1
	4b	Settings and locations where the data were collected	p. 6
Interventions	5	The interventions for each group with sufficient details to allow replication, including how and when they were actually administered	p. 7-8
Outcomes	6a	Completely defined pre-specified primary and secondary outcome measures, including how and when they were assessed	p. 8-11
	6b	Any changes to trial outcomes after the trial commenced, with reasons	n.a.
Sample size	7a	How sample size was determined	p. 6
	7b	When applicable, explanation of any interim analyses and stopping guidelines	n.a.
Randomisation:			
Sequence generation	8a	Method used to generate the random allocation sequence	p. 9
	8b	Type of randomisation; details of any restriction (such as blocking and block size)	p. 9
Allocation concealment mechanism	9	Mechanism used to implement the random allocation sequence (such as sequentially numbered containers), describing any steps taken to conceal the sequence until interventions were assigned	p. 7-9
Implementation	10	Who generated the random allocation sequence, who enrolled participants, and who assigned participants to interventions	p. 9
Blinding	11a	If done, who was blinded after assignment to interventions (for example, participants, care providers, those	p. 7

For peer review only

		assessing outcomes) and how	
	11b	If relevant, description of the similarity of interventions	p. 7-8
Statistical methods	12a	Statistical methods used to compare groups for primary and secondary outcomes	p. 11-12
	12b	Methods for additional analyses, such as subgroup analyses and adjusted analyses	p. 11-12
**Results**			
Participant flow (a diagram is strongly recommended)	13a	For each group, the numbers of participants who were randomly assigned, received intended treatment, and were analysed for the primary outcome	p. 12, Fig. 1, Supp. Table 2
	13b	For each group, losses and exclusions after randomisation, together with reasons	Fig. 1
Recruitment	14a	Dates defining the periods of recruitment and follow-up	p. 6
	14b	Why the trial ended or was stopped	p. 6
Baseline data	15	A table showing baseline demographic and clinical characteristics for each group	Table 2
Numbers analysed	16	For each group, number of participants (denominator) included in each analysis and whether the analysis was by original assigned groups	Sup. Table 2
Outcomes and estimation	17a	For each primary and secondary outcome, results for each group, and the estimated effect size and its precision (such as 95% confidence interval)	Sup. Table 2, Fig. 2, p.13-15, Table 3
	17b	For binary outcomes, presentation of both absolute and relative effect sizes is recommended	p. 14
Ancillary analyses	18	Results of any other analyses performed, including subgroup analyses and adjusted analyses, distinguishing pre-specified from exploratory	p. 14, Sup. Table 3
Harms	19	All important harms or unintended effects in each group (for specific guidance see CONSORT for harms)	p. 14
**Discussion**			
Limitations	20	Trial limitations, addressing sources of potential bias, imprecision, and, if relevant, multiplicity of analyses	p. 17-18
Generalisability	21	Generalisability (external validity, applicability) of the trial findings	p. 15-17
Interpretation	22	Interpretation consistent with results, balancing benefits and harms, and considering other relevant evidence	p. 15-17
**Other information**			
Registration	23	Registration number and name of trial registry	p. 2, 6
Protocol	24	Where the full trial protocol can be accessed, if available	p. 6
Funding	25	Sources of funding and other support (such as supply of drugs), role of funders	p. 19

*We strongly recommend reading this statement in conjunction with the CONSORT 2010 Explanation and Elaboration for important clarifications on all the items. If relevant, we also recommend reading CONSORT extensions for cluster randomised trials, non-inferiority and equivalence trials, non-pharmacological treatments, herbal interventions, and pragmatic trials. Additional extensions are forthcoming: for those and for up to date references relevant to this checklist, see www.consort-statement.org.

For peer review only - http://bmjopen.bmj.com/site/about/guidelines.xhtml
